# Large multi-ethnic genetic analyses of amyloid imaging identify new genes for Alzheimer disease

**DOI:** 10.1186/s40478-023-01563-4

**Published:** 2023-04-26

**Authors:** Muhammad Ali, Derek B. Archer, Priyanka Gorijala, Daniel Western, Jigyasha Timsina, Maria V. Fernández, Ting-Chen Wang, Claudia L. Satizabal, Qiong Yang, Alexa S. Beiser, Ruiqi Wang, Gengsheng Chen, Brian Gordon, Tammie L. S. Benzinger, Chengjie Xiong, John C. Morris, Randall J. Bateman, Celeste M. Karch, Eric McDade, Alison Goate, Sudha Seshadri, Richard P. Mayeux, Reisa A. Sperling, Rachel F. Buckley, Keith A. Johnson, Hong-Hee Won, Sang-Hyuk Jung, Hang-Rai Kim, Sang Won Seo, Hee Jin Kim, Elizabeth Mormino, Simon M. Laws, Kang-Hsien Fan, M. Ilyas Kamboh, Prashanthi Vemuri, Vijay K. Ramanan, Hyun-Sik Yang, Allen Wenzel, Hema Sekhar Reddy Rajula, Aniket Mishra, Carole Dufouil, Stephanie Debette, Oscar L. Lopez, Steven T. DeKosky, Feifei Tao, Michael W. Nagle, Knight Alzheimer Disease Research Center (Knight ADRC), the Dominantly Inherited Alzheimer Network (DIAN), Alzheimer’s Disease Neuroimaging Initiative (ADNI), ADNI-DOD, A4 Study Team, the Australian Imaging Biomarkers, Lifestyle (AIBL) Study, Timothy J. Hohman, Yun Ju Sung, Logan Dumitrescu, Carlos Cruchaga

**Affiliations:** 1 Department of Psychiatry, Washington University, St. Louis, MO 63110 USA; 2 NeuroGenomics and Informatics, Washington University, St. Louis, MO 63110 USA; 3 Vanderbilt Memory and Alzheimer’s Center, Vanderbilt University School of Medicine, Nashville, TN USA; 4 Glenn Biggs Institute for Alzheimer’s and Neurodegenerative Diseases, UT Health, San Antonio, TX 78229 USA; 5 Department of Biostatistics, Boston University School of Public Health, Boston, MA USA; 6 Department of Neurology, Boston University School of Medicine, Boston, MA USA; 7 Boston University, Boston, MA USA; 8 Knight Alzheimer’s Disease Research Center, Washington University, St Louis, MO USA; 9 Mallinckrodt Institute of Radiology, Washington University, St Louis, MO USA; 10 Department of Neurology, Washington University, St Louis, MO USA; 11 Department of Neuroscience, Ronald M. Loeb Center for Alzheimer’s Disease, Icahn School of Medicine at Mount Sinai, New York, NY USA; 12 Framingham Heart Study, Framingham, MA USA; 13 Boston University School of Medicine, Boston, MA USA; 14 The Department of Neurology, Columbia University, New York, NY USA; 15 Department of Neurology, Harvard Medical School, Boston, MA USA; 16 Brigham and Women’s Hospital and Department of Neurology, Massachusetts General Hospital, Harvard Medical School, Boston, MA USA; 17 Athinoula A. Martinos Center for Biomedical Imaging, Charlestown, MA USA; 18 Massachusetts General Hospital, Harvard Medical School, Boston, MA USA; 19 Department of Digital Health, Samsung Medical Center, SAIHST, Sungkyunkwan University, Seoul, Republic of Korea; 20 Department of Neurology, Dongguk University Ilsan Hospital, Dongguk University College of Medicine, Goyang, Republic of Korea; 21 Department of Neurology, Samsung Medical Center, Sungkyunkwan University School of Medicine, Seoul, Republic of Korea; 22 Department of Neurology and Neurological Sciences, Stanford University, Stanford, CA USA; 23 Centre for Precision Health, Edith Cowan University, 270 Joondalup Dr, Joondalup, WA 6027 Australia; 24 Department of Human Genetics, University of Pittsburgh, Pittsburgh, PA USA; 25 Department of Radiology, Mayo Clinic-Minnesota, Rochester, MN 55905 USA; 26 Department of Neurology, Mayo Clinic-Minnesota, Rochester, MN 55905 USA; 27 Department of Neurology, Brigham and Women’s Hospital, Boston, MA USA; 28 Department of Neurology, Massachusetts General Hospital, Boston, MA USA; 29 Harvard Medical School, Boston, MA USA; 30 Broad Institute of Harvard and MIT, Cambridge, USA; 31 Wisconsin Alzheimer’s Institute, Madison, WI USA; 32 UMR 1219, University of Bordeaux, INSERM, Bordeaux Population Health Research Centre, Team ELEANOR, 33000 Bordeaux, France; 33 Department of Neurology, Boston University School of Medicine, Boston, MA 2115 USA; 34 Department of Neurology, CHU de Bordeaux, 33000 Bordeaux, France; 35 Department of Neurology, University of Pittsburgh, Pittsburgh, PA USA; 36 Department of Neurology and McKnight Brain Institute, University of Florida, Gainesville, FL USA; 37 Neurogenomics, Genetics-Guided Dementia Discovery, Eisai, Inc, Cambridge, MA USA; 38 Hope Center for Neurologic Diseases, Washington University, St. Louis, MO 63110 USA; 39 Department of Genetics, Washington University School of Medicine, St Louis, MO 63110 USA

**Keywords:** Brain amyloidosis, Amyloid PET, Alzheimer’s disease, Multi-ethnic, Meta-analysis, GWAS

## Abstract

Amyloid PET imaging has been crucial for detecting the accumulation of amyloid beta (Aβ) deposits in the brain and to study Alzheimer’s disease (AD). We performed a genome-wide association study on the largest collection of amyloid imaging data (N = 13,409) to date, across multiple ethnicities from multicenter cohorts to identify variants associated with brain amyloidosis and AD risk. We found a strong *APOE* signal on chr19q.13.32 (top SNP: *APOE* ɛ4; rs429358; β = 0.35, SE = 0.01, P = 6.2 × 10^–311^, MAF = 0.19), driven by *APOE* ɛ4, and five additional novel associations (*APOE* ε2/rs7412; rs73052335/rs5117, rs1081105, rs438811, and rs4420638) independent of *APOE* ɛ4. *APOE* ɛ4 and ε2 showed race specific effect with stronger association in Non-Hispanic Whites, with the lowest association in Asians. Besides the *APOE*, we also identified three other genome-wide loci: *ABCA7* (rs12151021/chr19p.13.3; β = 0.07, SE = 0.01, *P* = 9.2 × 10^–09^, MAF = 0.32), *CR1* (rs6656401/chr1q.32.2; β = 0.1, SE = 0.02, P = 2.4 × 10^–10^, MAF = 0.18) and *FERMT2* locus (rs117834516/chr14q.22.1; β = 0.16, SE = 0.03, *P* = 1.1 × 10^–09^, MAF = 0.06) that all colocalized with AD risk. Sex-stratified analyses identified two novel female-specific signals on chr5p.14.1 (rs529007143, β = 0.79, SE = 0.14, P = 1.4 × 10^–08^, MAF = 0.006, sex-interaction P = 9.8 × 10^–07^) and chr11p.15.2 (rs192346166, β = 0.94, SE = 0.17, P = 3.7 × 10^–08^, MAF = 0.004, sex-interaction P = 1.3 × 10^–03^). We also demonstrated that the overall genetic architecture of brain amyloidosis overlaps with that of AD, Frontotemporal Dementia, stroke, and brain structure-related complex human traits. Overall, our results have important implications when estimating the individual risk to a population level, as race and sex will needed to be taken into account. This may affect participant selection for future clinical trials and therapies.

**Supplementary Information:**

The online version contains supplementary material available at 10.1186/s40478-023-01563-4.

## Introduction

Alzheimer’s disease (AD) is a complex polygenic disease with a genetic heritability estimated to be 58–79% [1]. This high genetic heritability in AD provides the opportunity to perform large-scale genetic studies in order to characterize new biological features, identify relevant pathophysiological processes, and establish novel diagnostic biomarkers for early detection. Recent genome-wide association studies (GWAS) have identified more than 74 AD risk loci, including the *APOE* ɛ4 locus, implicating various biological process e.g., amyloid processing and innate immunity in the development of AD [2–5]. However, known common AD variants account only for an approximately 30% of the AD genetic variance [6] and a large proportion of the underlying heritability still remains unexplained.

Although most of the genetic studies on AD are focused on clinical diagnosis as the primary outcome, using quantitative endophenotypes can also be helpful in identifying additional AD-related genes. Two such AD-related endophenotypes are accumulation of amyloid-beta (Aβ) in the brain and the formation of tau deposits in the form of neurofibrillary tangles and dystrophic neurites (tau pathology) [7]. Different cross-sectional and longitudinal studies on cognitively normal subjects have also implicated amyloidosis as an early process in AD pathology [8–10]. The *in-vivo* detection of Aβ accumulation in the brain, as measured by positron emission tomographic (PET) imaging tracers such as 11C-labeled Pittsburgh Compound-B (PiB) [11] and 18F-AV-45 (Florbetapir) [12] has provided a biomarker for AD diagnosis and risk assessment. Development of this advanced brain imaging approach has enabled the detection of fibrillar Aβ before the onset of symptoms, providing avenues for characterizing new genetic risk factors, and to design novel therapeutic approaches to halt the early progression of disease.

Previous genetic investigations leveraging amyloid PET imaging data as AD endophenotype have established its association with *APOE* locus [11–14]. In a recent multi-center case–control based study (N = 4314) using amyloid PET as a quantitative trait [15], a novel locus was reported to be associated with brain amyloidosis within *RBFOX1* gene. However, clinical heterogeneity across these studies and their limited sample size demands further investigations to replicate and expand on these findings. Here, we systematically analyzed the largest collection of amyloid imaging data (N = 13,409), across multiple ethnicities from multicenter cohorts (Knight ADRC, A4, DIAN, ADNI, ADNIDOD, UPitt, HABS, AIBL, Memento, MCSA, WRAP, Berkeley, Korean study, and MISSION-AD) as a quantitative trait to identify the functional variants and genes driving the association of AD. Furthermore, we have conducted gender-, and *APOE*-stratified analyses to investigate the effect of these variables on brain amyloidosis.

## Methods

### Study samples and amyloid-PET harmonization

For this study, we collected data from 14 different cohorts with a total sample size of 13,409 participants (Table 1). We analyzed the association of common and low frequency (MAF > 0.0005) genetic variants with amyloid-PET imaging, which is a well-known and validated AD endophenotype, serving as biomarker for brain amyloidosis. These subjects were recruited from the Memory and Aging Project (MAP) at the Knight Alzheimer Disease Research Center (Knight-ADRC) [16, 17], Alzheimer's Disease Neuroimaging Initiative (ADNI) [18], the Dominantly Inherited Alzheimer Network (DIAN) [19], Anti-Amyloid Treatment in Asymptomatic Alzheimer's Disease (A4) [20], ADNI Department of Defense (ADNIDOD) studies, Australian Imaging, Biomarkers and Lifestyle (AIBL) [21], The Harvard Aging Brain Study (HABS) [22], and University of Pittsburgh (UPitt) [23]. Furthermore, summary statistics data was obtained from six additional cohorts that processed the raw genotype and phenotype data according to the same pipeline. These cohorts included Memento [24], Mayo Clinic Study of Aging (MCSA) [25], Wisconsin Registry for Alzheimer's Prevention (WRAP) [26], Berkeley Aging Cohort study (BACS) [27], Korean study [28], and MISSION-AD. Part of the data used in the preparation of this article was obtained from the ADNI database (adni.loni.usc.edu). Collection of genotype data and PET image processing for each cohort are described in detail in the respective studies [16–23, 25, 27–29].

**Table 1:** Demographics of amyloid PET GWAS participants at the time of scanning. This table summarizes basic demographic information of participants included in the analysis. For each cohort, we report number of participants, percentage of females and males, mean age of the participants and standard deviation (SD) in the age, percentage of *APOE* ε4-carriers (*APOE* ε4 + participants), and percentage of cases and control participants, where available. To normalize amyloid PET endophenotype across different cohorts, we converted different amyloid imaging measures (e.g., Centiloid, PiB, and AV45) into log-normalized z-score using “scale” function in base R. Phenotype from each cohort was normalized individually to account for within cohort variation. Abbreviations: PET, positron emission tomography; sd, standard deviation; Knight-ADRC, Knight Alzheimer’s Disease Research Center; ADNI, Alzheimer's Disease Neuroimaging Initiative; DIAN, the Dominantly Inherited Alzheimer Network; A4, Anti-Amyloid Treatment in Asymptomatic Alzheimer's Disease; ADNI-DOD, ADNI Department of Defense studies; AIBL, Australian Imaging, Biomarkers and Lifestyle; HABS, The Harvard Aging Brain Study; UPitt, University of Pittsburgh; MCSA, Mayo Clinic Study of Aging; WRAP, Wisconsin Registry for Alzheimer's Prevention; BACS, Berkeley Aging Cohort study; SUVR, standardize uptake value ratios; FBP, Florbetapir; PIB, Pittsburgh Compound-B; FMT, Flutemetamol

Variables	Total	A4	ADNI	AIBL	ADNI-DOD	DIAN	HABS	Knight-ADRC	UPitt	Korean	Memento	MCSA	WRAP	BACS	MISSION-AD
Tracer	–	SUVR	FBP	FBP/PIB/FMT	AV45	PIB	PIB	PIB/AV45	PIB	AV45	FBP/FMT	PIB	PIB	PIB	FBP/FBB/ FMT
Total	13,409	3,180	1,134	1,214	169	209	258	1,048	345	996	630	1712	78	173	2,263
Female (%)	48.52	60.09	47	54.04	0.59	52.63	59.3	54.77	45.8	56.9	59.25	46.3	35	59	48.6
Male (%)	51.48	39.91	53	45.96	99.41	47.37	40.7	45.23	54.2	43.1	40.75	53.7	65	41	51.4
Age (mean)	69.63	71.30	73.73	72.65	69.08	36.60	73.99	70.74	78.39	70.15	71.55	73.4	67	74.49	71.8
Age (sd)	7.65	4.73	7.62	6.62	4.67	10.86	6.1	9.01	9.91	8.72	8.15	10.6	6.02	6.30	7.8
*APOE*4 + (%)	35.06	36.04	43.39	35.01	26.63	28.71	29.84	41.13	32.46	41.7	28.65	28.6	42	28	48.7
Cases (%)	35.17	0.06	61.55	7.5	32.54	45.01	27.13	16.98	12.17	69.8	100	15.7	5	0	99
Controls (%)	60.69	99.94	32.36	79.74	67.46	38.28	72.87	61.83	87.83	30.2	0	84.1	95	100	0

Briefly, individuals were diagnosed as cognitively healthy (controls) or clinical AD (cases), based on their Clinical Dementia Rating® (CDR®) that was available for 86% of the complete dataset. The CDR is a scaling system that categorizes the overall dementia severity for each participant into five classes (no dementia = 0, very mild = 0.5, mild = 1, moderate = 2, and severe = 3). For this study, individuals with CDR = 0 were considered as controls and the remaining were classified as cases. Participants were included if they had measurements of raw amyloid-PET levels and corresponding genotype data. Any participant that was missing information about the sex, age, and genetic principal components (PCs), was excluded from the study.

For standardization, amyloid PET data from each cohort was normalized to their reference cerebellar regions in order to obtain standardized uptake value ratios (SUVR) in a composite of cortical brain areas. As different cohorts obtained quantitative amyloid PET data using different tracers (e.g., PIB, FBP, and AV45), the available raw phenotypic data cannot combine unless it is normalized in advance. To normalize amyloid PET endophenotype across different cohorts, we converted different amyloid imaging measures into log-normalized z-score using “scale” function in base R. Briefly, z-scores were calculated by using the mean and standard deviation (SD) units across each cohort and applied to the entire endophenotype in order to account for within cohort variation. Samples having normalized z-score 3-SD away from the mean of the population were considered as outliers and removed from the subsequent analyses.

For seven additional cohorts (Memento, MCSA, WRAP, BACS, Korean study, and MISSION-AD), the raw phenotype and genetic data was not accessible due to the strict patient data sharing policies (Table 1). In those particular cases, the summary statistics data was obtained where association analyses were performed using the same analytical pipeline.

### Genotyping, imputation, quality control, and population structure

The genotyping platforms used by each cohort are listed in the respective studies [16–23, 25, 27–29]. All the GWAS datasets were aligned to GRCh38. For phasing and imputation of non-genotyped single-nucleotide polymorphisms (SNPs), we used the TOPMed Imputation Server (https://imputation.biodatacatalyst.nhlbi.nih.gov/#!). Phasing was performed using eagle v2.4 [30] and only those variants having imputation quality (Rsq or estimated R^2^) of 0.3 or greater were retained [31]. Genotyped and imputed variants with minor allele frequency (MAF) < 0.0002 were removed. We applied stringent quality control (QC) filters to process the genotyping array and sequencing data. We used the threshold of 98% for removing single nucleotide polymorphisms (SNPs) and individuals with low call rate. Autosomal SNPs that were not in the Hardy–Weinberg equilibrium (P = 1 × 10^−6^) were also removed. Subject duplication and relatedness were estimated from identity-by-descent (IBD) analysis carried out in Plink version 2.0 [32]. In case of related subjects (Pihat ≥ 0.25), the sample from the Knight ADRC or with a higher number of variants that passed the QC was prioritized. We performed Principal component analysis (PCA) on the genotype data to obtain genetic PCs that capture population substructure. In order to evaluate the association of genetic variants with brain amyloidosis in different populations, we considered a homogenous pool of three ethnicities (Non-Hispanic whites, American African, and Asian) for the subsequent statistical analyses (Additional file 1: Fig. S1).

After QC of genotype and phenotype data, a total of 7557 participants remained available from eight different cohorts for the subsequent analyses. According to the genetic PCA, participants were determined to be Non-Hispanic Whites (NHW; n = 7036), American Africans (AFR; n = 359), and Asians (ASN; n = 162). Additionally, summary statistics data was leveraged from six external amyloid PET cohorts to further increase the study sample size for NHW (n = 11,556) and ASN (n = 1494) ancestries (Additional file 1: Table S1a and S1b). The demographic characteristics of all the study participants (n = 13,409) from each of the 14 datasets included in the multi-ethnic standard error (StdErr)-based meta-analysis are shown in Table 1. The distribution of standardized amyloid-PET levels (Z-score) across eight cohorts is shown in Additional file 1: Fig. S2. Overall, the participants ranged from age 37 to 78 with a mean age of 69 across all cohorts. The age of participants from the DIAN cohort was almost half the mean age of all cohorts and nearly all the samples in the ADNI-DOD cohort were males. Around one-third (34%) of the individuals were *APOE* ε4 carriers. The number of cases and controls samples were 23% and 63%, respectively, and 14% of the individuals were missing the clinical diagnosis. There was an even distribution of gender across all cohorts with a total of 48% female and 52% male participants.

### Statistical analyses

Statistical analyses and data visualization were performed in Plink version 2.0 [32], Metal [33], and R version 3.5.2 [34]. We performed association analyses of common and low frequency variants with quantitative AD endophenotype from PET scan (Aβ) using an additive model and included sex, age and the 10 principal components as covariates, within each ethnicity. Within each ethnicity we first performed joint analyses using the Z-scores for those cohorts for which we had access to the genetic and amyloid PET data (Additional file 1: Fig. S3). Then we performed meta-analyses using METAL [33], of joint-cohorts with those that only had summary statistics (Memento, MCSA, WRAP, BACS, Korean study, and MISSION-AD) to obtain the race-specific meta-analyses. To conduct the multi-ethnic meta-analysis, a standard-error (StdErr)-based approach was used from Metal [33]. The variant-level visualization was accomplished using LocusZoom [30] tool.

As the amyloid PET imaging was calculated using different traces and pipelines, the joint analyses could lead to false positive or negative findings. Similarly, because of the different distribution of the amyloid PET data, the Z-scores could lead to spurious results. Existing studies suggest joint analysis can be more powerful than meta-analysis for low frequency variants where dataset is comprised of divergent samples [35, 36]. On the other hand, meta-analysis approaches do not require the complete original data as they can be used with summary statistics information while accounting for difference in population size, and they are less demanding computationally [37]. In order to confirm that the Z-score normalization and the joint analyses lead to robust results, we performed a GWAS analyses using raw centiloid values and the Z-scores for the Knight-ADRC and ADNI (Additional file 1: Fig. S4). A very strong correlation in p-values and effect size (R > 0.89) was found in these comparisons indicating that Z-scores will lead to the same signals as the raw amyloid data. In addition to the above validation, we performed a more systematic analyses comparing the *p*-values and effect sizes of the joint (z-scores) and the meta-analysis for all cohorts for those having raw phenotypic data available (A4 = 3,180, ADNI = 1134, ADNIDOD = 169, AIBL = 1214, DIAN = 209, HABS = 258, Knight-ADRC = 1,048, UPitt = 345). Briefly, (1) the raw phenotype values were converted to log10 scale for each cohort and each cohort was analyzed independently. Then the cohort-specific results were combined by meta-analyses. For meta-analysis, we used METAL software and standard-error (SE)-based meta-analysis approach. (2) In parallel, the raw phenotypes were also converted to z-score, separately for each cohort, combined by performing a single joint analyses. (3) Finally, the *p*-values and effect sizes of associations were compared between these two analyses (joint vs. meta-analysis). We assessed the correlation of effect sizes and p-values at different *p*-values thresholds of associations (P = 0.05, 5 × 10^–05^, and 5 × 10^–08^) and found very strong correlation (R > 0.94) in all comparisons (Additional file 1: Fig. S5). This high correlation indicates that the joint and the meta-analyses lead to the same results.

We also performed *APOE* ε4-, sex- and case–control status-stratified analyses. Most of these analyses were performed only on those datasets for which amyloid PET and genetic data were available. Therefore, the samples sizes for those analyses were lower than for the overall analyses. We also compared the *p*-values of all these analyses using the z-scores joint vs the meta-analysis (Additional file 1: Fig. S6, S7 and Table S2), finding very high correlation.

In order to compare the effect size (BETA) across different ethnicities, we used two-sample t-test that takes into account the BETA and standard error (SE) for performing a pair-wise comparison and provide the *p*-value of significance whether difference between BETA is statistically significant or not. We developed a custom R function that uses BETA and square root of SE^2^ of two groups to obtain a Z-score. As this Z-score follows a normal distribution with a sample size > 100 in each ancestry, we obtained a *p*-value for significance by using the pnorm function in R.

### Post-GWAS analyses

**Fig. 1:**
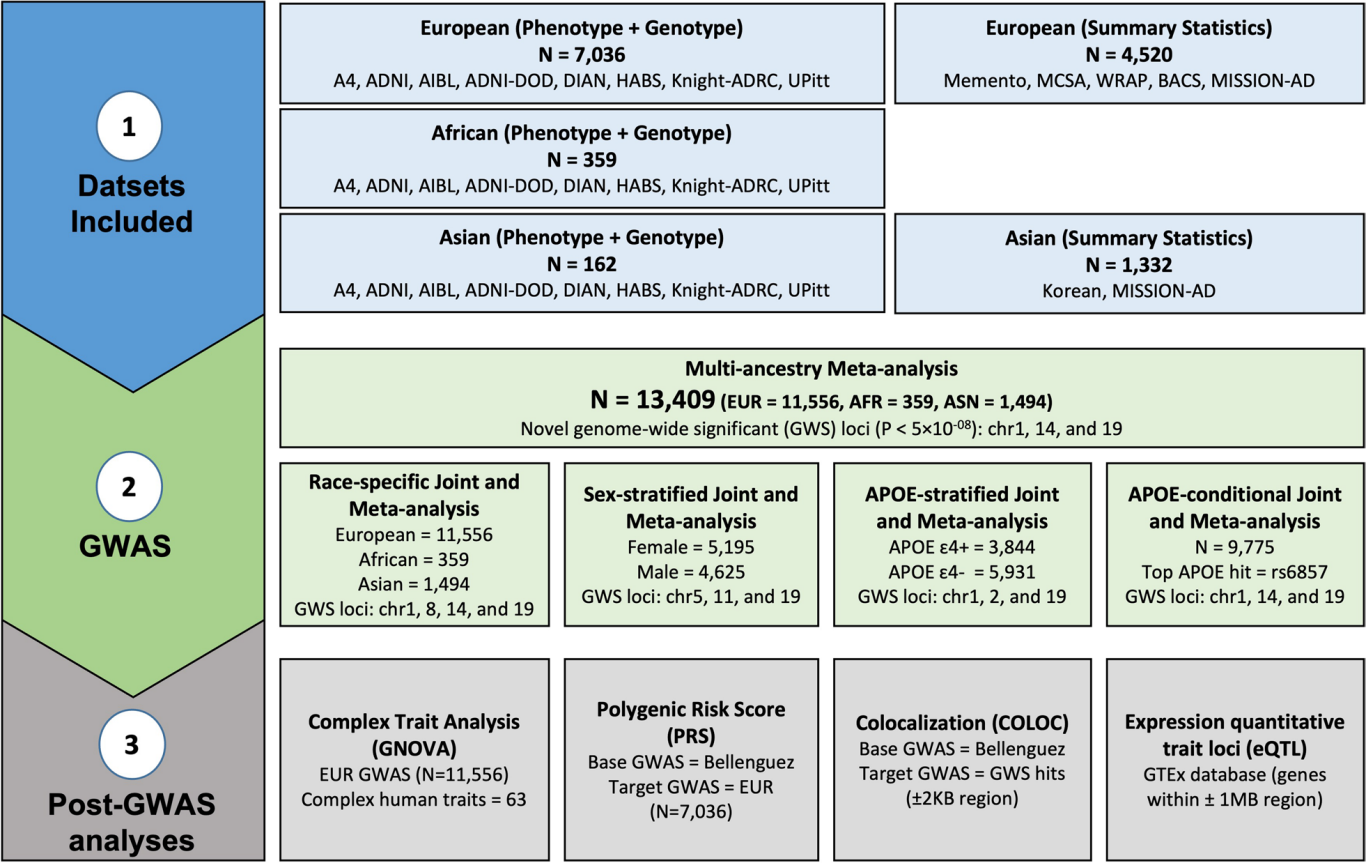
Schematic overview of datasets and performed analyses. Amyloid PET endophenotype and corresponding genotype data was available for 8 different cohorts with a total sample size of 7,557 (NHW = 7036, African = 359, Asian = 162). We also got GWAS summary statistics data from 6 external cohorts having a total sample size of 5852 (NHW = 4520, Asian = 1332). We performed Race-specific linear regression using amyloid PET as a quantitative endophenotype and age, sex, cohort name, and first ten genetic PCs as model covariates. The same analytic pipeline was used by the external cohorts for generating the summary statistics data. We meta-analyzed the results from internal and external summary statistics using a standard error (StdErr)-based meta-analysis approach using METAL software (N = 13,409). Furthermore, different post-GWAS analyses were carried out to identify novel SNPs associated with brain amyloidosis

Multiple post-GWAS analyses were conducted for the functional annotation of the identified hits. A schematic overview of study design and conducted analyses is provided in Fig. 1.

#### Genetic covariance analysis

We used Genetic Covariance Analyzer (GNOVA) program [38] for assessing the genetic covariance and correlation of amyloid PET AD endophenotype with different complex human traits. Publicly accessible GWAS summary statistics data was downloaded for 63 different complex human traits. Specific details about the sample size and source of summary statistics for each trait are presented in Additional file 1: Table S3. The obtained summary statistics data was used to assess the genetic covariance of each trait with amyloid PET GWAS from non-Hispanic Whites (NHW) ancestry (N = 11,556). The tool (GNOVA) that has been used for calculating genetic correlation has the inherent ability to take the sample overlap between different GWAS into account. The algorithm allows random error terms ϵ and δ to be correlated in order to account for the non-genetic correlation introduced by sample overlapping between two GWAS.

#### Bayesian co-localization analysis

We performed co-localization analysis using COLOC [39] tool (version 5.2.1). For each genomic locus of interest, identified by multi-ethnic or sex-stratified amyloid PET GWAS analyses, co-localization was assessed against AD risk GWAS from Bellenguez et al. [5]. Furthermore, to assess the genotype-specific expression of identified SNPs in human brain tissues, we performed cis-expression quantitative trait loci (QTL) analysis using gene expression data from the Genotype-Tissue Expression (GTEx) portal [40]. For both these analyses, posterior probability was calculated for five different hypotheses, (i) H0: there is no causal variant for either trait in the specified region, (ii) H1: there is a causal variant for trait 1, (iii) H2: there is a causal variant for trait 2, (iv) H3: both traits have distinct causal variants, and (v) H4: there is a single causal variant common to both traits. Here, our hypothesis of interest was H4. In case of co-localization with AD risk, a candidate locus was defined as 500 KB upstream and downstream of the variant of interest, however, in the case of eQTL analysis this region was extended to 1 MB on either side. For eQTL analysis, 10 types of tissues were considered from the GTEx portal, including “Brain Hypothalamus”, “Brain Cerebellar Hemisphere”, “Brain Cerebellum”, “Brain Cortex”, “Brain Amygdala”, “Brain Caudate basal ganglia”, “Brain Nucleus accumbens basal ganglia”, “Brain Anterior cingulate cortex BA24”, “Brain Putamen basal ganglia”, and “Whole Blood”.

#### Polygenic risk score analysis

PRSice-2 software [41] was used for calculating the Polygenic risk score (PRS). We used the summary statistics data from the largest available clinically assessed AD case–control GWAS study [5] (N = 788,989) as the base GWAS. We generated genetic risk scores as the weighted sum of the risk alleles for all participants in the amyloid PET GWAS of NHW ancestry (N = 7036). The standard clumping and thresholding (C + T) approach was used where only those markers are retained that are most strongly associated with the disease. PRS calculation was generated at multiple different thresholds, ranging from 5 × 10^–08^ to 0.5 with an interval of 5 × 10^–05^, on linkage disequilibrium (LD)-clumped SNPs by retaining the SNP with the smallest *p*-value and excluding the variants with r2 > 0.1 in a 250-kb window. We also calculated PRS by excluding the *APOE* region (GRCh38—chr19:43,907,927–45,908,821) because of the high LD in this region.

## Results

### Study design

In this study, we harmonized and integrated brain amyloid imaging and genetic data from 14 different cohorts with a total sample size of 13,409 (Table 1, Fig. 1), from three different populations (Non-Hispanic Whites (NHW), African (AFR) and Asian (ASN)), in order to identify genetic variants associated with amyloidosis and AD risk. The datasets with individual level genetic and brain amyloid imaging data and from the same ethnicity were analyzed using a joint approach after harmonizing the amyloid imaging. Results from these analyses were then meta-analyzed together with the datasets from the same ethnicity for which only summary statistics were available leading to race-specific brain amyloid imaging GWAS. Multi-ethnic GWAS was then performed using a random effect model. Moreover, *APOE*-, sex-, and diagnosis-stratified analyses were performed including those datasets with individual-level data to identify additional loci and genes to further explore the genetic architecture of brain amyloidosis. Functional annotation and expression and protein Quantitative Trait Loci (pQTL) mapping was performed to identify the most likely functional genes driving the associations for the GWAS signals. Finally, the overlap between the genetic architecture of brain amyloid imaging with AD risk and other traits were analyzed using Polygenic Risk Scores (PRS) and genetic covariance analysis.

### GWAS analyses

The race-specific meta-analysis for NWH participants (n = 11,556) from 13 different cohorts yielded a very strong signal in the *APOE* locus. In this locus, rs429358, which codifies for *APOE* ε4, was the most significant SNP (P = 1.8 × 10^–416^, β = 0.62, SE = 0.01, MAF = 0.19, I^2^ = 99.7; Additional file 1: Fig. S8A). Besides the *APOE* locus, we identified two additional novel genome-wide significant signals in chr14q.22.1 (rs117834516, P = 8.7 × 10^–09^, β = 0.15, SE = 0.03, MAF = 0.06, I^2^ = 0) on the *FERMT2 locus* and chr1q.32.2 (rs6656401, P = 9.1 × 10^–10^, β = 0.10, SE = 0.02, MAF = 0.18, I^2^ = 43.1) on the *CR1* locus (Additional file 1: Fig. S9C, D). All these signals colocalized with that of AD risk GWAS [4] having posterior probability of 99% for sharing a single causal variant (Additional file 1: Table S4). Colocalization with eQTL (in multiple brain regions based on GTEx [42]) was also observed for the chr1q.32.2 locus, rs6656401, with *CR1* mRNA levels (PP.H4 = 0.99), the chr14q.22.1, rs117834516, with *STYX* mRNA levels (PP.H4 = 0.93), but not *FERMT2* (PP.H4 = 0.08) (Additional file 1: Table S5, S6). Recent GWAS have provided strong evidence for *CR1* and *FERMT2* being the risk factors for the development of AD [43, 44]. Specifically, *CR1* has been reported to be consistently involved in immune system related pathways, particularly complement and inflammatory cytokines [45]. Changes in the gene expression level of *FERMT2* in the neurons have been shown to effect both extracellular Aβ40 and Aβ42 as well as phospho-Tau [46].

In case of AFR-specific analyses (n = 359), rs429358 was found to be the most significant SNP in the *APOE* locus (P = 1.0 × 10^–11^, β = 0.49, SE = 0.07, MAF = 0.21; Additional file 1: Fig. S8B). In addition, we found a novel locus on chr8q.22.1 (rs2271774, P = 2.5 × 10^–09^, β = 1.10, SE = 0.17, MAF = 0.028) that passed the genome-wide significance. This same SNP has a MAF of 1.3% in ASN (N = 336, P = 0.84, β = − 0.04, SE = 0.19, MAF = 0.013) and 0.21% in NHW (N = 6329, P = 0.84, β = 0.13, SE = 0.56, MAF = 0.0021) but it was not even nominally associated in these two ancestries, suggesting it to be an AFR-specific signal, or a false positive. Additional studies having larger dataset, focused on individuals with AFR background, will be needed to further replicate this association. This SNP is located in the *PTDSS1* gene-region that also includes *GDF6*, *UQCRB*, *MTERF3*, and *SDC2* genes (Additional file 1: Fig. S10A). Although we did not find any association of chr8q.22.1 with gene or protein levels QTLs (Additional file 1: Table S7), we found that *PTDSS1,* but not any other gene in this region, to be significantly differentially expressed in different brain tissues (IFG, PHG, STG, and TCX) with a consistent negative log2 fold change [47], nominating *PTDSS1* as the functional gene in this locus.

In case of ASN ancestry-specific analyses (n = 1,494), no additional genome-wide signals were found besides *APOE* locus (P = 6.7 × 10^–28^, β = 0.14, SE = 0.01, MAF = 0.14; Additional file 1: Fig. S8C). Overall, the rs429358 SNP that codifies for *APOE* ε4 was the most significant SNP across every race-specific GWAS with a consistent positive effect size.

**Fig. 2:**
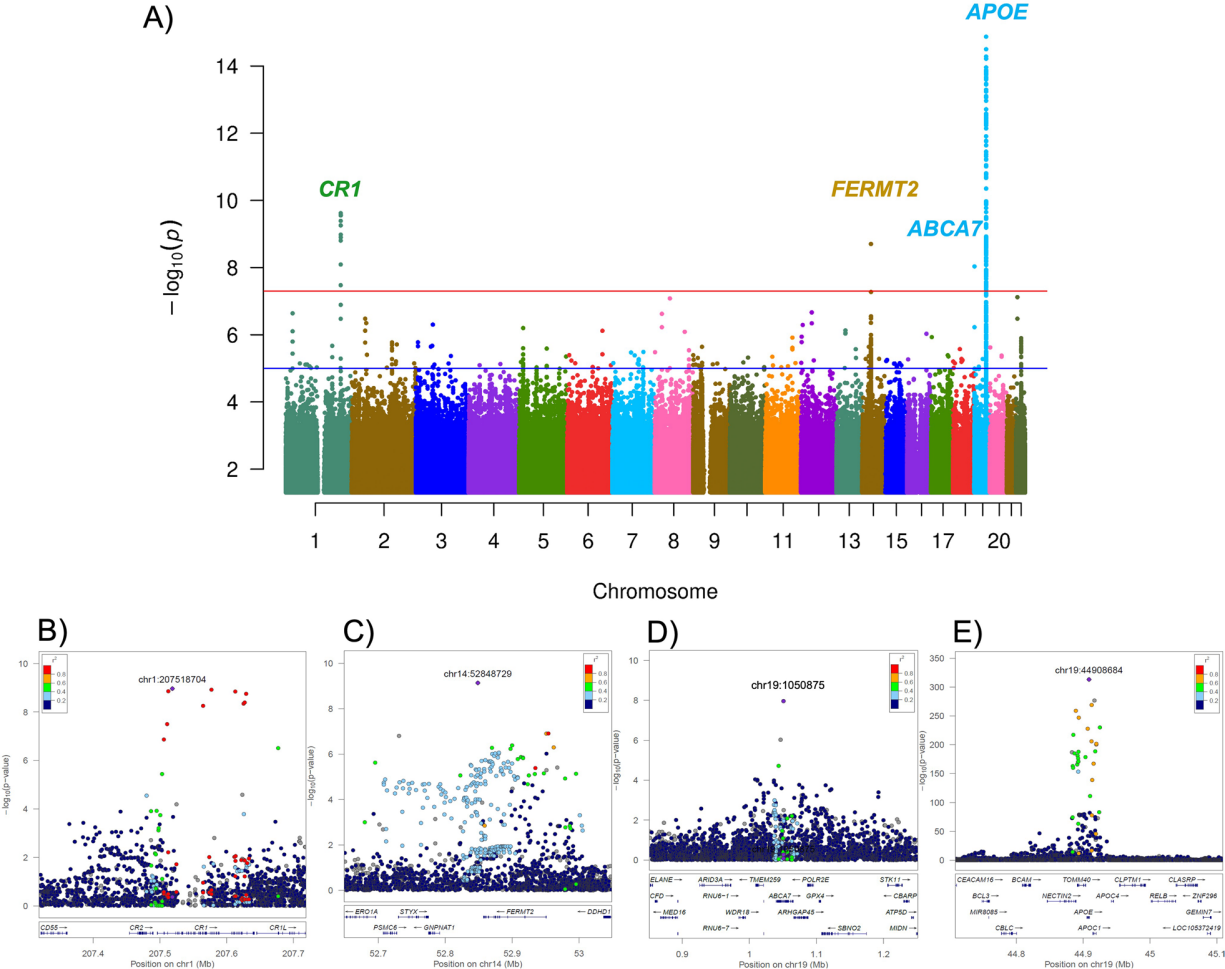
Multi-ethnic meta-analysis (N = 13,409) identified novel signals in chr 1, 14, and 19 associated with brain amyloidosis. **A** Manhattan plot showing the *p*-values in the multi-ethnic meta-analysis. The blue and red lines represent the suggestive (P = 1 × 10^−5^) and genome-wide significance thresholds (P = 5 × 10^−8^). Variants with a *p* value below 1 × 10^−15^ are not shown. Local Manhattan plot for the chr1 (**B**), chr14 (**C**), and chr19 (**D**, **E**) loci. The relative location of genes and the direction of transcription are shown in the lower portion of the locus zoom plots

The multi-ethnic meta-analysis of NHW, AFR, and ASN ethnicities across 14 different cohorts (n = 13,409) further validated the results from individual ancestry GWAS and revealed one additional locus passing the genome-wide significance threshold on chr19p.13.3 (rs12151021, P = 9.2 × 10^–09^, β = 0.07, SE = 0.01, MAF = 0.32, I^2^ = 0; Fig. 2) located near the *ABCA7* gene (Additional file 1: Fig. S9B). This same SNP is strongly associated with AD risk (P = 4.1 × 10^–30^, β = 0.1), and colocalizes (PP.H4 = 0.99) with AD risk [4]. This gene has already been shown to affect cognitive and behavioral aspects of AD [48–50]. We did not observe colocalization of this signal with eQTL for *ABCA7* mRNA levels (PP.H4 < 0.05) based on GTEx [42], however, nearby genes such as *RNU6-2* and *GRIN3B* were colocalized with rs12151021 in brain frontal cortex (PP.H4 = 0.93) and brain cortex (PP.H4 = 0.94), respectively (Additional file 1: Table S8).

### *APOE* conditional analyses

The *APOE* locus that encodes apolipoprotein E (*APOE*) is a strong genetic risk factor for AD. Previous studies have shown that *APOE* ε4 individuals have higher amyloid burden [51, 52]. Contrarily, *APOE* ε2 carriers, showing reduced Aβ deposition [53], have been associated with milder clinical and pathological AD when compared to ε4 carriers [54].

In order to determine if the signals observed in the current analysis are driven by *APOE* ε2/ε3/ε4, we performed *APOE* conditional analyses using the summary statistics data from the multi-ethnic meta-analysis (N = 13,409), using COJO [55]. In the multi-ethnic meta-analysis, rs429358, which codifies *APOE* ε4, was found to be the most significant SNP from chr19 (P = 6.2 × 10^–311^, β = 0.35, SE = 0.01, MAF = 0.19). Therefore, a conditional analysis was performed by adjusting for rs429358 (*APOE* ε4) and rs7412 (*APOE* ε2). A total of 50 SNPs on the *APOE* locus remained genome-wide significant at P < 5 × 10^–08^ (Additional file 1: Fig. S11 and Table S9), and the most significant was rs73052335 (Additional file 1: Fig. S12A). After conditioning for *APOE* ε4*, APOE* ε2, and rs73052335, we identified three additional independent signals in this region: (rs1081105/chr19:44,909,698:A:C; rs438811/chr19:44,913,484:C:T; rs4420638/chr19:44,919,689:A:G) that remained genome-wide significant in the conditional analyses, indicating that there are up to six independent signals in the *APOE* locus (Additional file 1: Table S9 and Fig. S12). We performed functional annotation and expression- and splicing-QTL mapping for these independent signals and several of these SNPs are either eQTL or sQTL for *TOMM40* and *NECTIN2*, suggesting that these genes may also be contributing to amyloid deposition independently of *APOE*. Colocalization analysis with AD risk for all the hits from *APOE* conditional analyses (N = 50) with AD risk GWAS [4] suggests more than half of these signals to be colocalizing with H4 > 0.8 (Additional file 1: Table S10).

We also performed similar analyses including only the datasets from which we have individual level data (N = 7,557), but instead of conditioning on the SNPs that codified for *APOE* ε4*, APOE* ε2, we conditioned on the full *APOE* genotypes: ε22, ε32, ε33, ε24, ε34 and ε44, codified as 0, 1, 2, 3, 4 and 5 respectively (Additional file 1: Fig. S13). One independent and additional signal (sentinel SNP: rs5117/chr19:44,915,533:T:C) passed genome-wide significance (P = 1.6 × 10^–08^, β = 0.11, SE = 0.02, MAF = 0.29), after condition for full *APOE* genotype. This SNP is in high LD with rs73052335 (D’ = 1, R^2^ = 0.23, Additional file 1: Table S11) and likely to correspond to the same signal.

### *Race-specific APOE* analyses

In order to assess whether *APOE* exhibits a race-specific effect, we analyzed the association between quantitative amyloid PET levels and *APOE*-associated variants on chr19q.13.32 (*APOE* ε4/rs429358, *APOE* ε2/rs7412, and rs5117). *APOE* ε4 SNP showed a consistent and positive association with amyloid PET levels (positive effect size in each of the analyzed races; NHW = 0.62, AFR = 0.49, and ASN = 0.14; Additional file 1: Table S12). The effect size on *APOE* ε4 in ASN was significantly lower than the NHW (two sample *t*-test, P = 3.8 × 10^–242^) and AFR (two sample *t*-test, P = 7.4 × 10^–07^) ethnicities.

We observed a protective role of *APOE* ε2 variant across all ethnicities with a consistent negative effect size (NHW = − 0.33, AFR = − 0.16, ASN = − 0.02; Additional file 1: Table S12). Although the difference in the effect size was only significant in NHW and ASN ethnicities (two sample *t*-test, P = 8.1 × 10^–18^), AFR had almost half the effect size of NHW, indicating a race-specific effect of *APOE* ε2.

The third independent signal in the *APOE* locus, rs5117, consistently positively associated with brain amyloidosis (NHW = 0.41, AFR = 0.04, and ASN = 0.11; Additional file 1: Table S12), also exhibited a race-specific effect. The observed effect size was significantly higher in NHW as compared to AFR (two sample *t*-test, P = 3.3 × 10^–09^) and ASN (two sample t-test, P = 1.9 × 10^–93^) ethnicities. The fourth independent signal in the *APOE* locus, rs73052335, also showed consistently positive association across all races (NHW = 0.56, AFR = 0.45, and ASN = 0.43), but the observed effect size was not race-specific. Overall, this data indicates that *APOE* is a complex locus in which multiple race-specific signals influence brain amyloidosis.

### *APOE* ɛ4 stratified analyses

Considering the well-established risk effect of *APOE* ε4 and protective effect of *APOE* ε2 alleles in AD, we asked if amyloid positivity also shows any *APOE* ε4-dependent difference. Therefore, we performed an *APOE* ε4-stratified analysis and observed genome-wide significant SNPs in both *APOE* ɛ4+ (N = 3,844) and *APOE* ɛ4- (N = 5,931) strata (Additional file 1: Fig. S14).

In the *APOE* ɛ4+ strata, the top hit on the *APOE* locus was driven by the *APOE* ɛ4 allele (rs429358, P = 1.5 × 10^–26^, β = 0.37, SE = 0.03, MAF = 0.19), which is capturing the association of one vs two *APOE ɛ4* alleles. The signal in the *CR1* locus, chr1q.32.2 (rs679515, P = 2.7 × 10^–08^, β = 0.15, SE = 0.03, MAF = 0.18) was in high LD (R^2^ = 0.97) with previously observed signal in the NHW and multi-ethnic meta-analysis (chr1q.32.2), also remained significant in the *APOE* ɛ4+ strata, but only nominally significant in the *APOE* ɛ4- (P = 0.02, β = 0.05, SE = 0.02). We found that this SNP also has a significant interaction with *APOE* ɛ4+ (P = 0.008) and the effect size in the *APOE* ɛ4+ vs ɛ4- is also significantly different (*p* = 0.001; Additional file 1: Table S13), indicating an interaction with *APOE*.

In case of the *APOE* ɛ4- strata, the *ABCA7* locus (chr19p.13.3) passed the genome-wide significance threshold (rs12151021, P = 3.3 × 10^–09^, β = 0.10, SE = 0.02, MAF = 0.18; Additional file 1: Fig. S14A), but was only nominal significant in the *APOE* ɛ4+ strata (P = 0.02, β = 0.05, SE = 0.02). We did not find any significant interaction between this SNP and *APOE* ɛ4, nor was the effect size in ɛ4+ vs *APOE* ɛ4- strata significantly different (Additional file 1: Table S13). Similar findings were identified for *FERMT2*, with a more significant *p*-value in *APOE* ɛ4- (P = 7.3 × 10^–06^, β = 0.16, SE = 0.03) compared to *APOE* ɛ4+ (P = 0.02, β = 0.11, SE = 0.05) but without interaction with *APOE* ɛ4.

Notably, in the *APOE* ɛ4- strata we identified rs1065853 as the most significant SNP on the *APOE* locus (P = 1.5 × 10^–13^, β =  − 0.21, SE = 0.03, MAF = 0.07). This SNP is in high LD with *APOE* ɛ2 (D’ = 0.99, R^2^ = 0.96). In addition, we identified a novel signal on chr2q.12.2 (rs567226423; P = 2.8 × 10^–08^, β = 0.81, SE = 0.15, MAF = 0.003). Functional annotation by VEP [56] suggest this SNP to be a regulatory region variant for *UXS1* protein coding gene, however, another nominally significant SNP, chr2q.12.2 (rs191708024, P = 1.9 × 10^–04^, β = 0.28, SE = 0.08, MAF = 0.01) within high LD (D’ = 1, R^2^ = 0.50), is an eQTL for *NCK2, which is* a known AD risk gene [5]. Additional studies even in larger dataset are needed to replicate this finding.

### Sex-stratified GWAS

About two thirds of people diagnosed with AD are women, however, the life expectancy for women is longer than for men [57]. Furthermore, age is the greatest risk factor for AD dementia as chances for developing AD by the age of 45 is 20% and 10% for women and men, respectively [57]. In light of these facts, we wanted to assess if amyloid burden also exhibits sex-dependent differences by performing the association analysis separately for the female (N = 5195) and male (N = 4625) strata.

**Fig. 3:**
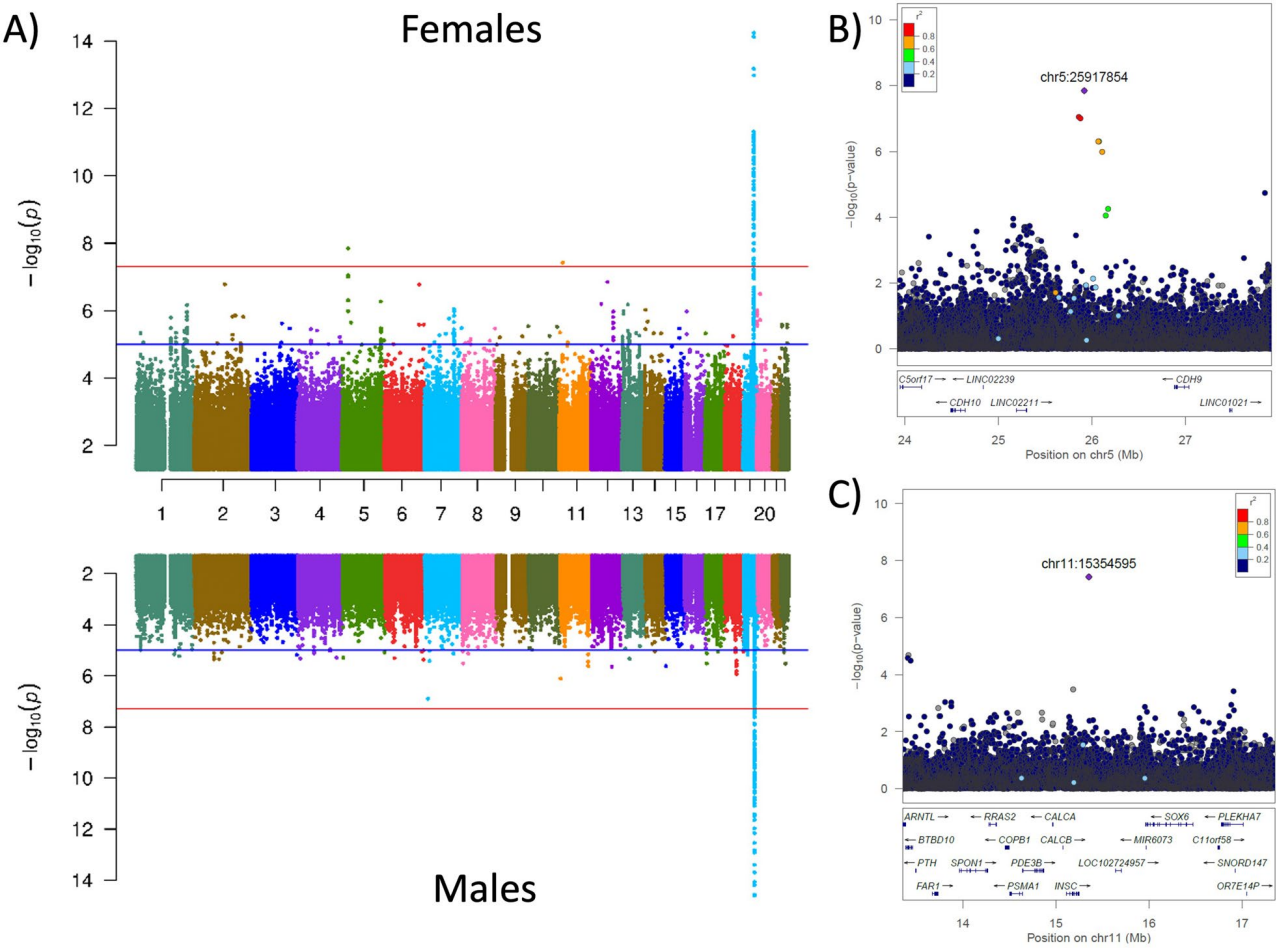
Sex stratified analyses identified several female specific signals. **A** Manhattan plot showing the *p*-values in the 5195 female and 4625 male participants across 9 cohorts. The blue and red lines represent the suggestive (P = 1 × 10^−5^) and genome-wide significance thresholds (P = 5 × 10^−8^). Variants with a p value below 1 × 10^−15^ are not shown. The observed genomic control value (λ) was 1 for both strata. Local Manhattan plot showing the genome-wide significant locus from the chr5 (B) and chr11 (C) for female-specific signals. The relative location of genes and the direction of transcription are shown in the lower portion of the locus zoom plots

No genome-wide signals were found in males, but the female-specific analyses revealed two independent genome-wide significant SNPs on chr5p.14.1 (rs529007143, β = 0.79, SE = 0.14, P = 1.4 × 10^–08^, sex-interaction P = 9.8 × 10^–07^, MAF = 0.006, I^2^ = 0; Fig. 3) and chr11p.15.2 (rs192346166, β = 0.94, SE = 0.17, P = 3.7 × 10^–08^, sex-interaction P = 1.3 × 10^–03^, MAF = 0.004, I^2^ = 0). We looked for SNPs in high LD with these signals and found several SNPs in high LD that were also highly significant, indicating them to be potential new signals and not an imputation error or artifact (Additional file 1: Table S14). In addition, in the sex-AD interaction analysis, both of the identified variants passed the suggestive significance (Additional file 1: Fig. S15) and showed a positive association with amyloid deposition for rs529007143 (β = 0.54, SE = 0.11, P = 9.8 × 10^–07^) and chr11p.15.2 (rs192346166, β = 0.41, SE = 0.13, P = 1.3 × 10^–03^) variants. Functional annotation of these rare genetic variants indicates that the intergenic upstream genes are *MSNP1* and *INSC/EHF* for rs529007143 and rs192346166, respectively (Additional file 1: Fig. S16). Expression and pQTL analyses were not informative to identify the most likely functional genes in those regions (Additional file 1: Table S15). We also did not find any overall gene-expression change between AD cases and controls for *MSNP1*. However, gene expression of *EHF* is higher in AD brains compared to controls, and more interestingly this difference is also driven by women [47]. The identified loci did not colocalized with AD risk GWAS (Additional file 1: Table S4), probably because the considered AD risk GWAS has both males and females individuals. To conclude, we have identified two novel female-specific signals that are positively associated with amyloid deposition, however, additional studies with even a larger sample size are needed to replicate these finding and proper colocalization analyses are also needed in female-only GWAS for AD risk to further validate these signals.

### Case–control stratified analyses

Population-based case–control studies have become increasingly popular for finding common polymorphisms that underlie complex human traits. In order to identify such genetic variants underlying our AD endophenotype of interest (Amyloid PET), we conducted a case–control stratified association analysis (CO = 5846, AD = 1138) and identified multiple variants from the *APOE* locus that passed the genome-wide significance in both strata (Additional file 1: Fig. S17).

Briefly, in these analyses only the *APOE* locus passed the genome-wide significance, with *APOE* ɛ4 (rs429358) being the most significant SNP. Even, the effect size in cases and controls (AD: β = 0.59, SE = 0.02, P = 1.2 × 10^–165^, MAF = 0.19; CO: β = 0.47, SE = 0.04, P = 1.9 × 10^–29^, MAF = 0.23) were in the same direction, the statistical analyses indicate that they are significantly different (*p* = 0.007, Additional file 1: Table S16). Similar findings were observed for the protective effect of *APOE* ɛ2 (AD: β = − 0.54, SE = 0.1, P = 3.7 × 10^–08^; CO; β = − 0.26, SE = 0.03, P = 2.1 × 10^–16^), with cases showing significantly higher effect size than controls (*p* = 0.007; Additional file 1: Table S16). We observed similar and non-significantly different effect sizes for *CR1*, *FERMT2*, and *ABCA7* in AD and CO (*p* > 0.29), indicating that both strata contribute to the association.

We also checked the overlap between known AD risk loci [5] and the case–control stratified GWAS. In the case-only analysis (N = 1138), we found seven SNPs to be nominally significant with consistent effect size direction except for one variant (Additional file 1: Table S17). The most significant was on chr14q.32.12/ rs7401792 (*SLC24A4*, P = 0.0010, β =  − 0.14, SE = 0.04, MAF = 0.37; Additional file 1: Table S17). In case of controls (N = 5846), we identified 11 SNPs to be nominally significant, with chr7p.21.3 (rs6943429) being the most significant (*UMAD1*, *p* = 0.0010, β = 0.05, SE = 0.02, MAF = 0.42; Additional file 1: Table S17). Overall, we found more loci to be associated with AD risk in the amyloid PET control GWAS.

### Association of known AD risk loci with amyloid burden

Next, we assessed if there is an overlap between the genetic architecture of amyloid imaging and AD risk beyond the identified SNPs in the *APOE*, *ABCA7*, *FERMT2*, and *CR1* loci that all colocalized with AD risk (PP.H4 > 0.88; Additional file 1: Table S4). Among the 82 genome-wide significant sentinel SNPs associated with AD risk reported by Bellenguez et al. [5], 18 additional AD risk variants were also nominally associated (P < 0.05) with brain amyloidosis in the current amyloid PET GWAS (Additional file 1: Table S18). This overlap of overall 21 SNPs between AD risk and amyloid PET GWAS was found to be statistically significant (hypergeometric test P = 4.1 × 10^–12^). The identified SNPs included key AD associated genes, such as *ANK3, APP, BIN1, CLU*, FERMT2, and *TREM2*. Importantly, for most of the identified SNPs, the direction of effect size (β) was consistent between Bellenguez et al. [5] and the current amyloid PET GWAS. These results suggest that amyloid imaging endophenotype can serve as a proxy for AD risk.

### Polygenic risk score analysis

In order to further determine the association of brain amyloidosis and AD risk, we determined if AD Polygenic risk score (PRS) were associated with amyloid PET. PRS were calculated with and without *APOE* region (GRCh38, chr19:43,907,927–45,908,821) from the most recent AD case–control GWAS [5]. We observed significant association between PRS for AD risk and brain amyloidosis (PRS.R2 = 0.05, P = 2.6 × 10^–85^) at P-value threshold (P_T_) equal to genome-wide significance (P_T_ = 5 × 10^–08^; Additional file 1: Fig. S18) when *APOE* was included. We also observed a significant association (PRS.R2 = 0.01, P = 1.4 × 10^–19^) even when the *APOE* region was removed from the reference GWAS. Overall, age, sex, cohort, first ten genetic principal components, and the polygenic score explained 8.7% (Full.R2 = 0.087) and 4.7% (Full.R2 = 0.047) of the variance in amyloid levels with and without *APOE*, respectively. These results further strengthen our hypothesis that there are loci besides *APOE* that are associated with amyloid burden in the brain and they also contribute to the observed association with AD risk PRS.

### Genetic covariance between amyloid PET GWAS and complex human traits

**Fig. 4:**
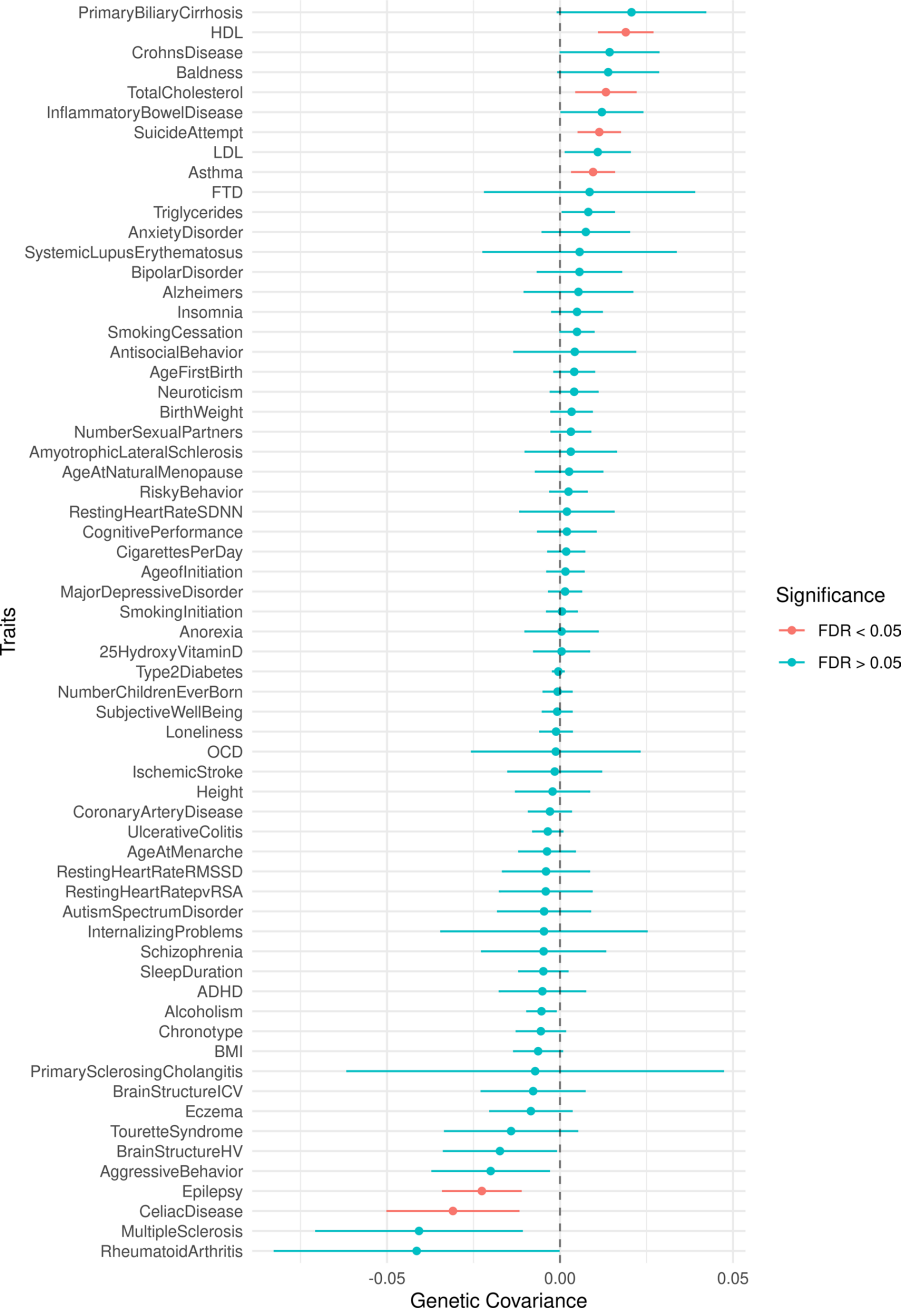
Genome-wide genetic covariance results. Genetic covariance between multi-ethnic amyloid PET GWAS (NHW = 11, 816) and 63 complex human traits. Error bars represent 95% confidence intervals

To assess whether amyloid PET endophenotype shares its genetic basis with other complex human traits, we performed genetic covariance analyses using summary statistics from amyloid PET GWAS from NHW ancestry (N = 11,556) and 63 human health-related phenotypes [58] (Additional file 1: Table S3). Pair‐wise genetic covariances between amyloid PET AD endophenotype and 63 human health-related phenotypes are shown in Fig. 4 and Additional file 1: Table S19. In total, 7 phenotypes showed significant correlation after multiple testing and sample overlap correction.

We observed a strongly positive correlation with high-density lipoprotein (HDL; cor = 0.29, SE = 0.09, FDR = 9.2 × 10^–05^), Total cholesterol (cor = 0.19, SE = 0.09, FDR = 2.5 × 10^–02^), and Asthma (cor = 0.15, SE = 0.06, FDR = 2.5 × 10^–02^), among others. Moreover, we observed significantly negative correlations with Epilepsy (cor = − 0.38, SE = 0.13, FDR = 2.5 × 10^–03^) and Celiac disease (cor = − 0.16, SE = 0.06, FDR = 2.2 × 10^–02^). Removing the *APOE* region had minimal effect on the pair-wise genetic correlations and their significance, with the exception of identifying a new significant correlation for Multiple Sclerosis (cor =  − 0.23, SE = 0.06, FDR = 7.3 × 10^–03^). Taken together, these results provide evidence of consistency in the observed polygenic trends across comparable measures from independent datasets.

## Discussion

In this study we explored the genetic basis of brain amyloidosis by analyzing the largest collection of amyloid PET imaging data to date. We used harmonized amyloid PET levels as a quantitative trait to conduct the race-specific GWAS across eight different cohorts (N = 7557). Further, the summary statistics data from 6 additional cohorts were included to perform multi-ethnic meta-analysis for identifying novel genetic associations with brain amyloidosis (N = 13,409).

The meta-analysis revealed a very strong significant genome-wide locus on the *APOE* gene region, validating previous findings [11, 14, 15]. We leveraged these large and multi-ethnic cohorts to further disentangle the role of *APOE* in brain amyloidosis and AD risk. It is known, and well accepted that there are at least two independent signals in the *APOE* gene: ɛ4 and ɛ2 [11, 15]. We found a strong and positive association of ɛ4 across populations (Effect size NHW = 0.62 [SE = 0.01], AFR = 0.49 [SE = 0.07], ASN = 0.14 [SE = 0.01]; Additional file 1: Table S12), even though the MAF and the effect size were significantly different across populations. We also observed significant heterogeneity in the effect of *APOE* ɛ2, with strong protective effect of this variant in NHW (Effect size = -0.33, SE = 0.02), but almost nothing at all in ASN (Effect size = − 0.02, SE = 0.03), and AFR showing an intermediate but not significant association of this variant with brain amyloidosis (Effect size = − 0.16, SE = 0.10). As our population principal component factor analyses indicate that our AFR population includes samples with some NHW contribution (Additional file 1: Fig. S1B), it will be important to perform future analyses evaluating local admixture mapping to determine the effect size of the *APOE* ɛ2 in individuals from a NHW or AFR background.

In addition to the known ɛ4 and ɛ2 independent variants, our *APOE* conditional analyses demonstrate that there are at least four additional risk signal in this locus on chr19q.13.32, tagged by rs73052335 (β = 0.28, SE = 0.02, P = 2.1 × 10^–71^, MAF = 0.17)/rs5117 (β = 0.07, SE = 0.01, P = 1.2 × 10^–13^, MAF = 0.29), rs1081105 (β = 0.29, SE = 0.03, P = 6.4 × 10^–24^, MAF = 0.04), rs438811 (β = − 0.15, SE = 0.01, P = 1.6 × 10^–57^, MAF = 0.41), and rs4420638 (β =  − 0.06, SE = 0.001, P = 1.0 × 10^–09^, MAF = 0.23), that have also been reported to be associated with AD risk with positive colocalization (Additional file 1: Table S10). Similar to *APOE* ɛ2, rs5117 signal shows very specific race-specific effects. This variant has a strong effect in NWH, a significant but weaker effect on ASN and no effect on AFR. Based on our analyses, it is not clear if this variant is modifying amyloid imaging through an *APOE*-dependent mechanism or by some other gene. Our eQTL analyses indicate that the additional *APOE* variants also regulate *TOMM40* or *NECTIN2* mRNA levels. *TOMM40* (translocase of outer mitochondrial membrane 40 homolog) has also been reported to be associated with LOAD and multiple LOAD-related neuroimaging phenotypes in the 19q13 region [59–61]. A poly-T polymorphism in *TOMM40* has been proposed to modify AD risk independently of *APOE* in multiple studies, but with contradictory results [59–62]. Nectin cell adhesion molecule 2 (*NECTIN2*), an important mediator of immune system, has already been shown to be downregulated in the neurons of AD cases [63] and different genetic variants in this gene have been associated with alterations in the CSF Aβ and tau levels as well as AD [2, 64, 65]. In addition, common variants in *NECTIN2*, have shown a strong association with CSF sTREM2 [66]. Besides *APOE* ɛ4 and ɛ2, various other variants have also been shown to exhibit protective or risk effect on AD. For example, a recent report suggest that the individuals having two copies of *APOE* ɛ3 Christchurch (p.R136S) mutation may slow down the progression of AD by preventing the accumulation of tau tangles and associated cell death [67]. Furthermore, multiple signals have been found in the *APOE* locus for CSF Aβ_1-42_ after controlling for *APOE* genotype and adjusting for multiple comparisons based on Bonferroni threshold [68].

The effect of *APOE* ɛ4 and ɛ2 with AD risk and AD endophenotypes is so strong that identifying, validating, and characterizing additional independent signals in this region remains challenging and additional studies are needed. In any case, these results strength the notion that multiple independent risk and protective variants are found in the *APOE* locus, and more importantly that these signals show a very clear race-specific effect. Other studies have also reported a race-specific effect of *APOE* on AD risk [69], mainly for *APOE* ɛ4. Here we demonstrate that this observation can be extended to amyloid imaging but more importantly, we demonstrate that this differential effect is more pronounced to *APOE* ɛ2 and for the new additional signal we identified. This has important implications when estimating the individual risk at a population level that may affect future clinical trial strategies and therapies. The new results from the Lecanemab study [70] indicates that this treatment has different effects depending on the ethnicity and *APOE* genotype. The results from this study could help to further understand the result from this trial.

Besides identifying multiple independent signals in the *APOE* locus, we also found two additional NHW signals in the *CR1* (chr1q.32.2, P = 9.1 × 10^–10^, β = 0.1, SE = 0.02, MAF = 0.18) and *FERMT2* (chr14q.22.1, P = 8.7 × 10^–09^, β = 0.15, SE = 0.03, MAF = 0.06) loci, and *a multi-ethnic signal in ABCA7* (chr19p.13.3, P = 9.2 × 10^–09^, β = 0.07, SE = 0.01, MAF = 0.32) locus. All these signals have already been shown to be significantly associated (*CR1* = 5.2 × 10^–33^, *FERMT2* = 5.8 × 10^–10^, *ABCA7* = 4.1 × 10^–30^) with AD risk [5] and our colocalization analyses indicate they all share a single causal variant (PP.H4 > 0.88; Additional file 1: Table S4). We observed a significant interaction between *APOE* and *CR1* signal (P = 0.008, β = 0.05, SE = 0.02; Tale S16). This signal was mainly driven by *APOE* ɛ4+ stratum (P = 3.3 × 10^–08^, β = 0.15, SE = 0.03) but also nominally significant in the *APOE* ɛ4− stratum (P = 0.05, β = 0.04, SE = 0.02), and exhibited significant difference in the effect size between both these strata (two-sample t-test *p* = 0.001). We did not observe an interaction of *APOE* with *FERMT2* and *ABCA7* signal (*p* > 0.20), also their effect sizes were not significantly different (*p* > 0.10) between ɛ4+ and ɛ4- strata.

We found a strong overlap in the genetic architecture of AD risk and brain amyloid imaging. Alzheimer disease PRS with (P = 2.6 × 10^–85^) and without (P = 1.4 × 10^–19^) *APOE* showed a strong association with amyloid imaging. Additionally, 21 of the sentinel SNPs for the latest GWAS for AD risk [5] showed at least a nominal significance (*p* < 0.05) for brain amyloidosis. For most of the overlapping SNPs, the effect size directions were consistent across both analyses. Some of the most important genes that have been associated with these SNPs included, *CR1* (Complement Receptor Type 1), *ABCA7* (ATP-Binding Cassette Sub-Family A Member 7), *BIN1* (Bridging Integrator 1), *ANK3* (Ankyrin 3), *TREM2* (Triggering Receptor Expressed On Myeloid Cells 2), and *CLU* (Clusterin). All these genes have been shown to be implicated in neuropathology and AD [40, 71–77]. For example, *ABCA7* has been shown to mediate the generation of high-density lipoprotein (HDL) with apolipoproteins [78]. Like *APOE*, *CLU* is also known as a component of lipoprotein and implicated in a wide range of biological functions including cholesterol and lipid transport [79], however, the specific roles of these genes in brain cholesterol homeostasis and its involvement in AD is still unknown. Taken together, these results indicate that genetic architecture of amyloid PET endophenotype is very similar to that of AD risk, therefore, it may be used as a proxy for explaining the yet to be discovered genetic variance in AD.

Besides the robust association of brain amyloidosis with *APOE* locus, SNPs within *RBFOX1* gene on chr16p.13.3 have also been shown to reach genome-wide significance in previous studies (top SNP rs56081887, P = 3 × 10^–09^, β = 0.61, MAF = 0.09) [15]. We did not find a significant association for this variant in our race-specific GWAS for NHW ancestry (*p* = 0.75, β = − 0.01, SE = 0.03; Additional file 1: Fig. S19) or multi-ethnic meta-analysis (*p* = 0.67, β = − 0.01, SE = 0.03). As the initial association of this SNP was identified on controls only, we analyzed if this SNP showed an association in controls-only (N = 5,846). We found rs56081887 (*p* = 0.04, β = 0.060, SE = 0.03) and rs34860942 (*p* = 0.03, β = 0.064, SE = 0.03) to be nominally associated with brain amyloidosis.

Additional interactions were found on our sex-specific analyses. Two new female-specific loci were detected in the sex-stratified multi-ethnic GWAS: *MSNP1* gene on chr5.p14.1 (rs529007143, β = 0.79, P = 1.5 × 10^–08^, MAF = 0.006) and *EHF* gene on chr11.p15.2 (rs192346166, β = 0.94, P = 3.9 × 10^–08^, MAF = 0.004). Our analyses indicate that expression level of this gene is altered in AD brains, specifically in women, supporting the race-specific association. *EHF* (ETS Homologous Factor) is a DNA-binding transcription factor activity and RNA polymerase II cis-regulatory region sequence-specific DNA binding protein that play a crucial role in regulating epithelial cell differentiation and proliferation [80], however the role of this protein on AD risk is still unclear. Future studies, focused on sex-stratified analyses in AD, will be needed to replicate these findings and identifying the functional signals on chr11 and 5 to better understand the role of *EHF* on AD risk in females.

Another novel signal identified in this study is the AFR-specific signal on chr8q.22.1 (rs2271774, β = 0.98, P = 7.8 × 10^–09^, MAF = 0.028) that, based on our functional mapping, is driven by *PTDSS1*. This gene encodes the enzyme phosphatidylserine synthase 1 (PSS1), which is involved in the transport of phospholipids between endoplasmic reticulum and mitochondria [81], and which is highly expressed in brain tissue, especially in neurons. This same SNP has a MAF of 1.3% in ASN (N = 336, *p* = 0.84, β = − 0.04, SE = 0.19, MAF = 0.013) and 0.21% in NHW (N = 6329, *p* = 0.84, β = 0.13, SE = 0.56, MAF = 0.0021) but it was not even nominally associated in these two ancestries, suggesting it to be an AFR-specific signal or be a false-positive signal. Additional studies with an even larger AFR-specific sample size will be needed to further confirm this finding.

Besides the positive genetic overlap of amyloid PET AD endophenotype with different neurological disorders such as AD, Amyotrophic Lateral Sclerosis (ALS), and Frontotemporal dementia (FTD), we also found a significant genetic correlation with a number of different human traits. We observed strong genetic correlations with HDL and total cholesterol levels (Fig. 4 and Additional file 1: Table S19). It is also interesting that we observed significant negative correlations between Aβ deposition and brain structure hippocampus and intracranial volumes, validating existing findings that suggest brain volume loss during the mild cognitive impairment (MCI) to AD transition [82]. Notably, we observed strong positive genetic correlation between our trait of interest and different inflammatory disorders such as Crohn’s disease (cor = 0.07, SE = 0.04, FDR < 0.19), ALS (cor = 0.03, SE = 0.06, FDR < 0.8), and Primary Biliary Cirrhosis (cor = 0.08, SE = 0.05, FDR < 0.21). Moreover, some genetic traits that were significantly negatively correlated were also related to the dysregulation of immune response e.g. Celiac disease (cor = − 0.16, SE = 0.06, FDR < 2.2 × 10^–02^) and Multiple Sclerosis (cor = − 0.19, SE = 0.07, FDR < 0.06). These results highlight the shared genetic architecture underlying central mechanism in AD and other neuroinflammatory disorders [83, 84]. Recent GWAS and pathway analyses have emphasized the crucial role of the innate immune system and neuroinflammation in the pathogenesis of AD [85, 86]. To that end, targeting neuroinflammation by modulating different phagocytic receptors e.g. *CD33* inhibition [87] and/or *TREM2* activation [88, 89] have been suggested as valuable therapeutic strategies to enhance neuroprotective microglia and reduce neuroinflammation, which is crucial for preventing and treating AD [85].

Similar to other GWAS, the present study also bears some important limitations. Although we have compiled and leveraged the largest collection of amyloid PET datasets reported-to-date, the sample size for all populations were not comparable. Significantly reduced sample size from the AFR and ASN ancestries might be the most profound hurdle in achieving genome-wide significance for loci with small effect sizes across multi-ethnic meta-analysis. Nevertheless, we showed that some of our suggestive and nominally significant loci have the same direction of allelic effects for established AD risk associated variants, suggesting that we might have achieved genome-wide significance with a relatively larger sample size. One potential reason for relatively lower sample size is the difficulty in obtaining the amyloid PET data through scanning due to limited availability of scanning platform in the under-developed countries and their lesser representation in developed countries where such facilities are readily available. Another significant constraint was the cis-eQTL dataset that was obtained from GTEx database which only represents cognitively normal individuals and not those diagnosed with AD. As more amyloid PET imaging data are obtained by different centers with larger population sizes, future studies leveraging these larger samples sizes will have better potential for validating existing findings and identifying additional genes associated with brain amyloidosis.

## Conclusion

We have performed the largest reported to-date amyloid PET GWAS (N = 13,409) that has confirmed the previously known association of the *APOE* locus with brain amyloidosis. In addition to recapitulating the established associations, we have identified novel variants in the *ABCA7, CR1,* and *FERMT2* regions as well as sex-specific variants that affect amyloid deposition. We have employed a combination of genetic and functional analytic approaches for identifying putative candidate genes that warrant follow-up genetic and functional studies to confirm their role in brain amyloidosis. This study highlights the importance and need of large-scale genetic studies focusing on brain amyloidosis in diverse populations for finding universal candidate therapeutic targets for AD.

## Supplementary Information

**Additional file 1.** Supplementary tables and figures

## Data Availability

All data generated in this study are included in this published article.

## Abbreviations

AD: Alzheimer’s disease
Aβ: Amyloid beta
GWAS: Genome-wide association studies
PiB: Pittsburgh compound-B
MAP: Memory and aging project
Knight-ADRC: Knight alzheimer’s disease research center
ADNI: Alzheimer's disease neuroimaging initiative
DIAN: Dominantly inherited alzheimer network
A4: Anti-amyloid treatment in asymptomatic alzheimer's disease
ADNI-DOD: adni department of defense
AIBL: Australian imaging, biomarkers and lifestyle
HABS: Harvard aging brain study
UPitt: University of pittsburgh
MCSA: Mayo clinic study of aging
WRAP: Wisconsin registry for alzheimer's prevention
BACS: Berkeley aging cohort study
CDR: Clinical dementia rating
PCs: Principal components
SUVR: Standardize uptake value ratios
SD: Standard deviation
MAF: Minor allele frequency
QC: Quality control
IBD: Identity-by-descent
PCA: Principal component analysis
NHW: Non-Hispanic Whites
AFR: American Africans
ASN: Asians
StdErr: Standard error
GNOVA: Genetic covariance analyzer
GTEx: Genotype-tissue expression
PRS: Polygenic risk score
LD: Linkage disequilibrium
QTL: Quantitative trait loci
sQTLs: Single-tissue splicing QTLs
*APOE*: Apolipoprotein E
CAA: Cerebral amyloid angiopathy
CO: Controls
CR1: Complement receptor 1
HDL: High-density lipoprotein
TOMM40: Translocase of outer mitochondrial membrane 40 homolog
EHF: ETS homologous factor
ABCA7: ATP-binding cassette sub-family a member 7
BIN1: Bridging integrator 1
ANK3: Ankyrin 3
TREM2: Triggering receptor expressed on myeloid cells 2
CLU: Clusterin
ALS: Amyotrophic lateral sclerosis
MCI: Mild cognitive impairment
