# Supplementary material for: Large multi-ethnic genetic analyses of amyloid imaging identify new genes for Alzheimer disease
Source: Acta Neuropathol Commun. 2023 Apr 26;11:68. doi: 10.1186/s40478-023-01563-4 (PMC10134547; doi:10.1186/s40478-023-01563-4)
Supplement: Supplementary file 1 — Additional file 1. Supplementary tables and figures [file 40478_2023_1563_MOESM1_ESM.docx]

**Supplementary Figures and Tables**

| **Ethnicity** | **N** | **Female (%)** | **Male (%)** | **Age (mean)** | **Age (SD)** | ***APOE* ε4+**  **(%)** | **Cases (%)** | **Controls (%)** |
| --- | --- | --- | --- | --- | --- | --- | --- | --- |
| **NHW** | 7,036 | 53.67 | 46.33 | 71 | 9 | 36.3 | 15.05 | 77.63 |
| **AFR** | 359 | 65.74 | 34.26 | 70 | 7.55 | 39.55 | 15.32 | 75.49 |
| **ASN** | 162 | 51.85 | 48.15 | 70 | 14.34 | 29.01 | 14.81 | 69.75 |
| **Table S1a: Demographics of amyloid PET GWAS participants from 8 cohorts based on their ethnicity.** This table summarizes basic demographic information of participants that have both phenotype and genotype data available. In this study, participants belonging to three different ethnicities, e.g. non-Hispanic White (NHW), American African (AFR), and Asian (ASN) were analyzed. For each ethnicity, we report number of participants, percentage of females and males, mean age of the participants and standard deviation (SD) in the age, percentage of *APOE* ε4-carriers (*APOE* ε4+ participants), and percentage of cases and control participants, where available. | | | | | | | | |

| **Ethnicity** | **N** | **Female (%)** | **Male (%)** | **Age (mean)** | **Age (SD)** | ***APOE* ε4+ (%)** | **Cases (%)** | **Controls (%)** |
| --- | --- | --- | --- | --- | --- | --- | --- | --- |
| **NHW** | 11,556 | 50.09 | 49.91 | 68.93 | 8.29 | 30.98 | 26.20 | 70.46 |
| **AFR** | 359 | 65.74 | 34.26 | 70 | 7.55 | 39.55 | 15.32 | 75.49 |
| **ASN** | 1,494 | 54.24 | 45.62 | 70.08 | 11.53 | 35.36 | 27.91 | 64.38 |
| **Table S1b: Demographics of amyloid PET GWAS participants from 15 cohorts based on their ethnicity.** This table summarizes basic demographic information of participants included in the analysis based on their ethnicity. In this study, participants belonging to three different ethnicities, e.g. non-Hispanic White (NHW), American African (AFR), and Asian (ASN) were analyzed. For each ethnicity, we report number of participants, percentage of females and males, mean age of the participants and standard deviation (SD) in the age, percentage of *APOE* ε4-carriers (*APOE* ε4+ participants), and percentage of cases and control participants, where available. | | | | | | | | |

| **GWAS** | **Correlation coefficient (R^2^)** | **P-value** |
| --- | --- | --- |
| Race-specific multi-ethnic | 0.98 | 2.2×10^-16^ |
| *APOE* ɛ4+ | 0.88 | 2.2×10^-16^ |
| *APOE* ɛ4- | 0.93 | 2.2×10^-16^ |
| Female-specific | 0.96 | 2.2×10^-16^ |
| Male-specific | 0.97 | 2.2×10^-16^ |
| **Table S2: Correlation of P-value between Joint and Meta-analysis GWAS.** R^2^ represents the correlation coefficient and P-value represents the strength of significance when the P-values from the joint- and meta-analysis GWAS are assessed for correlation using “cor.test” function in R. | | |

| **Trait** | **Sample Size** | **Reference** |
| --- | --- | --- |
| 25_Hydroxyvitamin D | 78,352 | https://www.ncbi.nlm.nih.gov/pubmed/29343764 |
| ADHD | 53,293 | https://www.ncbi.nlm.nih.gov/pubmed/30478444 |
| Age at First Birth | 241,781 | https://www.ncbi.nlm.nih.gov/pubmed/27798627 |
| Age at Menarche | 252,514 | https://www.ncbi.nlm.nih.gov/pubmed/28436984 |
| Age at Natural Menopause | 69,360 | http://www.ncbi.nlm.nih.gov/pubmed/26414677 |
| Age of Initiation - Smoking | 262,990 | https://www.ncbi.nlm.nih.gov/pubmed/30643251 |
| Aggressive Behavior | 18,988 | http://www.ncbi.nlm.nih.gov/pubmed/26087016 |
| Alcoholism | 537,349 | https://www.ncbi.nlm.nih.gov/pubmed/30643251 |
| Alzheimer Disease | 63,926 | https://www.ncbi.nlm.nih.gov/pubmed/30820047 |
| Amyotrophic Leteral Sclerosis | 36,052 | http://www.ncbi.nlm.nih.gov/pubmed/27455348 |
| Anorexia Nervosa | 72,517 | https://www.ncbi.nlm.nih.gov/pubmed/31308545 |
| Antisocial Behavior | 16,400 | https://www.ncbi.nlm.nih.gov/pubmed/28979981 |
| Anxiety Disorder | 31,890 | https://www.ncbi.nlm.nih.gov/pubmed/31116379 |
| Asthma | 142,486 | https://www.ncbi.nlm.nih.gov/pubmed/29273806 |
| Autism Spectrum Disorder | 46,351 | https://www.ncbi.nlm.nih.gov/pubmed/30804558 |
| Baldness | 52,874 | https://www.ncbi.nlm.nih.gov/pubmed/28196072 |
| Bipolar Disorder | 51,710 | https://www.ncbi.nlm.nih.gov/pubmed/31043756 |
| Birth Weight | 298,142 | https://www.ncbi.nlm.nih.gov/pubmed/31043758 |
| BMI | 795,640 | https://www.ncbi.nlm.nih.gov/pubmed/30124842 |
| Bone Mineral Density Neck | 32,961 | http://www.ncbi.nlm.nih.gov/pubmed/22504420 |
| Bone Mineral Density Spine | 31,800 | http://www.ncbi.nlm.nih.gov/pubmed/22504420 |
| Brain Structure ICV | 26,577 | https://www.ncbi.nlm.nih.gov/pubmed/27694991 |
| Brain Structure HV | 26,814 | https://www.ncbi.nlm.nih.gov/pubmed/28098162 |
| Celiac Disease | 15,283 | http://www.ncbi.nlm.nih.gov/pubmed/20190752 |
| Chronotype | 127,898 | http://www.ncbi.nlm.nih.gov/pubmed/27494321 |
| Cigarettes per day | 263,954 | https://www.ncbi.nlm.nih.gov/pubmed/30643251 |
| Cognitive Performance | 257,828 | https://www.ncbi.nlm.nih.gov/pubmed/30038396 |
| Coronary Artery Disease | 184,305 | http://www.ncbi.nlm.nih.gov/pubmed/26343387 |
| Crohn's Disease | 40,266 | https://www.ncbi.nlm.nih.gov/pubmed/28067908 |
| Eczema | 40,835 | http://www.ncbi.nlm.nih.gov/pubmed/26482879 |
| Epilepsy | 34,853 | http://www.ncbi.nlm.nih.gov/pubmed/25087078 |
| Frontotemporal Dementia | 6,462 | https://www.ncbi.nlm.nih.gov/pubmed/24943344 |
| HDL Cholesterol | 99,900 | http://www.ncbi.nlm.nih.gov/pubmed/20686565 |
| Height | 709,706 | https://www.ncbi.nlm.nih.gov/pubmed/30124842 |
| Inflammatory Bowel Disease | 59,957 | https://www.ncbi.nlm.nih.gov/pubmed/28067908 |
| Insomnia | 113,006 | https://www.ncbi.nlm.nih.gov/pubmed/28604731 |
| Internalizing Problems | 4,596 | http://www.ncbi.nlm.nih.gov/pubmed/24839885 |
| Ischaemic Stroke | 29,633 | https://www.ncbi.nlm.nih.gov/pubmed/26935894 |
| LDL Cholesterol | 95,454 | http://www.ncbi.nlm.nih.gov/pubmed/20686565 |
| Loneliness | 445,024 | https://www.ncbi.nlm.nih.gov/pubmed/29970889 |
| Major Depressive Disorder | 500,199 | https://www.ncbi.nlm.nih.gov/pubmed/30718901 |
| Multiple Sclerosis | 27,148 | http://www.ncbi.nlm.nih.gov/pubmed/21833088 |
| Neuroticism | 170,911 | http://www.ncbi.nlm.nih.gov/pubmed/27089181 |
| Number of Children Ever Born | 318,463 | https://www.ncbi.nlm.nih.gov/pubmed/27798627 |
| Number of Sexual Partners | 370,711 | https://www.ncbi.nlm.nih.gov/pubmed/30643258 |
| Obsessive Compulsive Disorder | 9,725 | https://www.ncbi.nlm.nih.gov/pubmed/28761083 |
| Primary Biliary Cirrhosis | 13,239 | http://www.ncbi.nlm.nih.gov/pubmed/26394269 |
| Primary Sclerosing Cholangitis | 14,890 | https://www.ncbi.nlm.nih.gov/pubmed/27992413 |
| Resting Heart Rate SDNN | 28,122 | https://www.ncbi.nlm.nih.gov/pubmed/28613276 |
| Resting Heart Rate RMSSD | 26,785 | https://www.ncbi.nlm.nih.gov/pubmed/28613276 |
| Resting Heart Rate pvRSA/HF | 24,342 | https://www.ncbi.nlm.nih.gov/pubmed/28613276 |
| Rheumatoid Arthritis | 58,284 | http://www.ncbi.nlm.nih.gov/pubmed/24390342 |
| Risky Behavior | 466,571 | https://www.ncbi.nlm.nih.gov/pubmed/30643258 |
| Schizophrenia | 105,318 | https://www.ncbi.nlm.nih.gov/pubmed/29483656 |
| Sleep Duration | 127,573 | http://www.ncbi.nlm.nih.gov/pubmed/27494321 |
| Smoking Cessation | 312,821 | https://www.ncbi.nlm.nih.gov/pubmed/30643251 |
| Smoking Initiation | 632,802 | https://www.ncbi.nlm.nih.gov/pubmed/30643251 |
| Subjective Well-being | 298,420 | http://www.ncbi.nlm.nih.gov/pubmed/27089181 |
| Suicide Attempt | 50,264 | https://www.ncbi.nlm.nih.gov/pubmed/30116032 |
| Systemic Lupus Erythematosus | 14,267 | http://www.ncbi.nlm.nih.gov/pubmed/26502338 |
| Total Cholesterol | 100,184 | http://www.ncbi.nlm.nih.gov/pubmed/20686565 |
| Tourette Syndrome | 14,307 | https://www.ncbi.nlm.nih.gov/pubmed/30818990 |
| Triglycerides | 96,598 | http://www.ncbi.nlm.nih.gov/pubmed/20686565 |
| Type 2 Diabetes | 159,208 | https://www.ncbi.nlm.nih.gov/pubmed/28566273 |
| Ulcerative Colitis | 45,975 | https://www.ncbi.nlm.nih.gov/pubmed/28067908 |
| **Table S3: Sources for Summary Statistics Used in Genetic Correlation Analyses.** This table summarizes the list of 63 complex human traits consider for checking genetic correlation and covariance with amyloid PET endophenotype. | | |

| **SNP (Gene)** | **PP.H0** | **PP.H1** | **PP.H2** | **PP.H3** | **PP.H4** | **Analyses** |
| --- | --- | --- | --- | --- | --- | --- |
| rs12151021  (*ABCA7*) | 2.4×10^-09^ | 2.6×10^-07^ | 1.0×10^-05^ | 4.7×10^-04^ | 0.99 | Meta |
| rs117834516  (*FERMT2*) | 9.2×10^-06^ | 1.4×10^-02^ | 7.1×10^-05^ | 0.11 | 0.88 | NHW, Meta |
| rs6656401  (*CR1*) | 1.8×10^-15^ | 1.2×10^-12^ | 8.2×10^-06^ | 4.7×10^-03^ | 0.99 | NHW, Meta |
| rs429358  (*APOE* ɛ4) | 0.00 | 0.00 | 8.7×10^-311^ | 0.00 | 1 | NHW, Meta, NHW, ASN, AFR |
| rs7412  (*APOE* ɛ2) | 0.00 | 0.00 | 8.8×10^-311^ | 0.00 | 1 | NHW, Meta, NHW, ASN, AFR |
| rs2271774  (*PTDSS1*) | 0.05 | 0.81 | 0.004 | 0.08 | 0.06 | AFR |
| rs567226423  (*UXS1*) | 0.66 | 0.12 | 0.17 | 0.03 | 0.01 | *APOE* ɛ4- |
| rs529007143  (*MSNP1*) | 0.75 | 0.10 | 0.13 | 0.02 | 0.005 | Female-Specific |
| rs192346166  (*INSC*) | 0.57 | 0.05 | 0.35 | 0.03 | 0.006 | Female-Specific |
| rs5117 | 0.00 | 0.00 | 8.8×10^-311^ | 0.00 | 1 | APOE-conditional |
| rs73052335 | 0.00 | 0.00 | 8.9×10^-311^ | 0.00 | 1 | APOE-conditional |
| **Table S4: Colocalization of the genome-wide signals for amyloid imaging with AD Risk.** We selected ± 500 kb region for each SNP to check the colocalization with AD risk. PP represent the posterior probability of: neither trait has a genetic association in the region (H0), only trait 1 has a genetic association in the region (H1), only trait 2 has a genetic association in the region (H2), both traits are associated, but with different causal variants (H3), and both traits are associated and share a single causal variant (H4). | | | | | | |

| **# SNPs** | **PP.H0.abf** | **PP.H1.abf** | **PP.H2.abf** | **PP.H3.abf** | **PP.H4.abf** | **Tissue** | **Gene** |
| --- | --- | --- | --- | --- | --- | --- | --- |
| 75 | 0.00 | 0.00 | 0.00 | 0.01 | 0.99 | Brain_Caudate_basal_ganglia | *CR1* |
| 36 | 0.00 | 0.00 | 0.00 | 0.02 | 0.98 | Brain_Cortex | *CR1* |
| 21 | 0.00 | 0.01 | 0.00 | 0.02 | 0.97 | Brain_Putamen_basal_ganglia | *CR1* |
| 21 | 0.00 | 0.03 | 0.00 | 0.01 | 0.96 | Brain_Nucleus_accumbens_basal_ganglia | *CR1* |
| 16 | 0.00 | 0.08 | 0.00 | 0.02 | 0.90 | Brain_Frontal_Cortex_BA9 | *CR1* |
| **Table S5: Colocalization of multi-ethnic meta-analysis hit from chr1q.32.2 (rs6656401) on *CR1* locus with GTEx data.** Total SNPs represent the number of SNP in the considered region that are tested for colocalization with our SNP of interest (rs6656401/ chr1:207518704:A:G). PP represent the posterior probability of: neither trait has a genetic association in the region (H0), only trait 1 has a genetic association in the region (H1), only trait 2 has a genetic association in the region (H2), both traits are associated, but with different causal variants (H3), and both traits are associated and share a single causal variant (H4). | | | | | | | |

| **# SNPs** | **PP.H0.abf** | **PP.H1.abf** | **PP.H2.abf** | **PP.H3.abf** | **PP.H4.abf** | **Tissue** | **Gene** |
| --- | --- | --- | --- | --- | --- | --- | --- |
| 16 | 0.00 | 0.04 | 0.00 | 0.03 | 0.93 | Whole_Blood | *STYX* |
| 30 | 0.09 | 0.20 | 0.01 | 0.02 | 0.68 | Brain_Substantia_nigra | *STYX* |
| 46 | 0.00 | 0.00 | 0.74 | 0.18 | 0.08 | Whole_Blood | *FERMT2* |
| 43 | 0.06 | 0.00 | 0.89 | 0.00 | 0.05 | Brain_Cerebellum | *STYX* |
| 40 | 0.01 | 0.00 | 0.93 | 0.00 | 0.05 | Brain_Hippocampus | *STYX* |
| **Table S6: Colocalization of multi-ethnic meta-analysis hit from chr14q.22.1 (rs117834516) on *FERMT2* locus with GTEx data.** Total SNPs represent the number of SNP in the considered region that are tested for colocalization with our SNP of interest (rs117834516/ chr14:52848729:C:T). PP represent the posterior probability of: neither trait has a genetic association in the region (H0), only trait 1 has a genetic association in the region (H1), only trait 2 has a genetic association in the region (H2), both traits are associated, but with different causal variants (H3), and both traits are associated and share a single causal variant (H4). | | | | | | | |

| **# SNPs** | **PP.H0.abf** | **PP.H1.abf** | **PP.H2.abf** | **PP.H3.abf** | **PP.H4.abf** | **Tissue** | **Gene** |
| --- | --- | --- | --- | --- | --- | --- | --- |
| 97 | 2.8×10^-08^ | 2.8×10^-09^ | 0.921 | 0.009 | 0.069 | Whole_Blood | *CPQ* |
| 25 | 0.354 | 0.0005 | 0.602 | 0.0008 | 0.041 | Brain_Hypothalamus | *MTERF3* |
| 37 | 0.023 | 3.2×10^-05^ | 0.935 | 0.001 | 0.040 | Whole_Blood | *TSPYL5* |
| 54 | 0.009 | 1.8×10^-05^ | 0.960 | 0.002 | 0.028 | Brain_Cerebellar_Hemisphere | *TSPYL5* |
| 51 | 3.9×10^-06^ | 6.9×10^-09^ | 0.970 | 0.002 | 0.027 | Brain_Cerebellum | *TSPYL5* |
| 123 | 0.003 | 1.2×10^-05^ | 0.973 | 0.003 | 0.019 | Brain_Cerebellum | *PTDSS1* |
| 36 | 0.519 | 0.0006 | 0.461 | 0.0005 | 0.017 | Brain_Cortex | *TSPYL5* |
| 3 | 0.862 | 0.0001 | 0.127 | 1.8×10^-05^ | 0.009 | Whole_Blood | *SDC2* |
| 8 | 0.606 | 0.0001 | 0.384 | 7.5×10^-05^ | 0.008 | Brain_Caudate_basal_ganglia | *ENSG00000260640* |
| 10 | 0.798 | 0.0005 | 0.195 | 0.0001 | 0.004 | Brain_Caudate_basal_ganglia | *PTDSS1* |
| 3 | 0.927 | 0.0001 | 0.068 | 9.3×10^-06^ | 0.003 | Whole_Blood | *PTDSS1* |
| 6 | 0.942 | 0.0002 | 0.055 | 9.8×10^-06^ | 0.002 | Brain_Nucleus_accumbens_basal_ganglia | *CPQ* |
| 1 | 0.933 | 3.1×10^-05^ | 0.063 | 0 | 0.002 | Brain_Hippocampus | *CFAP418-AS1* |
| 2 | 0.950 | 6.3×10^-05^ | 0.047 | 1.5×10^-06^ | 0.001 | Brain_Putamen_basal_ganglia | *TSPYL5* |
| 2 | 0.963 | 0.0001 | 0.034 | 2.9×10^-06^ | 0.001 | Brain_Anterior_cingulate_cortex_BA24 | *PTDSS1* |
| 17 | 0.973 | 0.001 | 0.023 | 2.6×10^-05^ | 0.001 | Brain_Putamen_basal_ganglia | *CPQ* |
| 3 | 0.946 | 4.9×10^-05^ | 0.052 | 1.8×10^-06^ | 0.0009 | Brain_Amygdala | *TSPYL5* |
| 2 | 0.986 | 5.4×10^-05^ | 0.013 | 3.7×10^-07^ | 0.0003 | Brain_Hypothalamus | *CPQ* |
| 2 | 0.996 | 5.4×10^-05^ | 0.003 | 9.1×10^-08^ | 9.1×10^-05^ | Brain_Frontal_Cortex_BA9 | *CPQ* |
| **Table S7: Colocalization of AFR-specific hit from chr8q.22.1 (rs2271774) with GTEx data.** Total SNPs represent the number of SNP in the considered region that are tested for colocalization with our SNP of interest (rs2271774/chr8:96306362:A:G). PP represent the posterior probability of: neither trait has a genetic association in the region (H0), only trait 1 has a genetic association in the region (H1), only trait 2 has a genetic association in the region (H2), both traits are associated, but with different causal variants (H3), and both traits are associated and share a single causal variant (H4). | | | | | | | |

| **# SNPs** | **PP.H0.abf** | **PP.H1.abf** | **PP.H2.abf** | **PP.H3.abf** | **PP.H4.abf** | **Tissue** | **Gene** |
| --- | --- | --- | --- | --- | --- | --- | --- |
| 35 | 0.00 | 0.00 | 0.05 | 0.01 | 0.94 | Brain_Cortex | *GRIN3B* |
| 25 | 0.00 | 0.00 | 0.05 | 0.01 | 0.94 | Brain_Putamen_basal_ganglia | *RNU6-2* |
| 29 | 0.00 | 0.00 | 0.06 | 0.01 | 0.93 | Brain_Frontal_Cortex_BA9 | *RNU6-2* |
| 31 | 0.00 | 0.00 | 0.93 | 0.03 | 0.04 | Brain_Cerebellar_Hemisphere | *ABCA7* |
| 41 | 0.00 | 0.00 | 0.92 | 0.05 | 0.04 | Brain_Cerebellum | *ABCA7* |
| 14 | 0.07 | 0.00 | 0.89 | 0.00 | 0.03 | Brain_Spinal_cord_cervical_c-1 | *ABCA7* |
| 10 | 0.00 | 0.00 | 0.96 | 0.00 | 0.03 | Brain_Putamen_basal_ganglia | *ABCA7* |
| **Table S8: Colocalization of multi-ethnic meta-analysis hit from chr19p.13.3 (rs12151021) on *ABCA7* locus with GTEx data.** Total SNPs represent the number of SNP in the considered region that are tested for colocalization with our SNP of interest (rs12151021/ chr19:1050875:A:G). PP represent the posterior probability of: neither trait has a genetic association in the region (H0), only trait 1 has a genetic association in the region (H1), only trait 2 has a genetic association in the region (H2), both traits are associated, but with different causal variants (H3), and both traits are associated and share a single causal variant (H4). | | | | | | | |

| **Multi-ethnic Meta-analysis** | | | | | | | **APOE Conditional Analyses** | | | | | **Ensembl Variant Effect Predictor (VEP)** | | | | **GTEx** | | |
| --- | --- | --- | --- | --- | --- | --- | --- | --- | --- | --- | --- | --- | --- | --- | --- | --- | --- | --- |
| **Chr** | **SNP (rsID)** | **refA** | **freq** | **b** | **se** | **p** | **bC** | **bC_se** | **pC** | **Group** | **Consequence** | | **Biotype** | **Symbol** | **eQTL** | | **sQTL** |  |
| 19 | chr19:44916825:A:C (rs73052335) | C | 0.17 | 0.54 | 0.02 | 6.9×10^-279^ | 0.28 | 0.02 | 2.1×10^-71^ | 1 | intron_variant | | protein_coding | *APOC1* |  | |  |  |
| 19 | chr19:44912678:G:T (rs7256200) | T | 0.18 | 0.51 | 0.01 | 1.1×10^-270^ | 0.26 | 0.02 | 2.2×10^-66^ | 1 | upstream_gene_variant | | protein_coding | *APOC1* |  | |  |  |
| 19 | chr19:44924977:G:A (rs66626994) | A | 0.19 | 0.46 | 0.01 | 2.4×10^-230^ | 0.22 | 0.01 | 1.8×10^-52^ | 1 | upstream_gene_variant | | lncRNA | *APOC1P1* |  | | *TOMM40* |  |
| 19 | chr19:44883210:G:GTAA (rs142042446) | GTAA | 0.17 | 0.49 | 0.02 | 2.5×10^-187^ | 0.25 | 0.02 | 5×10^-47^ | 1 |  | |  |  |  | | *TOMM40* |  |
| 19 | chr19:44892587:G:A (rs34095326) | A | 0.13 | 0.44 | 0.02 | 5.6×10^-154^ | 0.22 | 0.02 | 3.3×10^-37^ | 1 | downstream_gene_variant | | protein_coding | *NECTIN2* | *BCAM* | | *TOMM40* |  |
| 19 | chr19:44901805:A:G (rs1038026) | G | 0.42 | -0.21 | 0.01 | 1.8×10^-76^ | -0.11 | 0.01 | 1.1×10^-21^ | 1 | upstream_gene_variant | | protein_coding | *APOE* |  | |  |  |
| 19 | chr19:44901715:T:C (rs1038025) | C | 0.42 | -0.21 | 0.01 | 4.6×10^-76^ | -0.11 | 0.01 | 2×10^-21^ | 1 | upstream_gene_variant | | protein_coding | *APOE* |  | |  |  |
| 19 | chr19:44904531:G:A (rs7259620) | A | 0.41 | -0.21 | 0.01 | 1.2×10^-72^ | -0.11 | 0.01 | 4.5×10^-20^ | 1 | upstream_gene_variant | | protein_coding | *APOE* |  | |  |  |
| 19 | chr19:44917843:G:A (rs3925681) | A | 0.38 | -0.18 | 0.01 | 1.7×10^-52^ | -0.1 | 0.01 | 1×10^-17^ | 1 | intron_variant | | protein_coding | *APOC1* |  | | *TOMM40* |  |
| 19 | chr19:44903281:C:CG (rs34215622) | CG | 0.4 | -0.21 | 0.01 | 2×10^-58^ | -0.11 | 0.01 | 1.1×10^-17^ | 1 |  | |  |  |  | |  |  |
| 19 | chr19:44899220:C:T (rs34878901) | T | 0.38 | -0.18 | 0.01 | 9.6×10^-52^ | -0.1 | 0.01 | 1.5×10^-16^ | 1 | intron_variant | | protein_coding | *TOMM40* |  | |  |  |
| 19 | chr19:44888997:C:T (rs6857) | T | 0.21 | 0.33 | 0.01 | 4.8×10^-259^ | 0.07 | 0.01 | 6.3×10^-14^ | 1 | 3_prime_UTR_variant | | protein_coding | *NECTIN2* |  | | *TOMM40* |  |
| 19 | chr19:44915704:T:C (rs3826688) | T | 0.34 | -0.18 | 0.01 | 5.9×10^-44^ | -0.1 | 0.01 | 8.8×10^-14^ | 1 |  | |  |  | *APOE, APOC1* | | *APOC1, APOE, APOC1P1* |  |
| 19 | chr19:44915533:T:C (rs5117) | C | 0.29 | 0.25 | 0.01 | 2.8×10^-166^ | 0.07 | 0.01 | 1.2×10^-13^ | 1 | intron_variant | | protein_coding | *APOC1* |  | |  |  |
| 19 | chr19:44918715:AG:A (rs12721052) | A | 0.32 | -0.18 | 0.01 | 2.9×10^-36^ | -0.1 | 0.01 | 2.4×10^-13^ | 1 | intron_variant | | protein_coding | *APOC1* | *APOC1P1* | | *TOMM40* |  |
| 19 | chr19:44925202:C:T (rs4803772) | T | 0.31 | -0.16 | 0.01 | 2.2×10^-40^ | -0.09 | 0.01 | 4×10^-13^ | 1 | upstream_gene_variant | | lncRNA | *APOC1P1* | *APOC1P1* | | *TOMM40* |  |
| 19 | chr19:44920379:G:A (rs78959900) | A | 0.32 | -0.16 | 0.01 | 1.6×10^-39^ | -0.09 | 0.01 | 1.4×10^-12^ | 1 | downstream_gene_variant | | protein_coding | *APOC1* |  | |  |  |
| 19 | chr19:44918487:G:T (rs12721056) | T | 0.32 | -0.16 | 0.01 | 3×10^-39^ | -0.09 | 0.01 | 2.2×10^-12^ | 1 | intron_variant | | protein_coding | *APOC1* |  | |  |  |
| 19 | chr19:44834661:G:A (rs147711004) | A | 0.04 | 0.43 | 0.03 | 2.7×10^-47^ | 0.2 | 0.03 | 5×10^-11^ | 1 |  | |  |  |  | |  |  |
| 19 | chr19:44915229:G:A (rs12691088) | A | 0.03 | 0.51 | 0.04 | 7.3×10^-43^ | 0.24 | 0.04 | 6.1×10^-11^ | 1 | intron_variant | | protein_coding | *APOC1* |  | |  |  |
| 19 | chr19:44887076:A:G (rs283815) | G | 0.27 | 0.26 | 0.01 | 3.3×10^-187^ | 0.06 | 0.01 | 9.5×10^-11^ | 1 | intron_variant | | protein_coding | *NECTIN2* |  | | *TOMM40* |  |
| 19 | chr19:44892962:C:T (rs157582) | T | 0.27 | 0.26 | 0.01 | 1.8×10^-187^ | 0.06 | 0.01 | 9.6×10^-11^ | 1 | downstream_gene_variant | | protein_coding | *NECTIN2* |  | | *TOMM40* |  |
| 19 | chr19:44891712:T:G (rs184017) | G | 0.27 | 0.26 | 0.01 | 8×10^-181^ | 0.06 | 0.01 | 3.4×10^-10^ | 1 | downstream_gene_variant | | protein_coding | *NECTIN2* |  | | *TOMM40* |  |
| 19 | chr19:44901434:A:G (rs405697) | A | 0.25 | -0.15 | 0.01 | 2.7×10^-30^ | -0.08 | 0.01 | 1.1×10^-09^ | 1 |  | |  |  |  | |  |  |
| 19 | chr19:44862190:G:A (rs146275714) | A | 0.02 | 0.48 | 0.04 | 1.5×10^-36^ | 0.23 | 0.04 | 2.3×10^-09^ | 1 | intron_variant | | protein_coding | *NECTIN2* |  | |  |  |
| 19 | chr19:44935297:C:T (rs7254133) | T | 0.31 | 0.15 | 0.01 | 2.6×10^-33^ | 0.07 | 0.01 | 2.9×10^-09^ | 1 | downstream_gene_variant | | lncRNA | *APOC1P1* |  | |  |  |
| 19 | chr19:44926286:C:T (rs71352239) | T | 0.29 | -0.13 | 0.01 | 1.8×10^-23^ | -0.07 | 0.01 | 9.2×10^-09^ | 1 | upstream_gene_variant | | lncRNA | *APOC1P1* |  | |  |  |
| 19 | chr19:44893408:G:T | T | 0.25 | 0.43 | 0.01 | 1.7×10^-246^ | 0.23 | 0.01 | 1.8×10^-66^ | 2 | downstream_gene_variant | | protein_coding | *NECTIN2* |  | |  |  |
| 19 | chr19:44885243:A:G | G | 0.27 | 0.41 | 0.01 | 7.7×10^-218^ | 0.22 | 0.01 | 2.4×10^-58^ | 2 | intron_variant | | protein_coding | *NECTIN2* |  | |  |  |
| 19 | chr19:44893716:G:A (rs77301115) | A | 0.04 | 0.55 | 0.03 | 6.3×10^-80^ | 0.3 | 0.03 | 1.2×10^-24^ | 2 | downstream_gene_variant | | protein_coding | *NECTIN2* |  | |  |  |
| 19 | chr19:44895528:C:T (rs79398853) | T | 0.04 | 0.55 | 0.03 | 1.7×10^-79^ | 0.3 | 0.03 | 1.6×10^-24^ | 2 | intron_variant | | protein_coding | *TOMM40* |  | |  |  |
| 19 | chr19:44896087:G:T (rs75687619) | T | 0.04 | 0.55 | 0.03 | 1.5×10^-79^ | 0.3 | 0.03 | 1.9×10^-24^ | 2 | intron_variant | | protein_coding | *TOMM40* |  | |  |  |
| 19 | chr19:44897468:C:T (rs114536010) | T | 0.04 | 0.55 | 0.03 | 2.6×10^-79^ | 0.3 | 0.03 | 2.1×10^-24^ | 2 | intron_variant | | protein_coding | *TOMM40* |  | |  |  |
| 19 | chr19:44896639:G:A (rs76366838) | A | 0.04 | 0.55 | 0.03 | 3.2×10^-79^ | 0.3 | 0.03 | 2.8×10^-24^ | 2 | intron_variant | | protein_coding | *TOMM40* |  | |  |  |
| 19 | chr19:44894050:C:T (rs112849259) | T | 0.04 | 0.55 | 0.03 | 4.4×10^-79^ | 0.3 | 0.03 | 3.3×10^-24^ | 2 | downstream_gene_variant | | protein_coding | *NECTIN2* |  | |  |  |
| 19 | chr19:44909698:A:C (rs1081105) | C | 0.04 | 0.55 | 0.03 | 8.5×10^-83^ | 0.29 | 0.03 | 6.4×10^-24^ | 2 | upstream_gene_variant | | protein_coding | *APOC1* |  | |  |  |
| 19 | chr19:44901600:T:C (rs112019714) | C | 0.04 | 0.54 | 0.03 | 2.2×10^-80^ | 0.29 | 0.03 | 6.6×10^-24^ | 2 | upstream_gene_variant | | protein_coding | *APOE* |  | |  |  |
| 19 | chr19:44894695:T:C (rs116881820) | C | 0.04 | 0.55 | 0.03 | 1.8×10^-78^ | 0.3 | 0.03 | 7.9×10^-24^ | 2 | intron_variant | | protein_coding | *TOMM40* |  | |  |  |
| 19 | chr19:44883377:C:T (rs147636938) | T | 0.03 | 0.55 | 0.03 | 2.5×10^-73^ | 0.31 | 0.03 | 1.2×10^-23^ | 2 | intron_variant | | protein_coding | *NECTIN2* |  | |  |  |
| 19 | chr19:44899959:C:T (rs115881343) | T | 0.04 | 0.54 | 0.03 | 6.4×10^-80^ | 0.29 | 0.03 | 1.4×10^-23^ | 2 | intron_variant | | protein_coding | *TOMM40* |  | |  |  |
| 19 | chr19:44921809:G:A (rs188535946) | A | 0.04 | 0.55 | 0.03 | 2.6×10^-73^ | 0.3 | 0.03 | 3.1×10^-23^ | 2 | downstream_gene_variant | | protein_coding | *APOC1* |  | |  |  |
| 19 | chr19:44917947:C:T (rs150966173) | T | 0.04 | 0.52 | 0.03 | 2×10^-75^ | 0.27 | 0.03 | 1×10^-21^ | 2 | intron_variant | | protein_coding | *APOC1* |  | |  |  |
| 19 | chr19:44918393:G:A (rs140480140) | A | 0.04 | 0.53 | 0.03 | 1.4×10^-71^ | 0.29 | 0.03 | 2×10^-21^ | 2 | intron_variant | | protein_coding | *APOC1* |  | |  |  |
| 19 | chr19:44903416:G:A (rs10119) | A | 0.33 | 0.25 | 0.01 | 9.9×10^-179^ | 0.08 | 0.01 | 6.1×10^-20^ | 2 | upstream_gene_variant | | protein_coding | *APOE* | *NECTIN2* | | *TOMM40* |  |
| 19 | chr19:44898611:T:C (rs118170342) | C | 0.05 | 0.43 | 0.03 | 1.7×10^-60^ | 0.22 | 0.03 | 4.6×10^-17^ | 2 | intron_variant | | protein_coding | *TOMM40* |  | |  |  |
| 19 | chr19:44890259:C:T (rs117310449) | T | 0.02 | 0.58 | 0.05 | 9×10^-31^ | 0.33 | 0.05 | 4.8×10^-11^ | 2 | downstream_gene_variant | | protein_coding | *NECTIN2* | *NECTIN2* | |  |  |
| 19 | chr19:44882099:C:A (rs144261139) | A | 0.02 | 0.56 | 0.05 | 1.8×10^-29^ | 0.32 | 0.05 | 1.9×10^-10^ | 2 | intron_variant | | protein_coding | *NECTIN2* |  | |  |  |
| 19 | chr19:44913484:C:T (rs438811) | C | 0.41 | -0.23 | 0.01 | 1.8×10^-139^ | -0.15 | 0.01 | 1.6×10^-57^ | 3 |  | |  |  | *APOE* | | *TOMM40* |  |
| 19 | chr19:44870308:G:A (rs395908) | A | 0.16 | 0.05 | 0.01 | 7.8×10^-08^ | 0.06 | 0.01 | 1.2×10^-08^ | 3 | intron_variant | | protein_coding | *NECTIN2* | *NECTIN2, BCAM* | |  |  |
| 19 | chr19:44869537:G:A (rs404935) | A | 0.16 | 0.06 | 0.01 | 2.2×10^-08^ | 0.06 | 0.01 | 3.6×10^-09^ | 4 | intron_variant | | protein_coding | *NECTIN2* | *NECTIN2, BCAM* | |  |  |
| **Table S9: Genome-wide significant SNPs from *APOE* conditional analysis performed using COJO.** This table summarizes the association statistics for SNPs that passed genome-wide significance in *APOE* conditional analysis where results were adjusted for top hit rs429358 (chr19:44908684:T:C) and rs7412 (chr19:44908822:C:T) that codifies *APOE* ɛ4 and *APOE* ɛ2, respectively, from the multi-ethnic meta-analysis. Columns are chromosome (Chr); SNP; effect allele (refA); frequency of effect allele in the multi-ethnic data (freq); effect size (b), standard error (se), and p-value from the original multi-ethnic meta-analysis (p); effect size (bC), standard error (bC_se), p-value (pC), and group (Group) from the *APOE* conditional analyses. Here, group 2, 3, and 4 represent the SNPs that passed the genome-wide significant when the top hits from each of the conditional analysis (rs73052335, rs1081105, and rs438811, respectively) were added to the subsequent conditional analysis, whereas group 1 is the main *APOE*-conditional analysis on *APOE* ɛ4 and *APOE* ɛ2 SNPs. Consequence, Biotype, and Symbol represent the functional annotation of SNPs using VEP tool. eQTL and sQTL represent the expression- and splicing-QTLs for genes whose mRNA levels colocalize with these SNPs based on the GTEx data. | | | | | | | | | | | | | | | | | | |

| **# SNPs** | **PP.H0.abf** | **PP.H1.abf** | **PP.H2.abf** | **PP.H3.abf** | **PP.H4.abf** | **SNP** |
| --- | --- | --- | --- | --- | --- | --- |
| 51 | 0.00 | 0.00 | 0.98 | 0.00 | 0.02 | chr19:44834661:G:A |
| 55 | 0.00 | 0.00 | 0.00 | 0.00 | 1.00 | chr19:44862190:G:A |
| 58 | 0.00 | 0.98 | 0.00 | 0.00 | 0.01 | chr19:44869537:G:A |
| 36 | 0.01 | 0.98 | 0.00 | 0.00 | 0.01 | chr19:44870308:G:A |
| 52 | 0.00 | 0.00 | 0.00 | 0.00 | 1.00 | chr19:44882099:C:A |
| 38 | 0.00 | 0.00 | 0.00 | 0.00 | 1.00 | chr19:44883210:G:GTAA |
| 37 | 0.00 | 0.00 | 0.00 | 0.00 | 1.00 | chr19:44883377:C:T |
| 57 | 0.00 | 0.00 | 0.02 | 0.00 | 0.98 | chr19:44885243:A:G |
| 36 | 1.00 | 0.00 | 0.00 | 0.00 | 0.00 | chr19:44887076:A:G |
| 38 | 1.00 | 0.00 | 0.00 | 0.00 | 0.00 | chr19:44888997:C:T |
| 33 | 0.00 | 0.00 | 0.00 | 0.00 | 1.00 | chr19:44890259:C:T |
| 47 | 0.00 | 0.00 | 0.15 | 0.01 | 0.85 | chr19:44891712:T:G |
| 45 | 0.00 | 0.00 | 0.00 | 1.00 | 0.00 | chr19:44892587:G:A |
| 53 | 0.00 | 0.00 | 0.00 | 1.00 | 0.00 | chr19:44892962:C:T |
| 53 | 0.00 | 0.00 | 0.00 | 0.00 | 1.00 | chr19:44893408:G:T |
| 54 | 0.00 | 0.00 | 0.00 | 0.00 | 1.00 | chr19:44893716:G:A |
| 37 | 0.00 | 0.00 | 0.00 | 0.00 | 1.00 | chr19:44894050:C:T |
| 32 | 0.00 | 0.00 | 0.00 | 0.00 | 1.00 | chr19:44894695:T:C |
| 50 | 0.00 | 0.00 | 0.00 | 1.00 | 0.00 | chr19:44895528:C:T |
| 58 | 0.99 | 0.00 | 0.00 | 0.00 | 0.00 | chr19:44896087:G:T |
| 47 | 0.99 | 0.00 | 0.00 | 0.00 | 0.00 | chr19:44896639:G:A |
| 44 | 0.00 | 0.00 | 0.00 | 0.00 | 1.00 | chr19:44897468:C:T |
| 54 | 0.00 | 0.00 | 0.00 | 1.00 | 0.00 | chr19:44898611:T:C |
| 47 | 0.00 | 0.00 | 0.09 | 0.00 | 0.91 | chr19:44899220:C:T |
| 46 | 0.00 | 0.00 | 0.00 | 0.00 | 1.00 | chr19:44899959:C:T |
| 50 | 0.00 | 0.00 | 0.00 | 1.00 | 0.00 | chr19:44901434:A:G |
| 49 | 0.00 | 0.00 | 0.00 | 1.00 | 0.00 | chr19:44901600:T:C |
| 47 | 0.00 | 0.00 | 0.00 | 0.00 | 1.00 | chr19:44901715:T:C |
| 46 | 0.00 | 0.00 | 0.00 | 0.00 | 1.00 | chr19:44901805:A:G |
| 52 | 0.99 | 0.00 | 0.01 | 0.00 | 0.00 | chr19:44903281:C:CG |
| 55 | 0.99 | 0.00 | 0.01 | 0.00 | 0.00 | chr19:44903416:G:A |
| 43 | 1.00 | 0.00 | 0.00 | 0.00 | 0.00 | chr19:44904531:G:A |
| 64 | 0.00 | 0.00 | 0.00 | 0.00 | 1.00 | chr19:44909698:A:C |
| 43 | 0.00 | 0.00 | 0.00 | 0.00 | 1.00 | chr19:44912678:G:T |
| 45 | 0.00 | 0.00 | 0.01 | 0.00 | 0.99 | chr19:44913484:C:T |
| 44 | 0.00 | 0.00 | 0.00 | 0.00 | 1.00 | chr19:44915229:G:A |
| 49 | 0.00 | 0.00 | 0.00 | 0.00 | 1.00 | chr19:44915533:T:C |
| 48 | 0.00 | 0.00 | 0.00 | 0.00 | 1.00 | chr19:44915704:T:C |
| 57 | 0.99 | 0.00 | 0.00 | 0.00 | 0.00 | chr19:44916825:A:C |
| 58 | 0.00 | 0.00 | 0.00 | 1.00 | 0.00 | chr19:44917843:G:A |
| 55 | 0.00 | 0.00 | 0.00 | 1.00 | 0.00 | chr19:44917947:C:T |
| 42 | 0.00 | 0.00 | 0.00 | 1.00 | 0.00 | chr19:44918393:G:A |
| 39 | 0.00 | 0.00 | 0.00 | 1.00 | 0.00 | chr19:44918487:G:T |
| 46 | 0.00 | 0.00 | 0.00 | 0.00 | 1.00 | chr19:44918715:AG:A |
| 42 | 0.00 | 0.00 | 0.00 | 0.00 | 1.00 | chr19:44920379:G:A |
| 36 | 0.00 | 0.00 | 0.02 | 0.00 | 0.98 | chr19:44921809:G:A |
| 37 | 0.00 | 0.00 | 0.00 | 0.00 | 1.00 | chr19:44924977:G:A |
| 39 | 0.00 | 0.00 | 0.00 | 0.00 | 1.00 | chr19:44925202:C:T |
| 44 | 0.00 | 0.00 | 0.00 | 0.00 | 1.00 | chr19:44926286:C:T |
| 34 | 0.00 | 0.00 | 0.19 | 0.00 | 0.81 | chr19:44935297:C:T |
| **Table S10: Colocalization of hits from *APOE-ε4 and ε2*-conditional analysis with Schwartzentrauber AD risk GWAS** [4]**.** Total SNPs represents the number of SNP in the considered region (SNP ± 500 bp) from Schwartzentrauber GWAS that are tested for colocalization with our SNP of interest mentioned in the last column (SNP). PP represent the posterior probability of: neither trait has a genetic association in the region (H0), only trait 1 has a genetic association in the region (H1), only trait 2 has a genetic association in the region (H2), both traits are associated, but with different causal variants (H3), and both traits are associated and share a single causal variant (H4). | | | | | | |

| **SNP Pair** | **R2** | **D’** |
| --- | --- | --- |
| rs429358_ rs7412 (*APOE* ɛ4_ ɛ2) | 0.0041 | 0.9498 |
| rs429358_ rs1081105 (ɛ4_cond1) | 0.0915 | 0.4056 |
| rs429358_ rs438811 (ɛ4_cond2) | 0.1239 | 0.9267 |
| rs429358_ rs4420638 (ɛ4_cond3) | 0.063 | 0.4486 |
| rs7412_ rs1081105 (ɛ2_cond1) | 0.0025 | 1 |
| rs7412_ rs438811 (ɛ2_cond2) | 0.1989 | 0.9742 |
| rs7412_rs4420638 (ɛ2_cond3) | 0.0035 | 0.4906 |
| rs73052335_ rs5117 | 0.2256 | 0.9197 |
| **Table S11: Linkage disequilibrium (LD) between pairs of SNP from the APOE conditional analysis**. This table summarize the LD between *APOE* ɛ4 (rs429358), *APOE* ɛ2 (rs7412), and independent signals (rs5117, rs73052335, rs1081105, rs438811, rs4420638) from the APOE conditional analysis. | | |

| **SNP** | **NHW** | | | **AFR** | | | **ASN** | | |
| --- | --- | --- | --- | --- | --- | --- | --- | --- | --- |
|  | **BETA [SE]** | **P** | **MAF** | **BETA [SE]** | **P** | **MAF** | **BETA [SE]** | **P** | **MAF** |
| **rs429358** | 0.62 [0.01] | **1.8×10^-416^** | 0.19 | 0.49 [0.07] | **1.0×10^-11^** | 0.21 | 0.14* [0.01] | **6.7×10^-28^** | 0.14 |
| **rs7412** | -0.33 [0.02] | **6.1×10^-42^** | 0.07 | -0.16 [0.10] | 0.11 | 0.10 | -0.02 [0.03] | 0.60 | 0.03 |
| **rs5117** | 0.41* [0.01] | **9.6×10^-212^** | 0.29 | 0.04 [0.06] | 0.50 | 0.33 | 0.11 [0.01] | **5.3×10^-17^** | 0.19 |
| **rs73052335** | 0.56 [0.02] | **1.7×10^-270^** | 0.18 | 0.45 [0.14] | 0.002 | 0.04 | 0.43 [0.07] | **1.3×10^-09^** | 0.13 |
| **Table S12: Race-specific *APOE* effect.** The race-specific effect of *APOE* ε4 (rs429358/chr19:44908684:T:C), *APOE* ε2 (rs7412/chr19:44908822:C:T), the top hit (rs5117/chr19:44915533:T:C) from *APOE* conditional analysis using *APOE* genotype as covariate, and top hit (rs73052335/chr19:44916825:A:C) from *APOE* conditional analysis using *APOE* ε4 and *APOE* ε2 variants as covariate in COJO are summarized from race-specific meta-analyses. BETA, SE, P, and MAF represent the effect estimate, standard error, p-value, and minor allele frequency. * denotes the significant difference (P < 0.05) in the effect size (BETA) of an ancestry with respect to all other ancestries, e.g. for rs429358, the two sample t-test showed significant difference in the BETA estimates of ASN ancestry with NHW (P < 3.5×10^-242^) and AFR (P < 7.4×10^-07^) ancestries. In case of rs7412, the difference in BETA was significant only between NHW and ASN (P < 8.1×10^-18^), however, rs5117 showed significantly different BETA in NHW as compared to AFR (P < 3.3×10^-09^) and ASN (P < 1.9×10^-93^) ancestries. | | | | | | | | | |

|  | **Meta-analyses** | |  | **APOE interaction** | |  | ***APOE* ɛ4+** | |  | ***APOE* ɛ4-** | |  | **ɛ4+ vs. ɛ4-** |  |
| --- | --- | --- | --- | --- | --- | --- | --- | --- | --- | --- | --- | --- | --- | --- |
| **SNP (Gene)** | **β (SE)** | **P** |  | **Β (SE)** | **P** |  | **β (SE)** | **P** |  | **β (SE)** | **P** |  | **T-test (β)** |  |
| chr1:207518704:A:G  (*CR1*) | 0.10 (0.02) | **2.4×10^-10^** |  | 0.05 (0.02) | **0.008** |  | 0.15 (0.03) | **3.3×10^-08^** |  | 0.04 (0.02) | 0.05 |  | **0.001*** |  |
| chr14:52848729:C:T  (*FERMT2*) | 0.16 (0.03) | **1.1×10^-09^** |  | -0.04 (0.03) | 0.20 |  | 0.11 (0.05) | 0.02 |  | 0.16 (0.03) | **7.3×10^-06^** |  | 0.38 |  |
| chr19:1050875:A:G  (*ABCA7*) | 0.07 (0.01) | **9.2×10^-09^** |  | -0.005 (0.02) | 0.74 |  | 0.05 (0.02) | 0.02 |  | 0.10 (0.02) | **3.3×10^-09^** |  | 0.10 |  |
| **Table S13: A comparison of effect size and p-value for genome-wide significant hits from multi-ethnic meta-analysis**: This table summarizes the comparison of effect size (β) and p-value (P) for genome-wide significant hits from the multi-ethnic meta-analysis. We checked the β and P for these SNPs in the multi-ethnic meta-analysis (ME), APOE-interaction model (APOE-inter), and *APOE* ɛ4- stratified (*APOE* ɛ4+ and *APOE* ɛ4-) analysis. We also compared the β between *APOE* ɛ4-stratified analyses using the two sample t-test and reported the p-value representing the significance of β difference. | | | | | | | | | | | | | | |

| **SNP** | | **rsID** | **Population (MAF)** | **R2** | **A1** | **A2** | **BETA** | **SE** | **P** |
| --- | --- | --- | --- | --- | --- | --- | --- | --- | --- |
| **chr5:25917854:G:A** | **rs529007143** | | **Female (NHW: 0.006)** | **1** | **G** | **A** | **0.794** | **0.140** | **1.5×10^-08^** |
| chr5:25877050:G:A | rs116801572 | | NHW (0.010) | 1 | G | A | 0.763 | 0.143 | 1.04×10^-07^ |
| chr5:25859458:A:G | rs188723910 | | NHW (0.010) | 1 | A | G | 0.749 | 0.140 | 9.4×10^-08^ |
| **chr11:15354595:A:G** | **rs192346166** | | **Female (NHW: 0.004)** | **1** | **A** | **G** | **0.943** | **0.171** | **3.99×10^-08^** |
| **Table S14: Association statistics for SNPs in high linkage disequilibrium with genome-wide significant hits from sex-stratified (female) GWAS.** Actual SNPs are highlighted with blue color and other SNPs in high LD (R2 > 0.9) are highlighted with black color. No SNP was found to be in high LD with hit from chr11.on *INSC* locus | | | | | | | | | |

| **# SNPs** | **PP.H0.abf** | **PP.H1.abf** | **PP.H2.abf** | **PP.H3.abf** | **PP.H4.abf** | **Tissue** | **Gene** |
| --- | --- | --- | --- | --- | --- | --- | --- |
| 287 | 1.4×10^-26^ | 1.9×10^-28^ | 0.897 | 0.012 | 0.090 | Whole_Blood | *COPB1* |
| 217 | 0.016 | 0.0002 | 0.924 | 0.008 | 0.049 | Brain_Cerebellum | *COPB1* |
| 95 | 3.6×10^-13^ | 1.1×10^-15^ | 0.964 | 0.003 | 0.032 | Whole_Blood | *INSC* |
| 221 | 1.3×10^-17^ | 8.6×10^-20^ | 0.974 | 0.006 | 0.019 | Whole_Blood | *ENSG00000254789* |
| 71 | 0.410 | 0.0009 | 0.570 | 0.001 | 0.017 | Brain_Cerebellar_Hemisphere | *COPB1* |
| 85 | 0.0002 | 4.8×10^-07^ | 0.980 | 0.002 | 0.016 | Brain_Cerebellum | *CALCB* |
| 135 | 6.5×10^-07^ | 2.8×10^-09^ | 0.979 | 0.004 | 0.016 | Brain_Cerebellar_Hemisphere | *CALCB* |
| 93 | 0.263 | 0.0005 | 0.720 | 0.001 | 0.013 | Brain_Cerebellum | *INSC* |
| 77 | 0.400 | 0.0007 | 0.584 | 0.001 | 0.013 | Brain_Cerebellum | *RRAS2* |
| 19 | 0.479 | 0.0001 | 0.509 | 0.0001 | 0.010 | Brain_Caudate_basal_ganglia | *RRAS2* |
| 46 | 0.599 | 0.0005 | 0.390 | 0.0003 | 0.008 | Brain_Cerebellar_Hemisphere | *OR7E41P* |
| 28 | 0.769 | 0.0004 | 0.225 | 0.0001 | 0.004 | Brain_Cerebellar_Hemisphere | *INSC* |
| 1 | 0.996 | 4.9×10^-05^ | 0.003 | 0 | 0.0001 | Whole_Blood | *RRAS2* |
| 2 | 0.998 | 0.0005 | 0.0006 | 1.8×10^-07^ | 0.0001 | Brain_Cerebellum | *ENSG00000254645* |
| 6 | 0.998 | 0.0004 | 0.001 | 4.9×10^-07^ | 9.98×10^-05^ | Brain_Hypothalamus | *CALCA* |
| **Table S15: Colocalization of hit from female-stratified analyses on chr11p.15.2 (rs192346166) with GTEx data.** Total SNPs represent the number of SNP in the considered region that are tested for colocalization with our SNP of interest (rs192346166/chr11:15354595:A:G). PP represent the posterior probability of: neither trait has a genetic association in the region (H0), only trait 1 has a genetic association in the region (H1), only trait 2 has a genetic association in the region (H2), both traits are associated, but with different causal variants (H3), and both traits are associated and share a single causal variant (H4). | | | | | | | |

|  | **Meta-analyses** | | **AD cases (AD)** | | **Controls (CO)** | | **AD vs. CO** |
| --- | --- | --- | --- | --- | --- | --- | --- |
| **SNP (Gene)** | **β (SE)** | **P** | **β (SE)** | **P** | **β (SE)** | **P** | **T-test (β)** |
| chr19:44908684:T:C  (*APOE* ɛ4) | 0.35 (0.01) | **6.2×10^-311^** | 0.59 (0.02) | **1.2×10^-165^** | 0.47 (0.04) | **1.9×10^-29^** | **0.007** |
| chr19:44908822:C:T  (*APOE* ɛ2) | -0.21 (0.02) | **1.8×10^-28^** | -0.54 (0.10) | **3.7×10^-08^** | -0.26 (0.03) | **2.1×10^-16^** | **0.007** |
| chr1:207518704:A:G  (*CR1*) | 0.10 (0.02) | **2.4×10^-10^** | 0.08 (0.02) | 0.12 | 0.05 (0.02) | **0.02** | 0.29 |
| chr14:52848729:C:T  (*FERMT2*) | 0.16 (0.03) | **1.1×10^-09^** | 0.14 (0.08) | 0.09 | 0.10 (0.04) | **0.005** | 0.65 |
| chr19:1050875:A:G  (*ABCA7*) | 0.07 (0.01) | **9.2×10^-09^** | 0.09 (0.04) | 0.04 | 0.05 (0.02) | **0.002** | 0.37 |
| **Table S16: A comparison of effect size and p-value for genome-wide significant hits from multi-ethnic meta-analysis in the case-control stratified analyses**: This table summarizes the comparison of effect size (β) and p-value (P) for genome-wide significant hits from the multi-ethnic meta-analysis in the case-control stratified analyses. We checked the β and P for these SNPs in the multi-ethnic meta-analysis (ME) and case-control stratified (AD and CO) analyses. We also compared the β between case-control stratified analyses using the two sample t-test and reported the p-value representing the significance of β difference. | | | | | | | |

| **MarkerName** | **rsID** | **A1** | **A2** | **P**  **AmyloidPET** | **P**  **Bellenguez** | **Beta**  **AmyloidPET** | **Beta**  **Bellenguez** | **Analyses** | **Gene** |
| --- | --- | --- | --- | --- | --- | --- | --- | --- | --- |
| chr14:92464917:G:A | rs7401792 | A | G | 0.001 | 7.15×10^-06^ | 0.14 | -0.04 | AD | *SLC24A4* |
| chr14:92472511:G:A | rs12590654 | A | G | 0.009 | 2.08×10^-15^ | -0.11 | -0.07 | AD | *SLC24A4* |
| chr17:63471557:C:T | rs4277405 | T | C | 0.012 | 7.24×10^-16^ | 0.11 | 0.07 | AD | *ACE* |
| chr21:26101558:C:T | rs2154481 | T | C | 0.017 | 1.02×10^-09^ | 0.1 | 0.05 | AD | *APP* |
| chr8:27607795:T:C | rs11787077 | T | C | 0.02 | 2.43×10^-33^ | -0.1 | -0.1 | AD | *CLU* |
| chr14:52924962:A:G | rs17125924 | A | G | 0.033 | 5.82×10^-10^ | -0.15 | -0.09 | AD | *FERMT2* |
| chr19:1050875:A:G | rs12151021 | A | G | 0.039 | 4.09×10^-30^ | 0.09 | 0.11 | AD | *ABCA7* |
| chr7:7817263:T:C | rs6943429 | T | C | 0.001 | 2.98×10^-07^ | 0.05 | 0.04 | CO | *UMAD1* |
| chr15:63277703:C:T | rs117618017 | T | C | 0.001 | 1.75×10^-21^ | 0.08 | 0.11 | CO | *APH1B* |
| chr8:27607795:T:C | rs11787077 | T | C | 0.001 | 2.43×10^-33^ | -0.05 | -0.1 | CO | *CLU* |
| chr19:1050875:A:G | rs12151021 | A | G | 0.002 | 4.09×10^-30^ | 0.05 | 0.11 | CO | *ABCA7* |
| chr11:121564878:T:C | rs11218343 | T | C | 0.013 | 1.01×10^-14^ | 0.1 | 0.17 | CO | *SORL1* |
| chr6:32615322:A:G | rs6605556 | A | G | 0.014 | 1.05×10^-17^ | -0.06 | 0.1 | CO | *HLA-DQA1* |
| chr2:127135234:C:T | rs6733839 | T | C | 0.016 | 6.48×10^-90^ | 0.04 | 0.17 | CO | *BIN1* |
| chr10:80494228:C:T | rs6586028 | T | C | 0.017 | 1.33×10^-14^ | 0.05 | 0.08 | CO | *TSPAN14* |
| chr1:207577223:T:C | rs679515 | T | C | 0.018 | 5.15×10^-33^ | 0.05 | 0.12 | CO | *CR1* |
| chr17:49219935:T:C | rs616338 | T | C | 0.026 | 5.38×10^-13^ | 0.21 | 0.29 | CO | *ABI3* |
| chr11:121482368:T:G | rs74685827 | T | G | 0.028 | 8.63×10^-11^ | -0.14 | -0.2 | CO | *SORL1* |
| **Table S17: Overlap of between AD risk genes identified by Bellenguez AD case-control GWAS and amyloid PET multi-ethnic case (N=1,138)-control (N=5,846) stratified analyses.** This table summarizes the list of variants that has been associated with AD risk genes according to Bellenguez case-control AD GWAS [5]. We also report the effect size and P-value for these variants from our case-control stratified analyses. | | | | | | | | | |

| **MarkerName** | **rsID** | **A1** | **A2** | **P**  **AmyloidPET** | **P**  **Bellenguez** | **Beta**  **AmyloidPET** | **Beta**  **Bellenguez** | **Gene** |
| --- | --- | --- | --- | --- | --- | --- | --- | --- |
| chr1:207577223:T:C | rs679515 | T | C | **1.2×10^-09^** | **5.2×10^-33^** | 0.093 | 0.124 | ***CR1*** |
| chr19:1050875:A:G | rs12151021 | A | G | **1.1×10^-08^** | **4.1×10^-30^** | 0.07 | 0.106 | ***ABCA7*** |
| chr14:52924962:A:G | rs17125924 | G | A | **8.6×10^-06^** | **5.8×10^-10^** | 0.052 | -0.088 | ***FERMT2*** |
| chr15:63277703:C:T | rs117618017 | T | C | **2.9×10^-05^** | **1.7×10^-21^** | 0.073 | 0.113 | ***APH1B*** |
| chr11:86157598:T:C | rs3851179 | T | C | **7.8×10^-05^** | **6.5×10^-36^** | -0.035 | -0.105 | ***EED*** |
| chr17:63471557:C:T | rs4277405 | C | T | **1.0×10^-04^** | **7.2×10^-16^** | -0.047 | 0.068 | ***ACE*** |
| chr8:27607795:T:C | rs11787077 | T | C | **1.7×10^-04^** | **2.4×10^-33^** | -0.035 | -0.1 | ***CLU*** |
| chr2:127135234:C:T | rs6733839 | T | C | **2.1×10^-04^** | **6.5×10^-90^** | 0.032 | 0.169 | ***BIN1*** |
| chr11:121564878:T:C | rs11218343 | C | T | **1.5×10^-03^** | **1.0×10-14** | -0.086 | 0.165 | ***SORL1*** |
| chr10:60025170:T:G | rs7068231 | T | G | **2.3×10^-03^** | **6.8×10^-09^** | -0.027 | -0.049 | ***ANK3*** |
| chr6:41161514:C:T | rs75932628 | T | C | **2.5×10^-03^** | **1.4×10^-27^** | 0.369 | 0.884 | ***TREM2*** |
| chr21:26101558:C:T | rs2154481 | C | T | **2.8×10^-03^** | **1.0×10^-09^** | -0.039 | 0.05 | ***APP*** |
| chr17:49219935:T:C | rs616338 | T | C | **4.8×10^-03^** | **5.4×10^-13^** | 0.247 | 0.294 | ***ABI3*** |
| chr7:7817263:T:C | rs6943429 | T | C | **1.0×10^-02^** | **3.0×10^-07^** | 0.03 | 0.043 | ***UMAD1*** |
| chr14:92472511:G:A | rs12590654 | A | G | **1.5×10^-02^** | **2.1×10^-15^** | -0.03 | -0.069 | ***SLC24A4*** |
| chr5:180201150:G:A | rs113706587 | A | G | **1.8×10^-02^** | **3.4×10^-12^** | 0.044 | 0.093 | ***RASGEF1C*** |
| chr11:47370397:G:A | rs10437655 | A | G | **1.9×10^-02^** | **8.2×10^-12^** | 0.028 | 0.057 | ***SPI1*** |
| chr19:54267597:C:T | rs587709 | C | T | **2.0×10^-02^** | **3.9×10^-08^** | 0.031 | -0.055 | ***LILRB2*** |
| chr8:144103704:G:A | rs34173062 | A | G | **4.3×10^-02^** | **2.9×10^-12^** | -0.049 | 0.114 | ***SHARPIN*** |
| chr10:11676714:A:G | rs7912495 | G | A | **4.7×10^-02^** | **2.9×10^-12^** | 0.018 | -0.057 | ***USP6NL*** |
| chr11:121482368:T:G | rs74685827 | G | T | **4.9×10^-02^** | **8.6×10^-11^** | 0.097 | -0.196 | ***SORL1*** |
| chr16:70660097:C:A | rs4985556 | A | C | 5.7×10^-02^ | 5.6×10^-06^ | -0.035 | 0.058 | *IL34* |
| chr6:114291731:T:C | rs785129 | T | C | 8.3×10^-02^ | 2.9×10^-07^ | 0.021 | 0.044 | *HS3ST5* |
| chr10:122413396:A:G | rs7908662 | G | A | 9.9×10^-02^ | 3.3×10^-06^ | -0.014 | 0.038 | *PLEKHA1* |
| chr3:155069722:G:A | rs16824536 | A | G | 1.0×10^-01^ | 3.8×10^-06^ | 0.037 | -0.086 | *MME* |
| chr7:100334426:C:T | rs7384878 | C | T | 1.1×10^-01^ | 2.1×10^-18^ | -0.015 | 0.078 | *SPDYE3* |
| chr5:14724304:T:A | rs112403360 | A | T | 1.2×10^-01^ | 3.4×10^-06^ | 0.053 | 0.073 | *ANKH* |
| chr7:28129126:GTCTT:G | rs1160871 | G | GTCTT | 1.2×10^-01^ | 1.1×10^-07^ | -0.025 | -0.053 | *JAZF1* |
| chr10:80494228:C:T | rs6586028 | C | T | 1.4×10^-01^ | 1.3×10^-14^ | -0.023 | 0.079 | *TSPAN14* |
| chr20:63743088:T:C | rs6742 | T | C | 1.5×10^-01^ | 2.5×10^-06^ | -0.021 | -0.049 | *SLC2A4RG* |
| chr6:32615322:A:G | rs6605556 | G | A | 1.5×10^-01^ | 1.0×10^-17^ | 0.019 | 0.098 | *HLA-DQA1* |
| chr16:81739398:G:A | rs12446759 | G | A | 1.6×10^-01^ | 3.6×10^-12^ | -0.017 | 0.059 | *PLCG2* |
| chr12:113281983:T:C | rs6489896 | C | T | 1.8×10^-01^ | 2.5×10^-06^ | 0.017 | -0.073 | *TPCN1* |
| chr4:40197226:G:C | rs2245466 | C | G | 2.0×10^-01^ | 3.1×10^-07^ | -0.024 | -0.047 | *RHOH* |
| chr16:31111250:C:T | rs889555 | T | C | 2.1×10^-01^ | 1.0×10^-09^ | -0.018 | -0.056 | *BCKDK* |
| chr7:143413669:G:A | rs11771145 | A | G | 2.1×10^-01^ | 1.3×10^-12^ | -0.011 | -0.06 | *EPHA1* |
| chr17:58332680:A:G | rs2526377 | G | A | 2.1×10^-01^ | 4.1×10^-08^ | -0.011 | 0.045 | *TSPOAP1* |
| chr8:27362470:C:T | rs73223431 | T | C | 2.3×10^-01^ | 5.3×10^-15^ | 0.011 | 0.066 | *PTK2B* |
| chr19:1854254:G:GC | rs149080927 | G | GC | 2.3×10^-01^ | 6.1×10^-09^ | 0.03 | 0.054 | *KLF16* |
| chr6:41161469:C:T | rs143332484 | T | C | 2.5×10^-01^ | 6.0×10^-19^ | 0.087 | 0.335 | *TREM2* |
| chr9:104903697:C:G | rs1800978 | C | G | 2.9×10^-01^ | 1.6×10^-08^ | -0.027 | -0.068 | *ABCA1* |
| chr14:105761758:A:G | rs7157106 | A | G | 3.0×10^-01^ | 1.5×10^-07^ | 0.02 | 0.056 | *IGH gene cluster* |
| chr21:26775872:C:T | rs2830489 | T | C | 3.0×10^-01^ | 1.7×10^-09^ | -0.013 | -0.055 | *ADAMTS1* |
| chr2:233117202:G:C | rs10933431 | C | G | 3.4×10^-01^ | 1.0×10^-17^ | 0.02 | 0.085 | *INPP5D* |
| chr7:12229967:C:A | rs13237518 | A | C | 3.4×10^-01^ | 5.1×10^-07^ | 0.011 | -0.041 | *TMEM106B* |
| chr15:58764824:T:A | rs602602 | A | T | 3.4×10^-01^ | 9.7×10-12 | -0.018 | -0.062 | *MINDY2* |
| chr11:60254475:G:A | rs1582763 | A | G | 3.6×10^-01^ | 1.7×10-24 | -0.009 | -0.086 | *MS4A4A* |
| chr17:18156140:G:A | rs2242595 | A | G | 3.7×10^-01^ | 4.6×10^-06^ | -0.016 | -0.059 | *MYO15A* |
| chr3:155084189:A:G | rs61762319 | G | A | 4.1×10^-01^ | 2.1×10^-08^ | -0.032 | -0.143 | *MME* |
| chr17:1728046:TGAG:T | rs35048651 | T | TGAG | 4.8×10^-01^ | 4.8×10^-07^ | 0.021 | 0.051 | *WDR81* |
| chr20:56423488:A:G | rs6014724 | G | A | 4.8×10^-01^ | 4.8×10^-16^ | 0.008 | 0.118 | *CASS4* |
| chr2:202878716:TC:T | rs139643391 | T | TC | 4.9×10^-01^ | 2.6×10^-07^ | 0.014 | -0.063 | *WDR12* |
| chr2:9558882:A:G | rs72777026 | G | A | 5.1×10^-01^ | 1.9×10^-06^ | -0.011 | -0.056 | *ADAM17* |
| chr5:86927378:T:C | rs62374257 | C | T | 5.3×10^-01^ | 1.4×10^-13^ | 0.006 | -0.071 | *COX7C* |
| chr14:106665591:G:A | rs10131280 | A | G | 5.3×10^-01^ | 2.0×10^-07^ | 0.011 | -0.065 | *IGH gene cluster* |
| chr19:49950060:C:T | rs9304690 | T | C | 5.5×10^-01^ | 6.4×10^-06^ | 0.008 | 0.046 | *SIGLEC11* |
| chr16:30010081:C:T | rs1140239 | T | C | 5.6×10^-01^ | 4.6×10^-12^ | -0.007 | -0.059 | *DOC2A* |
| chr17:44352876:C:T | rs5848 | T | C | 5.8×10^-01^ | 1.8×10^-12^ | 0.005 | 0.065 | *GRN* |
| chr2:105749599:T:C | rs143080277 | C | T | 5.9×10^-01^ | 7.9×10^-11^ | -0.05 | -0.392 | *NCK2* |
| chr15:50701814:A:G | rs8025980 | G | A | 6.2×10^-01^ | 6.1×10^-06^ | 0.006 | 0.039 | *SPPL2A* |
| chr2:37304796:T:C | rs17020490 | C | T | 6.3×10^-01^ | 3.3×10^-06^ | 0.005 | -0.054 | *PRKD3* |
| chr16:86420604:T:A | rs16941239 | A | T | 6.5×10^-01^ | 2.9×10^-06^ | -0.022 | 0.12 | *FOXF1* |
| chr15:64131307:G:A | rs3848143 | G | A | 6.6×10^-01^ | 1.1×10^-06^ | -0.005 | -0.048 | *SNX1* |
| chr16:90103687:G:A | rs56407236 | A | G | 6.9×10^-01^ | 1.3×10^-11^ | -0.008 | 0.11 | *PRDM7* |
| chr14:92464917:G:A | rs7401792 | G | A | 7.2×10^-01^ | 7.1×10^-06^ | -0.003 | -0.038 | *SLC24A4* |
| chr5:151052827:C:T | rs871269 | T | C | 7.3×10^-01^ | 3.4×10^-06^ | -0.003 | -0.041 | *TNIP1* |
| chr4:993555:G:T | rs3822030 | T | G | 7.6×10^-01^ | 5.0×10^-10^ | -0.003 | 0.051 | *IDUA* |
| chr6:41181270:A:G | rs60755019 | G | A | 7.8×10^-01^ | 1.8×10^-07^ | 0.02 | -0.439 | *TREML2* |
| chr1:109345810:T:C | rs141749679 | C | T | 8.1×10^-01^ | 8.0×10^-06^ | -0.036 | -0.313 | *SORT1* |
| chr6:41036354:G:A | rs10947943 | A | G | 8.5×10^-01^ | 6.2×10^-06^ | -0.002 | -0.053 | *UNC5CL* |
| chr4:11023507:C:T | rs6846529 | C | T | 8.6×10^-01^ | 1.3×10^-13^ | 0.002 | -0.067 | *CLNK* |
| chr20:413334:A:G | rs1358782 | A | G | 8.6×10^-01^ | 1.9×10^-06^ | 0.002 | -0.047 | *RBCK1* |
| chr15:78936857:A:G | rs12592898 | A | G | 8.7×10^-01^ | 1.3×10^-06^ | -0.003 | -0.06 | *CTSH* |
| chr7:8204382:T:C | rs10952097 | T | C | 8.7×10^-01^ | 3.3×10^-07^ | 0.003 | 0.071 | *ICA1* |
| chr17:5233752:G:A | rs7225151 | A | G | 8.9×10^-01^ | 2.6×10^-12^ | 0.002 | 0.086 | *SCIMP* |
| chr7:54873635:C:T | rs76928645 | T | C | 8.9×10^-01^ | 1.5×10^-08^ | -0.003 | -0.076 | *SEC61G* |
| chr6:47517390:C:T | rs7767350 | T | C | 9.2×10^-01^ | 5.0×10^-12^ | 0.001 | 0.063 | *CD2AP* |
| chr16:79574511:T:C | rs450674 | C | T | 9.4×10^-01^ | 1.1×10^-07^ | 0.001 | 0.045 | *MAF* |
| chr8:11844613:G:C | rs1065712 | C | G | 9.5×10^-01^ | 5.5×10^-09^ | -0.002 | 0.108 | *CTSB* |
| chr10:96266650:G:A | rs6584063 | G | A | 9.6×10^-01^ | 1.8×10^-07^ | -0.002 | 0.112 | *BLNK* |
| chr17:46779275:G:C | rs199515 | C | G | 9.8×10^-01^ | 6.0×10^-09^ | 0.001 | 0.058 | *WNT3* |
| chr7:37844191:T:C | rs6966331 | T | C | 9.8×10^-01^ | 4.8×10^-06^ | 0 | -0.039 | EPDR1 |
| **Table S18: Overlap of between AD risk genes identified by Bellenguez AD case-control GWAS and amyloid PET multi-ethnic GWAS (N=13,409).** This table summarizes the list of variants that has been associated with AD risk genes according to Bellenguez case-control AD GWAS [5]. We also report the effect size and P-value for these variants from our multi-ethnic amyloid PET GWAS | | | | | | | | |

|  | **with *APOE*** | | | | **without *APOE*** | | | |
| --- | --- | --- | --- | --- | --- | --- | --- | --- |
| **Trait** | **corr** | **ci_upper** | **ci_lower** | **P.fdr** | **corr** | **ci_upper** | **ci_lower** | **P.fdr** |
| 25HydroxyVitaminD | 0.0066 | 0.0084 | -0.0075 | 0.9318 | 0.0109 | 0.0085 | -0.0071 | 0.8944 |
| ADHD | -0.0374 | 0.0073 | -0.0175 | 0.7351 | -0.0483 | 0.0061 | -0.0174 | 0.5866 |
| AgeAtMenarche | -0.0295 | 0.0043 | -0.0118 | 0.6577 | -0.0317 | 0.0048 | -0.0117 | 0.6650 |
| AgeAtNaturalMenopause | 0.0310 | 0.0123 | -0.0070 | 0.7930 | 0.0353 | 0.0124 | -0.0069 | 0.7555 |
| AgeFirstBirth | 0.0806 | 0.0099 | -0.0017 | 0.4304 | 0.0875 | 0.0095 | -0.0015 | 0.4250 |
| AgeofInitiation | 0.0275 | 0.0069 | -0.0037 | 0.7930 | 0.0558 | 0.0083 | -0.0028 | 0.5811 |
| AggressiveBehavior | -0.3866 | -0.0032 | -0.0369 | 0.1334 | -0.4273 | -0.0024 | -0.0373 | 0.1294 |
| Alcoholism | -0.0909 | -0.0012 | -0.0095 | 0.0888 | -0.1109 | -0.0011 | -0.0101 | 0.1046 |
| Alzheimers | 0.0673 | 0.0209 | -0.0103 | 0.7930 | -0.0784 | 0.0046 | -0.0156 | 0.5287 |
| AmyotrophicLateralSchlerosis | 0.0270 | 0.0162 | -0.0100 | 0.7930 | 0.0405 | 0.0176 | -0.0095 | 0.7516 |
| Anorexia | 0.0038 | 0.0109 | -0.0100 | 0.9318 | 0.0069 | 0.0111 | -0.0097 | 0.8963 |
| AntisocialBehavior | 0.1160 | 0.0218 | -0.0132 | 0.7930 | 0.0916 | 0.0196 | -0.0139 | 0.8625 |
| AnxietyDisorder | 0.0744 | 0.0200 | -0.0051 | 0.5464 | 0.0851 | 0.0194 | -0.0051 | 0.5141 |
| **Asthma** | 0.1464 | 0.0156 | 0.0035 | 0.0256 | 0.1653 | 0.0163 | 0.0032 | 0.0363 |
| AutismSpectrumDisorder | -0.0368 | 0.0087 | -0.0179 | 0.7930 | -0.0458 | 0.0088 | -0.0187 | 0.7379 |
| Baldness | 0.0834 | 0.0284 | -0.0005 | 0.2070 | 0.1047 | 0.0292 | 0.0006 | 0.1612 |
| BipolarDisorder | 0.0342 | 0.0177 | -0.0065 | 0.6577 | 0.0576 | 0.0200 | -0.0042 | 0.4927 |
| BirthWeight | 0.0378 | 0.0092 | -0.0025 | 0.5738 | 0.0463 | 0.0098 | -0.0027 | 0.5141 |
| BMI | -0.0618 | 0.0006 | -0.0133 | 0.2490 | -0.0504 | 0.0026 | -0.0115 | 0.4927 |
| BrainStructureHV | -0.1690 | -0.0011 | -0.0336 | 0.1882 | -0.2009 | -0.0011 | -0.0332 | 0.1503 |
| BrainStructureICV | -0.0557 | 0.0071 | -0.0227 | 0.6237 | -0.0723 | 0.0075 | -0.0243 | 0.5426 |
| **CeliacDisease** | -0.1562 | -0.0120 | -0.0499 | 0.0215 | -0.1742 | -0.0121 | -0.0490 | 0.0146 |
| Chronotype | -0.0470 | 0.0015 | -0.0125 | 0.3680 | -0.0639 | 0.0008 | -0.0137 | 0.2733 |
| CigarettesPerDay | 0.0255 | 0.0070 | -0.0034 | 0.7930 | 0.0175 | 0.0064 | -0.0042 | 0.8603 |
| CognitivePerformance | 0.0161 | 0.0103 | -0.0064 | 0.7930 | 0.0267 | 0.0111 | -0.0056 | 0.7405 |
| CoronaryArteryDisease | -0.0671 | 0.0032 | -0.0090 | 0.6577 | -0.0938 | 0.0026 | -0.0094 | 0.5141 |
| CrohnsDisease | 0.0690 | 0.0285 | 0.0002 | 0.1887 | 0.0801 | 0.0283 | -0.0001 | 0.1833 |
| Eczema | -0.1013 | 0.0034 | -0.0202 | 0.4304 | -0.1079 | 0.0043 | -0.0193 | 0.4927 |
| **Epilepsy** | -0.3831 | -0.0114 | -0.0338 | 0.0025 | -0.4167 | -0.0114 | -0.0332 | 0.0013 |
| FTD | 0.0845 | 0.0388 | -0.0217 | 0.7930 | 0.1297 | 0.0399 | -0.0183 | 0.7336 |
| **HDL** | 0.2898 | 0.0267 | 0.0113 | 0.0001 | 0.3575 | 0.0284 | 0.0130 | 8.1E-6 |
| Height | -0.0129 | 0.0084 | -0.0128 | 0.8208 | -0.0122 | 0.0086 | -0.0122 | 0.8625 |
| InflammatoryBowelDisease | 0.0670 | 0.0238 | 0.0004 | 0.1887 | 0.0825 | 0.0242 | 0.0011 | 0.1402 |
| Insomnia | 0.0819 | 0.0121 | -0.0023 | 0.4367 | 0.1094 | 0.0127 | -0.0014 | 0.3546 |
| InternalizingProblems | -0.0487 | 0.0251 | -0.0343 | 0.8538 | -0.0506 | 0.0268 | -0.0354 | 0.8627 |
| IschemicStroke | -0.0210 | 0.0119 | -0.0150 | 0.8647 | -0.0278 | 0.0113 | -0.0147 | 0.8627 |
| LDL | 0.1659 | 0.0202 | 0.0016 | 0.1334 | 0.1613 | 0.0170 | 0.0014 | 0.1205 |
| Loneliness | -0.0199 | 0.0034 | -0.0057 | 0.7930 | -0.0287 | 0.0034 | -0.0062 | 0.7516 |
| MajorDepressiveDisorder | 0.0222 | 0.0061 | -0.0032 | 0.7930 | 0.0277 | 0.0064 | -0.0033 | 0.7500 |
| **MultipleSclerosis** | -0.1855 | -0.0110 | -0.0705 | 0.0649 | -0.2268 | -0.0180 | -0.0639 | 0.0073 |
| Neuroticism | 0.0499 | 0.0109 | -0.0027 | 0.5464 | 0.0642 | 0.0114 | -0.0026 | 0.4927 |
| NumberChildrenEverBorn | -0.0205 | 0.0034 | -0.0048 | 0.8436 | -0.0317 | 0.0031 | -0.0051 | 0.8125 |
| NumberSexualPartners | 0.0344 | 0.0088 | -0.0025 | 0.5745 | 0.0454 | 0.0097 | -0.0026 | 0.5141 |
| OCD | -0.0081 | 0.0230 | -0.0255 | 0.9318 | -0.0127 | 0.0227 | -0.0259 | 0.8963 |
| PrimaryBiliaryCirrhosis | 0.0835 | 0.0420 | -0.0007 | 0.2070 | 0.1056 | 0.0429 | 0.0035 | 0.1205 |
| PrimarySclerosingCholangitis | -0.0241 | 0.0471 | -0.0615 | 0.8551 | -0.0350 | 0.0525 | -0.0712 | 0.8625 |
| RestingHeartRatepvRSA | -0.0500 | 0.0092 | -0.0174 | 0.7930 | -0.0603 | 0.0087 | -0.0177 | 0.7379 |
| RestingHeartRateRMSSD | -0.0525 | 0.0084 | -0.0165 | 0.7930 | -0.0513 | 0.0094 | -0.0166 | 0.7555 |
| RestingHeartRateSDNN | 0.0290 | 0.0155 | -0.0115 | 0.8538 | 0.0213 | 0.0150 | -0.0123 | 0.8944 |
| RheumatoidArthritis | -0.1941 | -0.0004 | -0.0825 | 0.1887 | -0.2336 | -0.0088 | -0.0768 | 0.1046 |
| RiskyBehavior | 0.0301 | 0.0078 | -0.0029 | 0.6577 | 0.0367 | 0.0081 | -0.0030 | 0.6182 |
| Schizophrenia | -0.0251 | 0.0131 | -0.0226 | 0.7930 | -0.0303 | 0.0092 | -0.0189 | 0.7379 |
| SleepDuration | -0.0768 | 0.0022 | -0.0118 | 0.4367 | -0.0797 | 0.0023 | -0.0118 | 0.4927 |
| SmokingCessation | 0.1042 | 0.0097 | 0.0001 | 0.1887 | 0.1222 | 0.0099 | 0.0000 | 0.1833 |
| SmokingInitiation | 0.0082 | 0.0049 | -0.0038 | 0.8551 | 0.0069 | 0.0048 | -0.0040 | 0.8944 |
| SubjectiveWellBeing | -0.0215 | 0.0034 | -0.0050 | 0.8208 | -0.0217 | 0.0036 | -0.0051 | 0.8625 |
| **SuicideAttempt** | 0.6772 | 0.0173 | 0.0054 | 0.0043 | 0.6773 | 0.0169 | 0.0059 | 0.0013 |
| SystemicLupusErythematosus | 0.0223 | 0.0335 | -0.0222 | 0.8208 | 0.0209 | 0.0327 | -0.0238 | 0.8625 |
| **TotalCholesterol** | 0.1852 | 0.0218 | 0.0047 | 0.0256 | 0.1732 | 0.0188 | 0.0030 | 0.0623 |
| TouretteSyndrome | -0.0822 | 0.0050 | -0.0333 | 0.4218 | -0.1019 | 0.0047 | -0.0340 | 0.3932 |
| Triglycerides | 0.1187 | 0.0156 | 0.0008 | 0.1769 | 0.1035 | 0.0137 | -0.0011 | 0.3007 |
| Type2Diabetes | -0.0066 | 0.0011 | -0.0021 | 0.7930 | -0.0133 | 0.0005 | -0.0023 | 0.5052 |
| UlcerativeColitis | -0.0205 | 0.0007 | -0.0078 | 0.3259 | -0.0275 | -0.0005 | -0.0076 | 0.1294 |
| **Table S19: Genetic covariance analysis between 63 complex human traits and amyloid PET GWAS for Non-Hispanic Whites (NHW) ancestry (N = 11,556).** Traits having significant association (FDR < 0.05) with Amyloid PET are represented in bold. Corr_corrected represents the genetic correlation that is corrected for sample overlap. ci_upper and ci_lower represent the 90% confidence interval. | | | | | | | | |

| 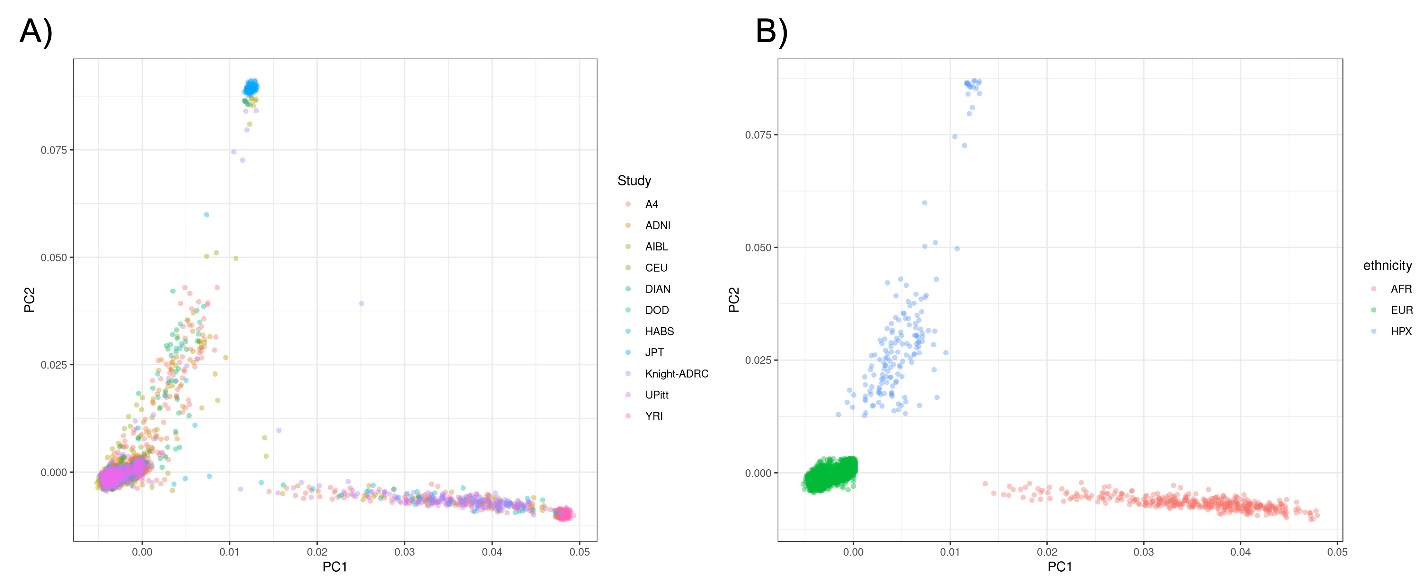 |
| --- |
| **Fig. S1: First two principal components of the genetic population structure across 8 cohorts together with the reference HapMap data**. A) First two principal components of the genetic population structure across 8 cohorts (A4, ADNI, AIBL, DIAN, DOD, HABS, Knight-ADRC, and UPitt) analyzed in this study, together with reference HapMap data (CEU, JPT, and YRI). B) First two principal components of the genetic population structure across American African (AFR), non-Hispanic white (EUR), and Asian (HPX) ethnicities. Abbreviations: PC, principal component. |

| 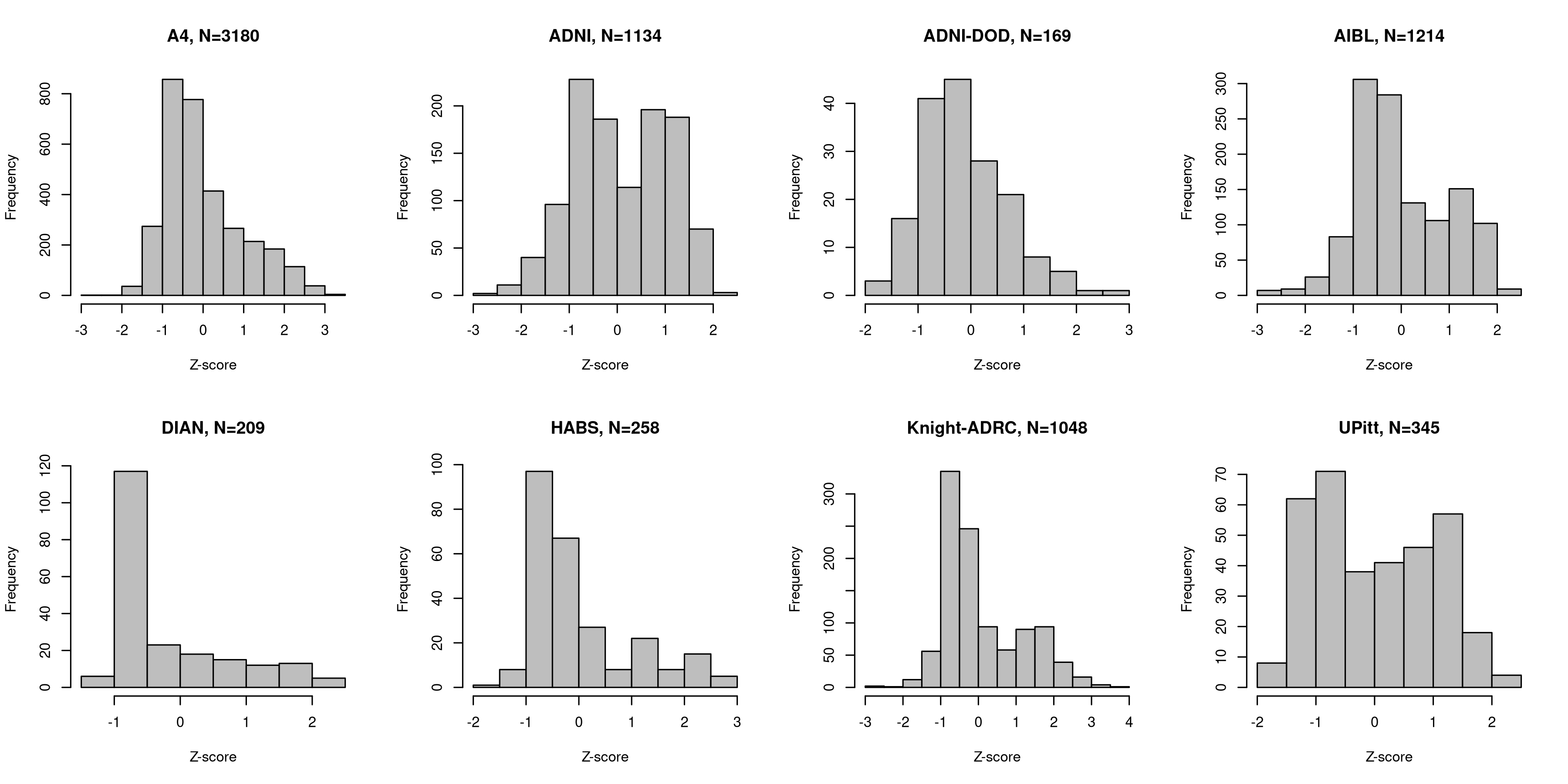 |
| --- |
| **Fig. S2: Distribution of harmonized amyloid PET endophenotype (z-score) across 8 cohorts**. The distribution of harmonized and standardized amyloid PET endophenptype across 8 cohorts (A4, ADNI, ADNI-DOD, AIBL, DIAN, HABS, Knight-ADRC, and UPitt) analyzed in this study. |

| 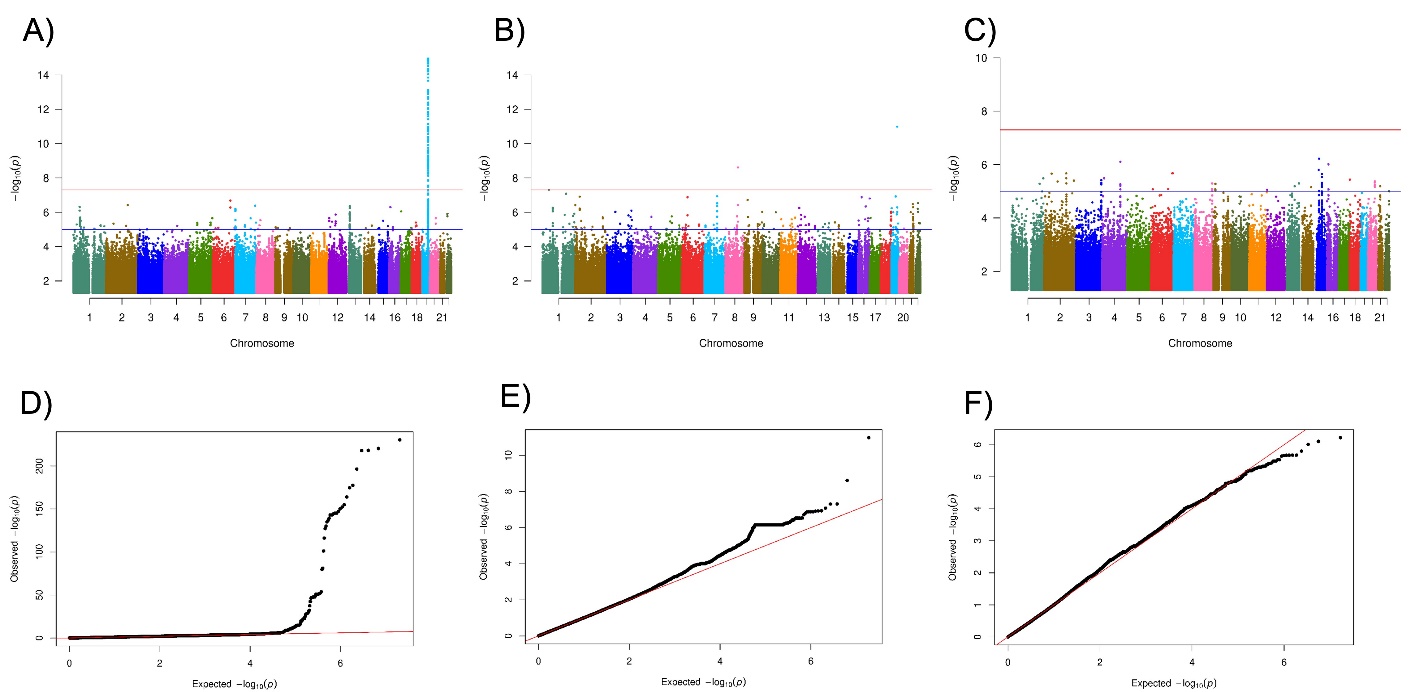 |
| --- |
| **Fig. S3:** **Manhattan and Quantile–quantile for race-specific join analysis in A) non-Hispanic white (NHW), B) American African (AFR), and C) Asian (ASN) populations.** Manhattan plot showing the P-values in the race-specific join analysis for EUR (n = 7,036), AFR (n = 359), and ASN (n = 162) populations across 8 cohorts having phenotypic and genotypic data. The blue and red lines represent the suggestive (P = 1 × 10^−5^) and genome-wide significance thresholds (P = 5 × 10^−8^). Variants with a P value below 1 × 10^−15^ are not shown. The lower panel shows the Quantile–quantile plot for the race-specific joint analysis for NHW (D), AFR (E), and ASN (F) populations across 8 cohorts. The observed genomic control value (λ) was 1.009, 1.009, and 1 for NHW, AFR, and ASN populations, respectively. |

| 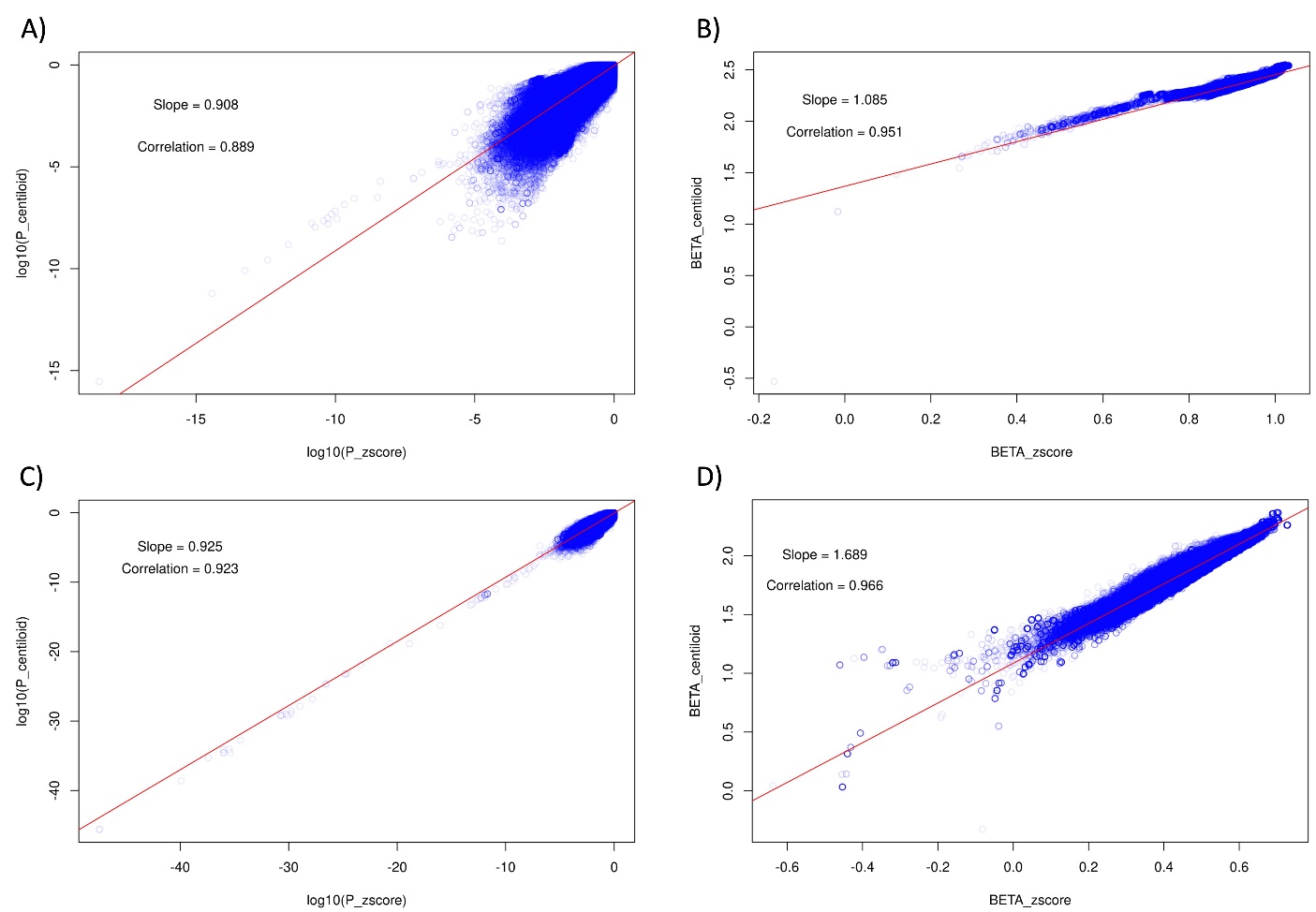 |
| --- |
| **Fig. S4:** **Correlation between P-value and BETA for GWAS using Z-score and raw Centiloid measure as the phenotype from Knight-ADRC and ADNI cohorts.** Correlation between the P-value (A) and effect size (B) from the GWAS analyses using Z-score (x-axis) and Centiloid measure (y-axis) as phenotype in Knight-ADRC cohort. Correlation between the P-value (C) and effect size (D) from the GWAS analyses using Z-score (x-axis) and Centiloid measure (y-axis) as phenotype in ADNI cohort. |

| 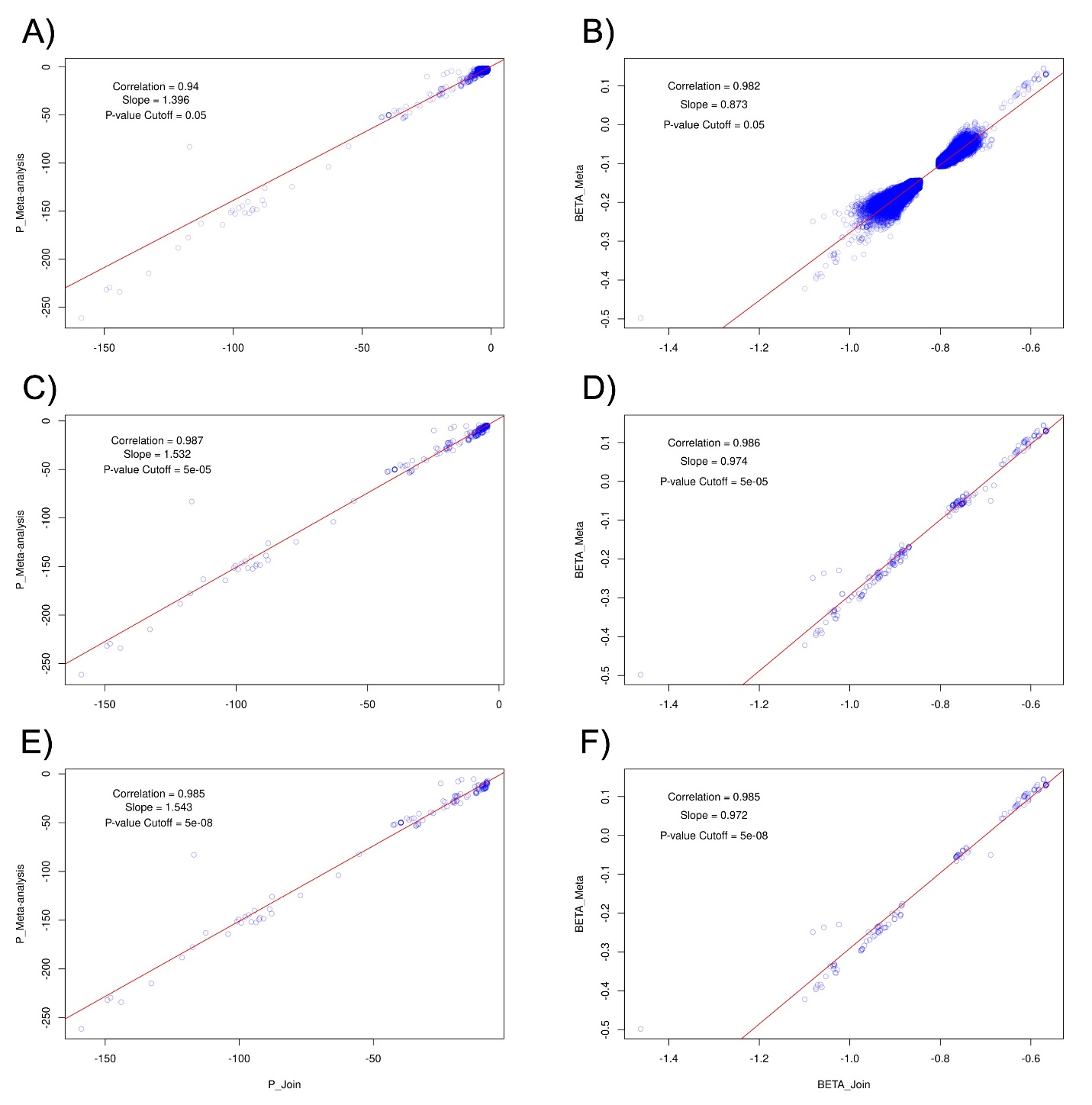 |
| --- |
| **Fig. S5: Correlation between P-values and BETA from meta- and joint-analyses of 8 cohorts.** For all the cohorts having genotype and phenotype data (N=8), we performed the GWAS using log10 of raw phenotype values (e.g. PIB, Centiloid, or SUVR) for each cohort individually and then perform the SE-based meta-analysis using METAL. We also performed the joint analysis of all 8 cohorts using z-score as the quantitative phenotype. Panel A) and B) shows the correlation between the P-values and BETA, respectively, for both these analyses. Panel C) and D) also represent the same correlation plots for SNPs passing the suggestive significance threshold (P = 1 × 10^−5^). Panel E) and F) shows the correlation of P-value and BETA for SNPs passing the genome-wide significance thresholds (P = 5 × 10^−8^). |

| 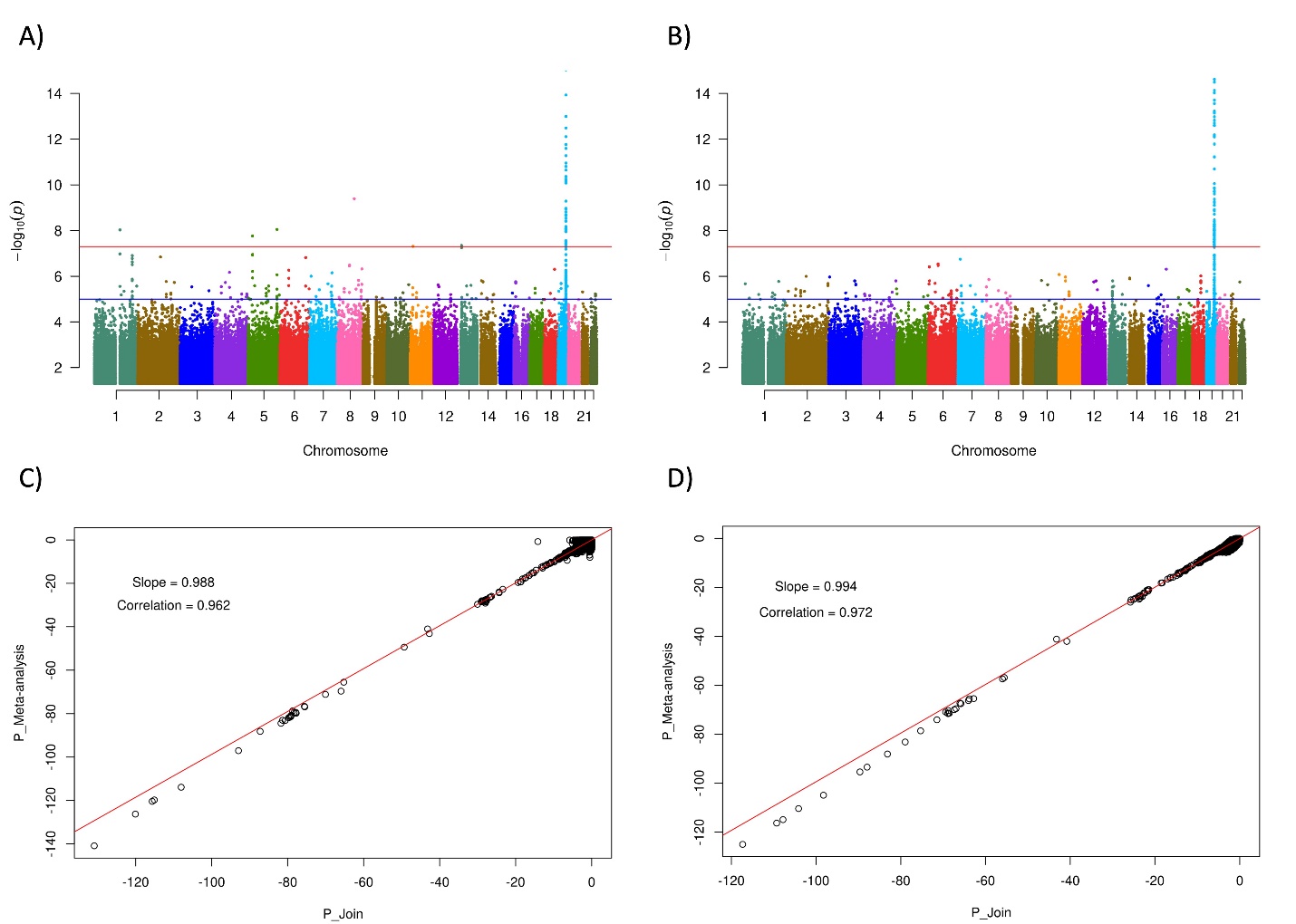 |
| --- |
| **Fig. S6:** **Manhattan (A,B) and correlation plot (C,D) for multi-ethnic sex-stratified meta-analyses.** Manhattan plot showing the P-values from the association analysis conducted for 4,096 female (A) and 3,461 male (B) participants across 8 cohorts having phenotypic and genotypic data. The blue and red lines represent the suggestive (P = 1 × 10^−5^) and genome-wide significance thresholds (P = 5 × 10^−8^). Variants with a P value below 1 × 10^−15^ are not shown. The lower panel shows the correlation plot for the female (C) and male (D) participants among p-values from the meta- and joint-analyses. |

| 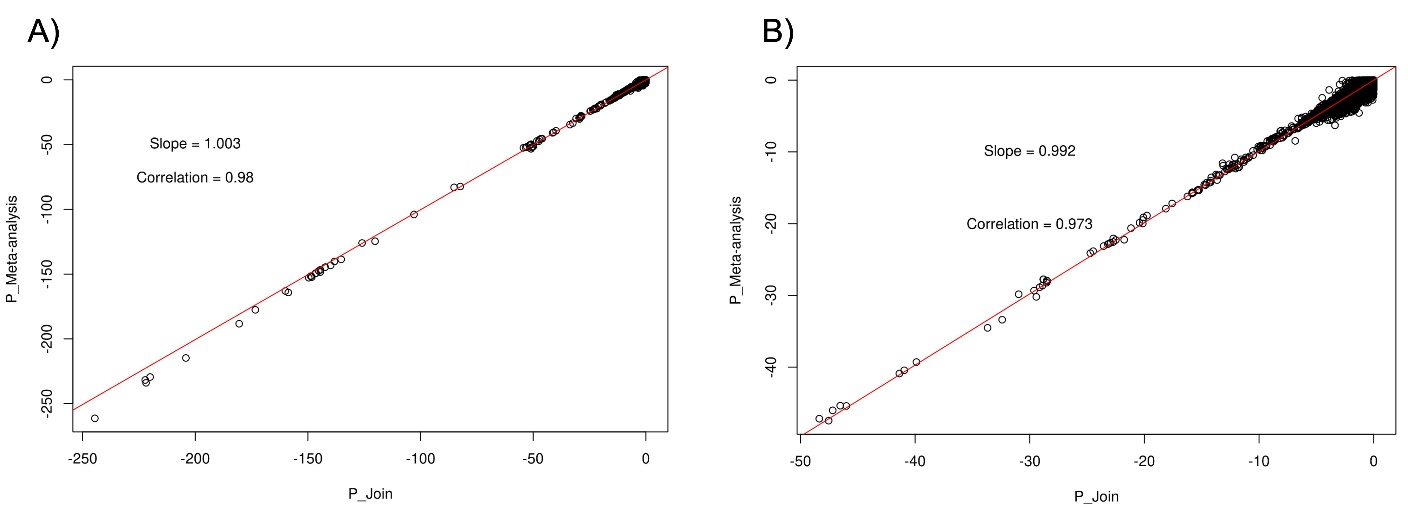 |
| --- |
| **Fig. S7: Correlation between P-values from race-specific meta- and joint-analyses.** A) This plot shows the correlation between P-values from the standard-error (StdErr)-based meta-analysis by METAL software (Y-axis) and Join analysis (X-axis) from the race-specific GWAS analysis. B) Same correlation plot as shown in panel A but the P-values were restricted to P-values greater than 2 × 10^−50^. |

| 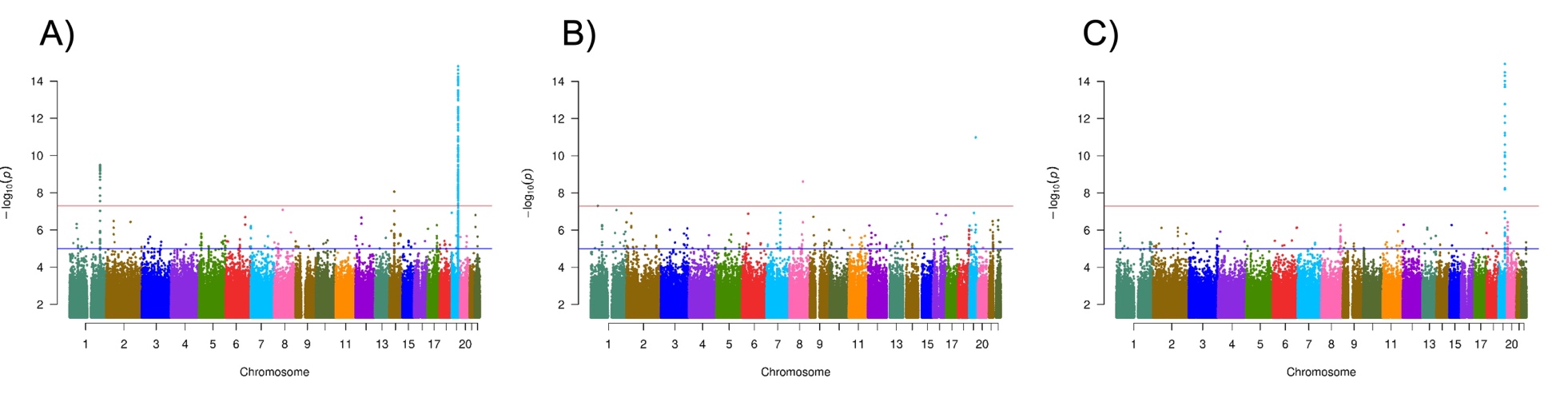 |
| --- |
| **Fig. S8:** **Manhattan plot for race-specific meta-analysis in A) non-Hispanic white (NHW), B) American African (AFR), and C) Asian (ASN) populations.** Manhattan plot showing the P-values in the race-specific meta-analysis for NHW (n = 11,556), AFR (n = 359), and ASN (n = 1,494) populations across multiple cohorts. The blue and red lines represent the suggestive (P = 1 × 10^−5^) and genome-wide significance thresholds (P = 5 × 10^−8^). Variants with a P value below 1 × 10^−15^ are not shown. |

| 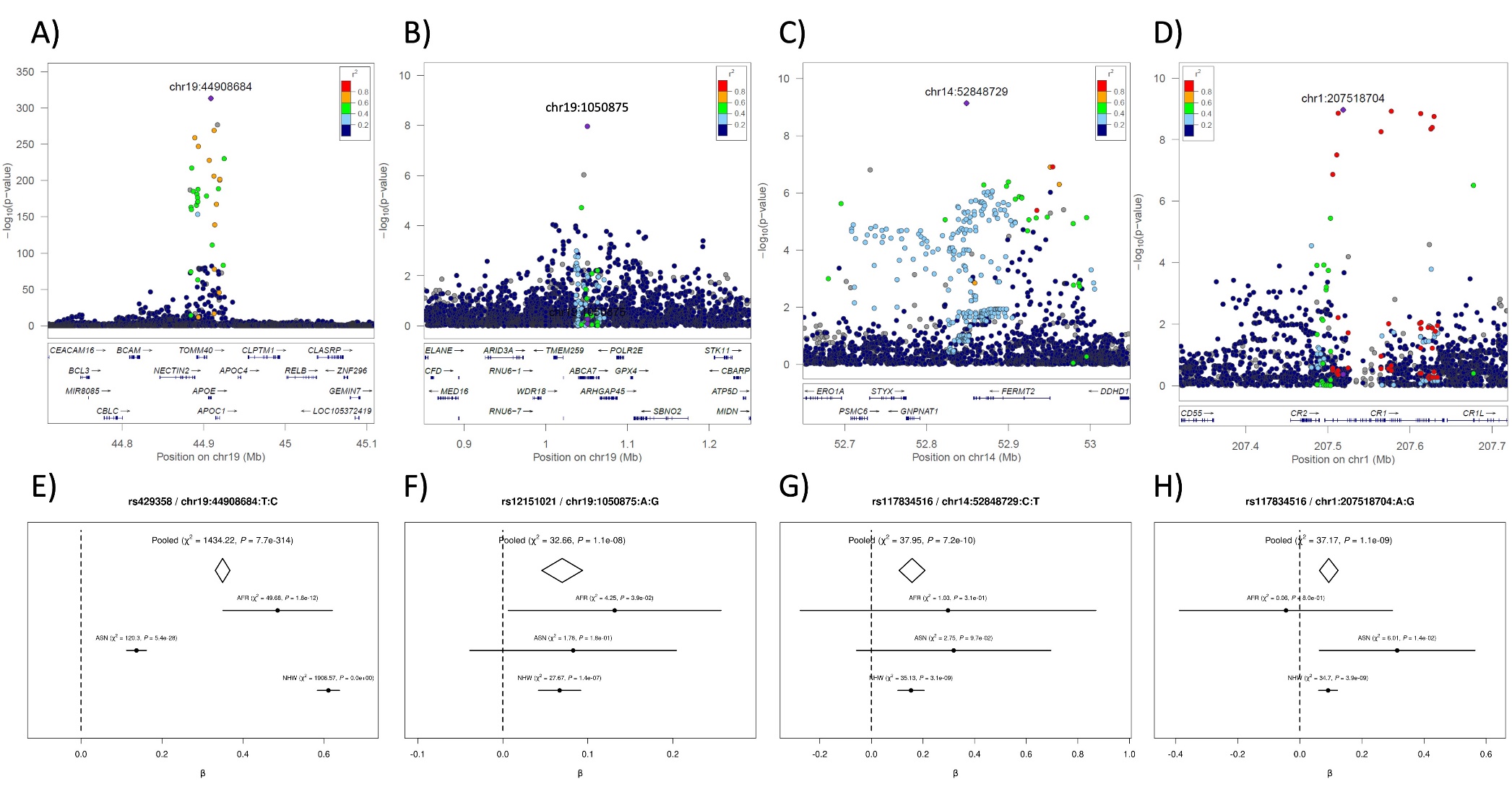 |
| --- |
| **Fig. S9:** **Regional locus zoom and forest plots of genome-wide significant SNPs from the multi-ethnic GWAS.** Regional locus zoom plots of genome-wide significant SNPs from multi-ethnic GWAS (A) rs429358 / chr19:44908684:T:C from chr19q.13.32 (B) rs12151021 / chr19:1050875:A:G from chr19p.13.3 (C) rs117834516 / chr14:52848729:C:T from chr14q.22.1 and (D) rs6656401 / chr1:207518704:A:G from chr1q.32.2. The chromosomal position is shown on the x –axis and the y-axis shows the significance of the associations. The purple diamond shows the P-value for the most significant SNP in the meta-analysis in that region. The circles show the P-values for all other SNPs in that particular genomic region and are color coded according to the level of LD with top hit in the 1000 Genome Project EUR population. Panel E-H shows the forest plot for the top SNPs demonstrating the direction of effect and its standard error (SE) across NHW, AFR, and ASN ethnicities. |

| 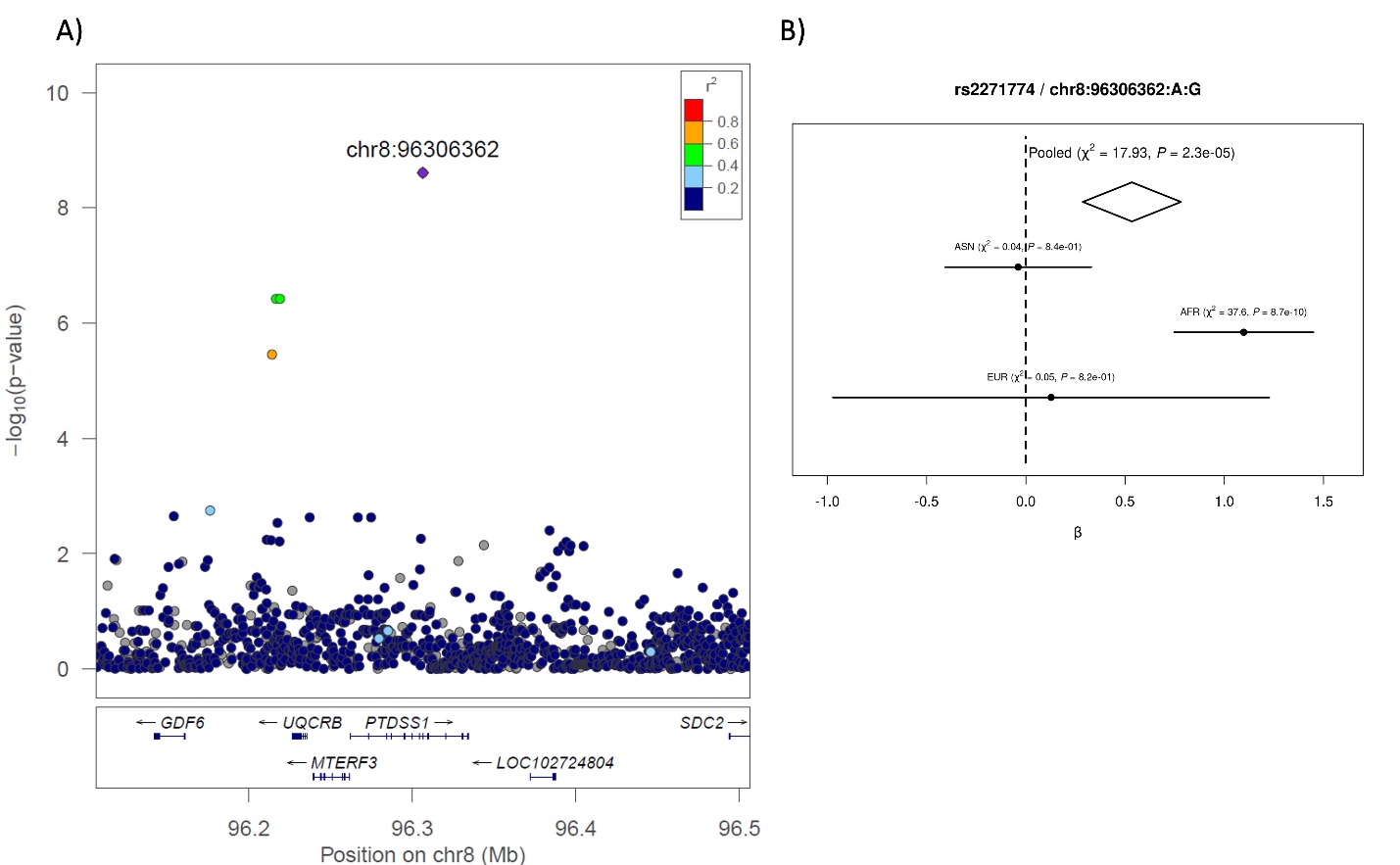 |
| --- |
| **Fig. S10:** **Chr8q.22.1 (rs2271774) is associated with amyloid imaging in the AFR population.** Regional locus zoom (A) for the AFR population and Forest plot (B) of genome-wide significant hit from chromosome 8. A) The relative location of genes and the direction of transcription are shown in the lower portion of the locus zoom plot for rs2271774 (chr8:96306362:A:G) genome-wide significant hit. The chromosomal position is shown on the x –axis and the y-axis shows the significance of the associations. The purple diamond shows the P-value for rs2271774 that is the most significant SNP in the meta-analysis in that region. The circles show the P-values for all other SNPs in that particular genomic region and are color coded according to the level of LD with rs2271774 in the 1000 Genome Project EUR population. B) Forest plot for the top SNP on chromosome 8 is presented demonstrating consistent direction across AFR and NHW (EUR) ethnicities. |

| 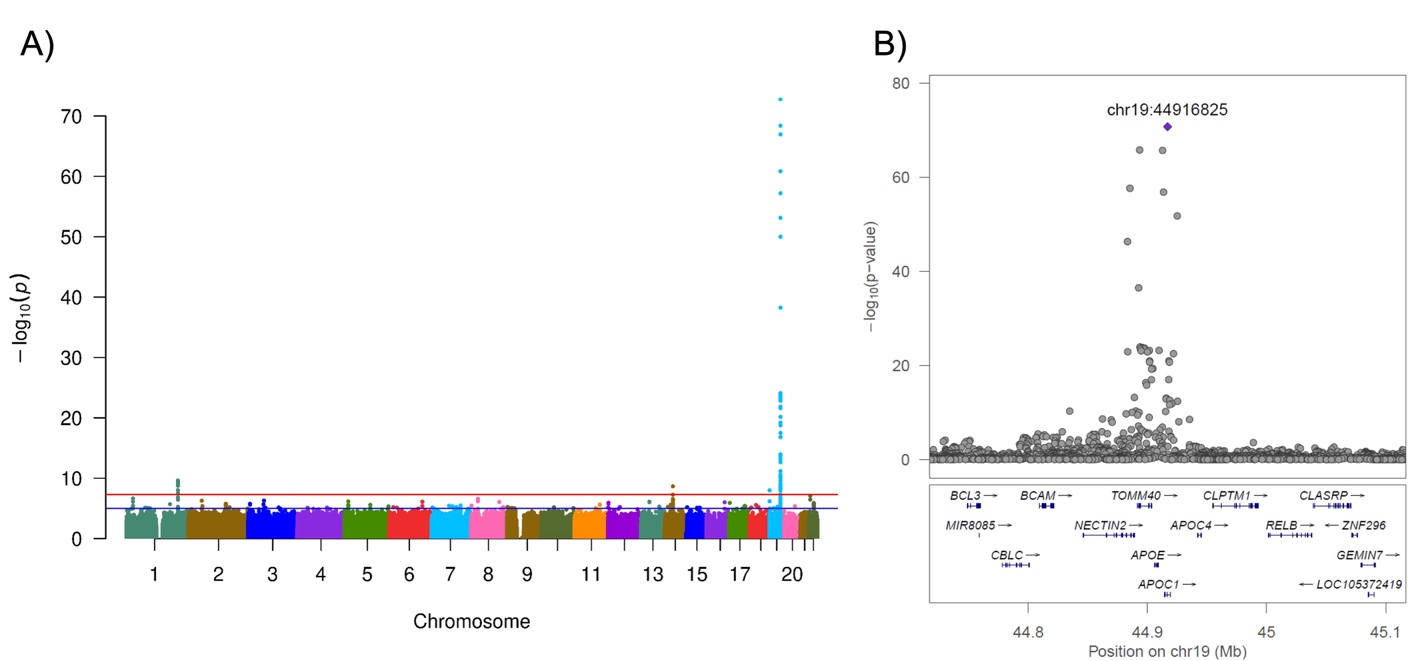 |
| --- |
| **Fig. S11: Manhattan (A), Quantile–quantile (B), and Regional locus zoom plots for Multi-ethnic *APOE*-conditional analysis (N=13,409).** A) Manhattan plot showing the P-values in the multi-ethnic conditional analysis on the top *APOE ɛ4* (rs429358/chr19:44908684:T:C, β =  0.35, P = 7.7 × 10^−314^) and *APOE ɛ2* (rs7412/ chr19:44908822:C:T, β =  -0.20, P = 1.4 × 10^−27^) signals from the multi-ethnic meta-analysis of 14 cohorts. The blue and red lines represent the suggestive (P = 1 × 10^−5^) and genome-wide significance thresholds (P = 5 × 10^−8^). B) The relative location of genes and the direction of transcription are shown in the lower portion of the locus zoom plot for rs73052335 (chr19:44916825:A:C) genome-wide significant hit. The chromosomal position is shown on the x–axis and the y-axis shows the significance of the associations. The purple diamond shows the P-value for rs73052335 that is the most significant SNP in that region. The circles show the P-values for all other SNPs in that particular genomic region and are color coded according to the level of LD with rs73052335 in the 1000 Genome Project EUR population. |

| 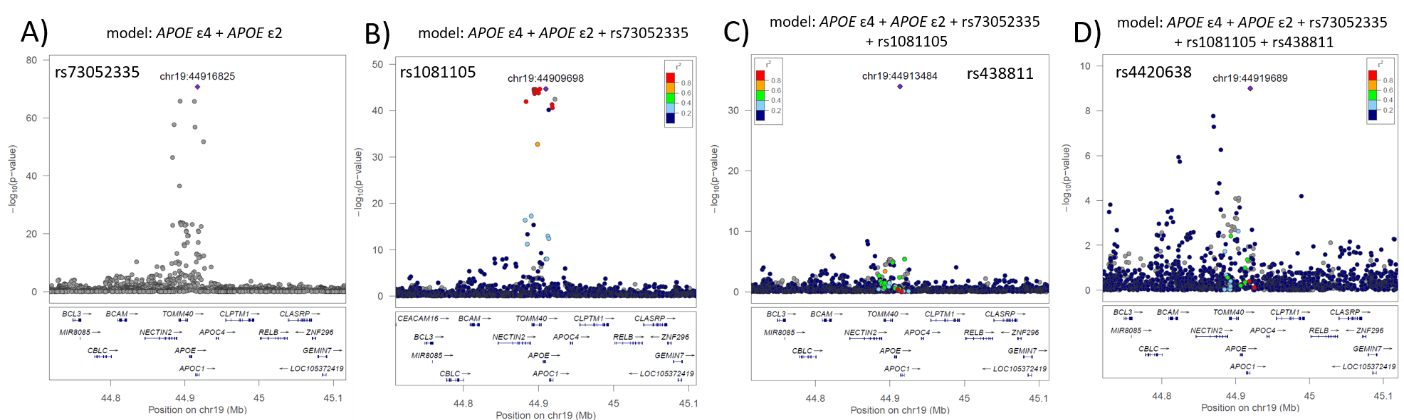 |
| --- |
| **Fig. S12:** **LocusZoom plot of independent signals from the conditional analyses on** ***APOE* ɛ4 and *APOE* ɛ2.** The locus zoom plots for A) rs73052335/chr19:44916825:A:C, B) rs1081105/chr19:44909698:A:C, C) rs438811/ chr19:44913484:C:T, and D) rs4420638/ chr19:44919689:A:G from the *APOE* conditional analyses on *APOE* ɛ4 (rs429358) + *APOE* ɛ2 (rs7412), *APOE* ɛ4 + *APOE* ɛ2 + rs73052335, *APOE* ɛ4 + *APOE* ɛ2 + rs73052335 + rs1081105, *APOE* ɛ4 + *APOE* ɛ2 + rs73052335 + rs1081105 + rs438811, respectively. The relative location of genes and the direction of transcription are shown in the lower portion of the locus zoom plot. The chromosomal position is shown on the x–axis and the y-axis shows the significance of the associations. The purple diamond shows the P-value for the SNP that is the most significant in that region. The circles show the P-values for all other SNPs in that particular genomic region and are color coded according to the level of LD with the sentinel SNP in the 1000 Genome Project EUR population |

| 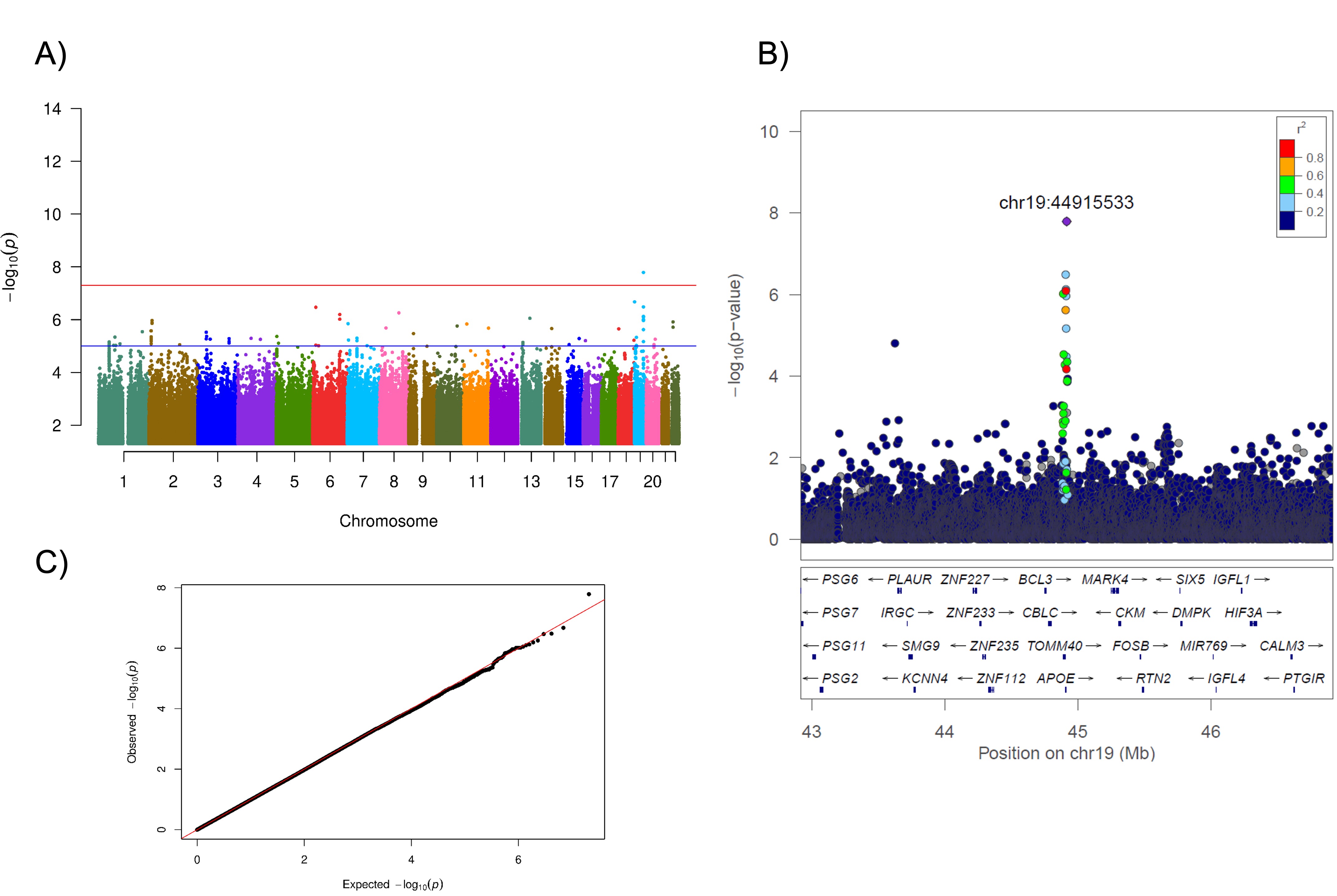 |
| --- |
| **Fig. S13: Manhattan (A), Regional locus zoom (B) and Quantile–quantile (C) plots for multi-ethnic joint analysis adjusted for the *APOE* ɛ4 allele genotype (N=7,557).** A) Manhattan plot showing the P-values in the multi-ethnic GWAS where *APOE* genotype (e.g. 22, 23, 34, 44) was used as a covariate in the model. The blue and red lines represent the suggestive (P = 1 × 10^−5^) and genome-wide significance thresholds (P = 5 × 10^−8^). B) The relative location of genes and the direction of transcription are shown in the lower portion of the locus zoom plot for rs5117 genome-wide significant hit. The chromosomal position is shown on the x–axis and the y-axis shows the significance of the associations. The purple diamond shows the P-value for rs5117 that is the most significant SNP in that region. The circles show the P-values for all other SNPs in that particular genomic region and are color coded according to the level of LD with rs5117 in the 1000 Genome Project EUR population. C) Quantile–quantile plot for the multi-ethnic joint analysis adjusted for the *APOE* ɛ4 allele genotype. The observed genomic control value (λ) was 1. |

| 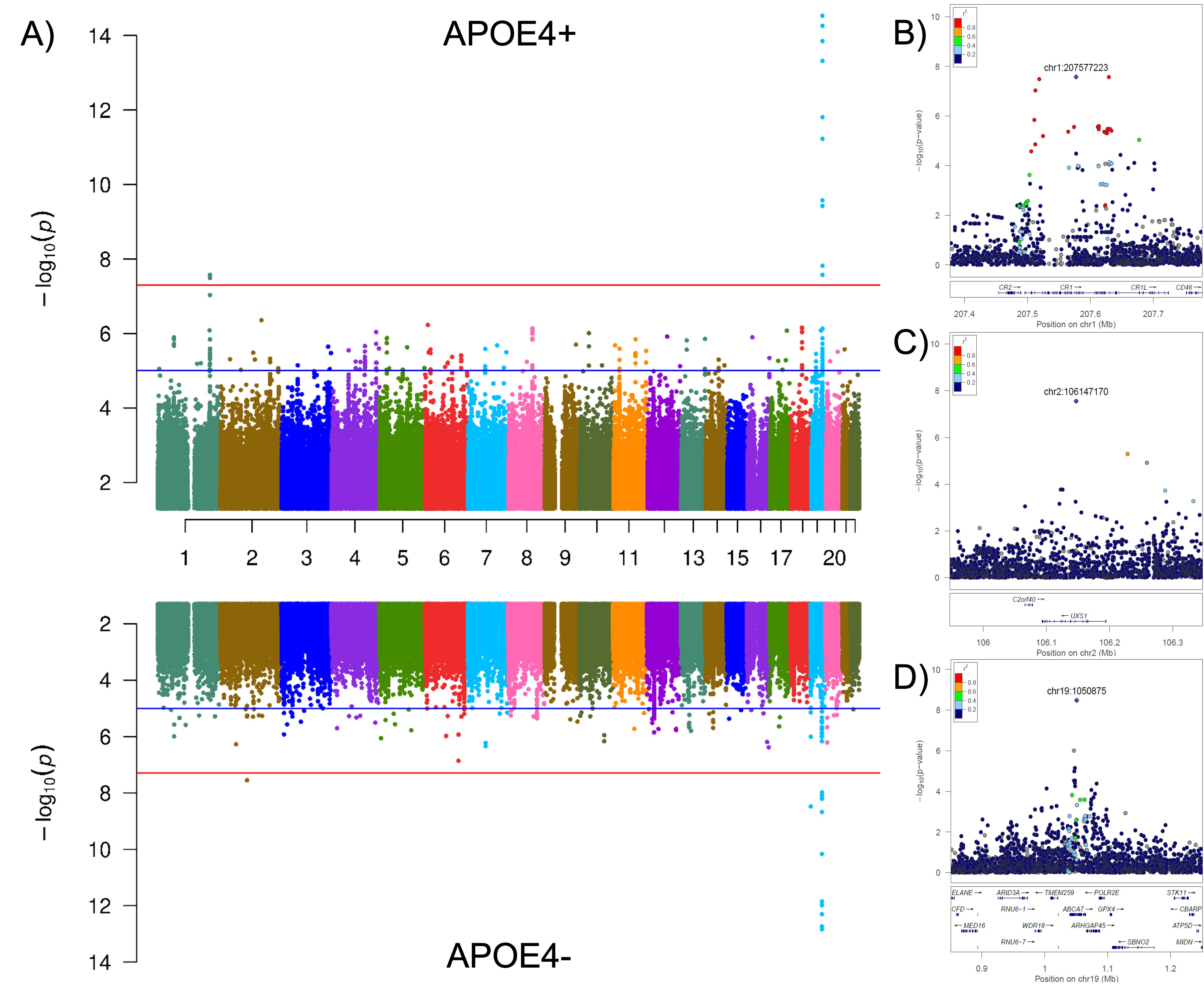 |
| --- |
| **Fig. S14:** **Manhattan (A) and Locus Zoom plots (B,C,D) for multi-ethnic** ***APOE* ɛ4-stratified analysis.** Manhattan plot showing the P-values in the 3,844 *APOE* ɛ4+ (Panel A top figure) and 5,931 *APOE* ɛ4- (Panel A bottom figure) participants across 8 cohorts having phenotypic and genotypic data as well as external cohort MISSION-AD. The blue and red lines represent the suggestive (P = 1 × 10^−5^) and genome-wide significance thresholds (P = 5 × 10^−8^). Variants with a P value below 1 × 10^−15^ are not shown. The panels on the right show the locus zoom plots for the novel signal on B) chr 1 from the *APOE* ɛ4+ GWAS as well as hits on the C) chr2q.12.2 (rs567226423 / chr2:106147170:A:G) and D) chr19p.13.3 (rs12151021 / chr19:1050875:A:G) from the *APOE* ɛ4- GWAS. |

| 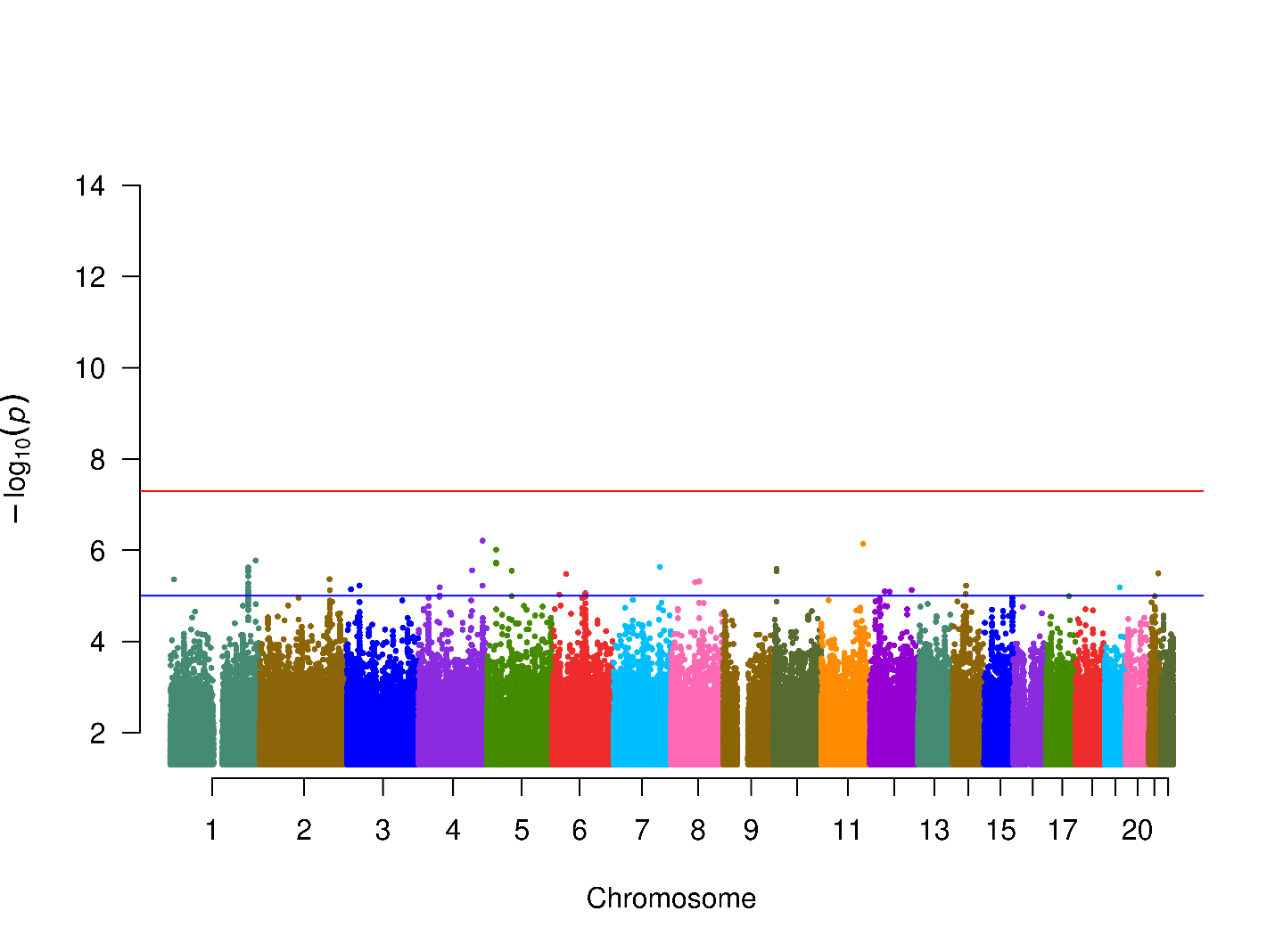 |
| --- |
| **Fig. S15: Manhattan plot for multi-ethnic sex-AD interaction analysis.** Manhattan plot showing the P-values from the sex-AD interaction analysis conducted for 7,557 participants across 8 cohorts having phenotypic and genotypic data. The blue and red lines represent the suggestive (P = 1 × 10^−5^) and genome-wide significance thresholds (P = 5 × 10^−8^). |

| 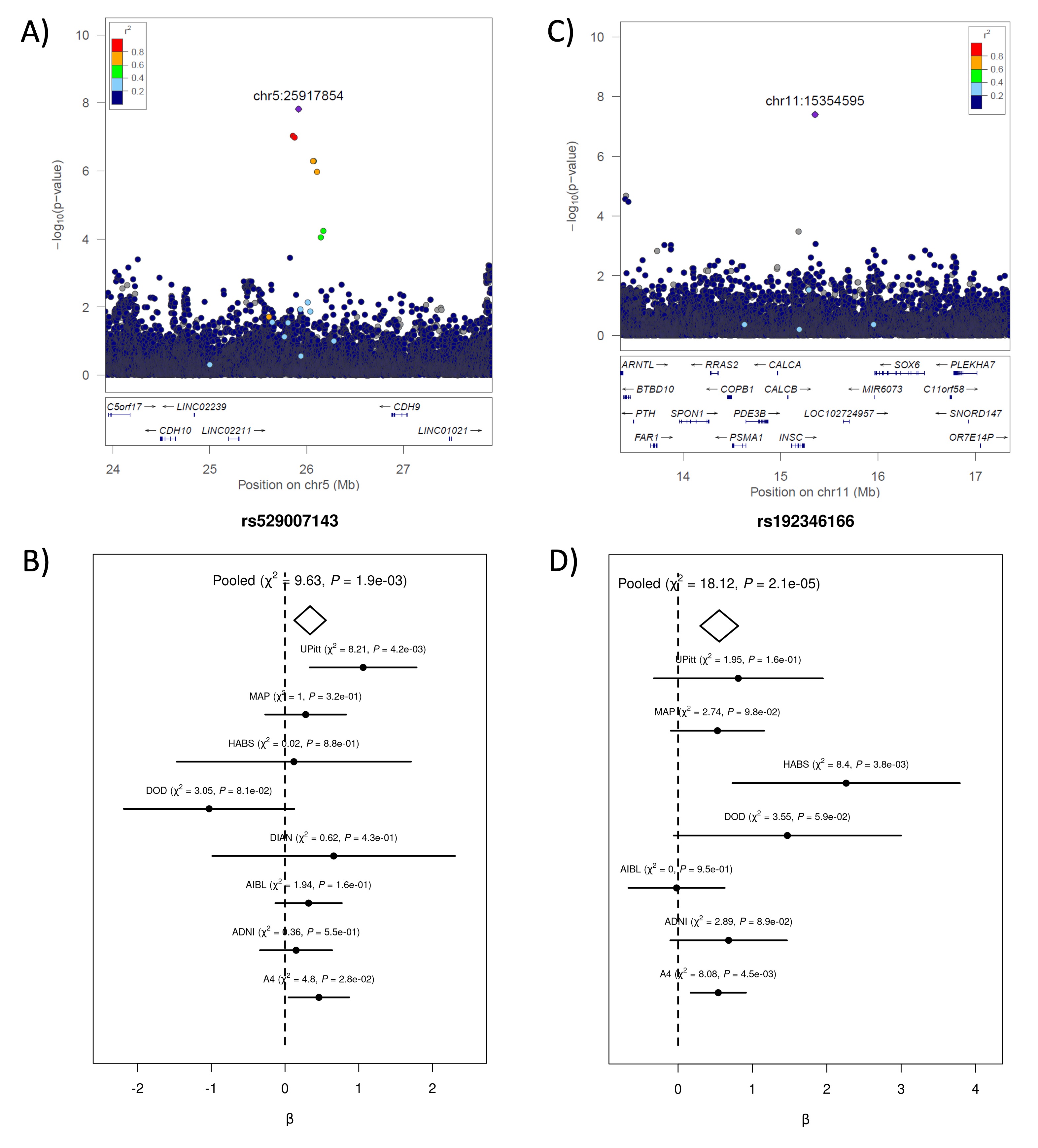 |
| --- |
| **Fig. S16: Regional locus zoom and forest plots for genome-wide significant hits from sex-stratified (female) joint analysis (N=9,820).** The locus zoom plots for A) chr5p.14.1 signal (chr5:25917854:G:A/rs529007143), and C) chr11p.15.2 (chr11:15354595:A:G/rs192346166) from the multi-ethnic sex-stratified (female) GWAS. The relative location of genes and the direction of transcription are shown in the lower portion of the locus zoom plot for rs529007143, and rs529007143 genome-wide significant hit from sex-stratified (female) GWAS. The chromosomal position is shown on the x–axis and the y-axis shows the significance of the associations. The purple diamond shows the P-value for the SNP that is the most significant in that region. The circles show the P-values for all other SNPs in that particular genomic region and are color coded according to the level of LD with the sentinel SNP in the 1000 Genome Project EUR population. Forest plot showing the estimate and standard error (SE) from each cohorts for the same SNPs are shown in the lower panel (B and D for rs529007143 and rs529007143, respectively). |

| 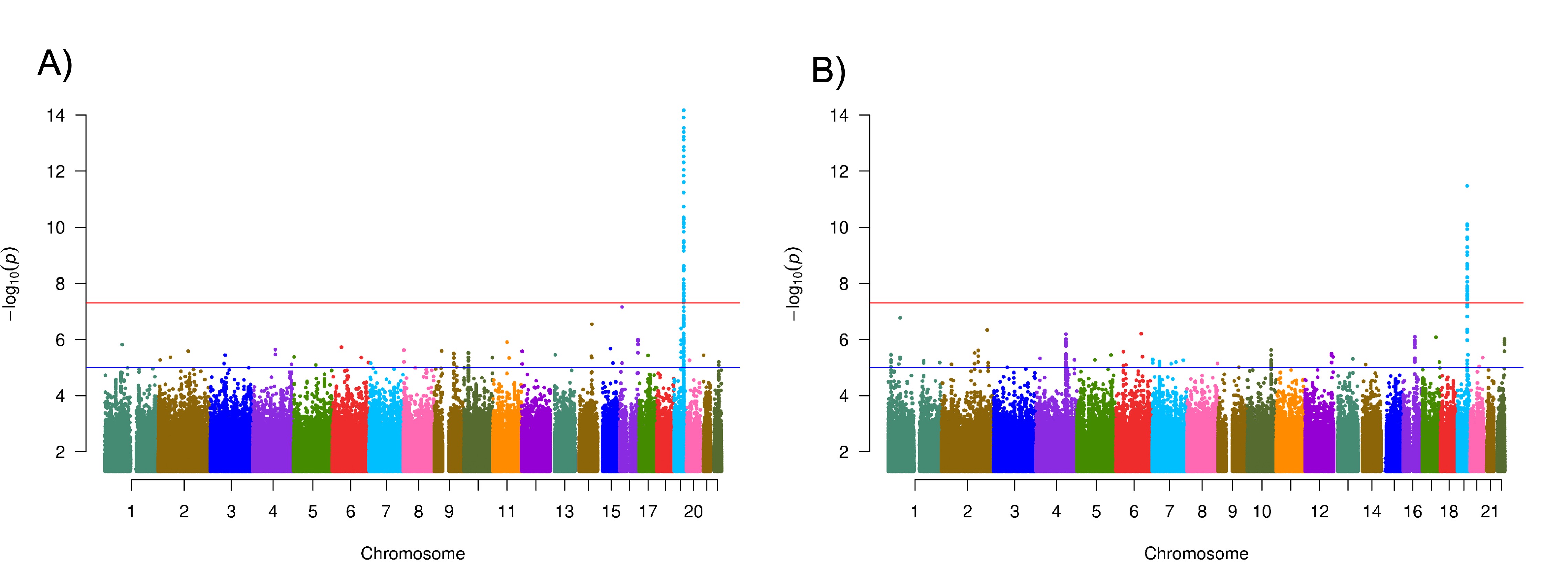 |
| --- |
| **Fig. S17:** **Manhattan plot for multi-ethnic case-control-stratified meta-analyses.** Manhattan plot showing the P-values from the association analysis conducted for the A) 5,846 controls (CO) and B) 1,138 AD participants across 8 cohorts having phenotypic and genotypic data. The blue and red lines represent the suggestive (P = 1 × 10^−5^) and genome-wide significance thresholds (P = 5 × 10^−8^). Variants with a P value below 1 × 10^−15^ are not shown. |

| 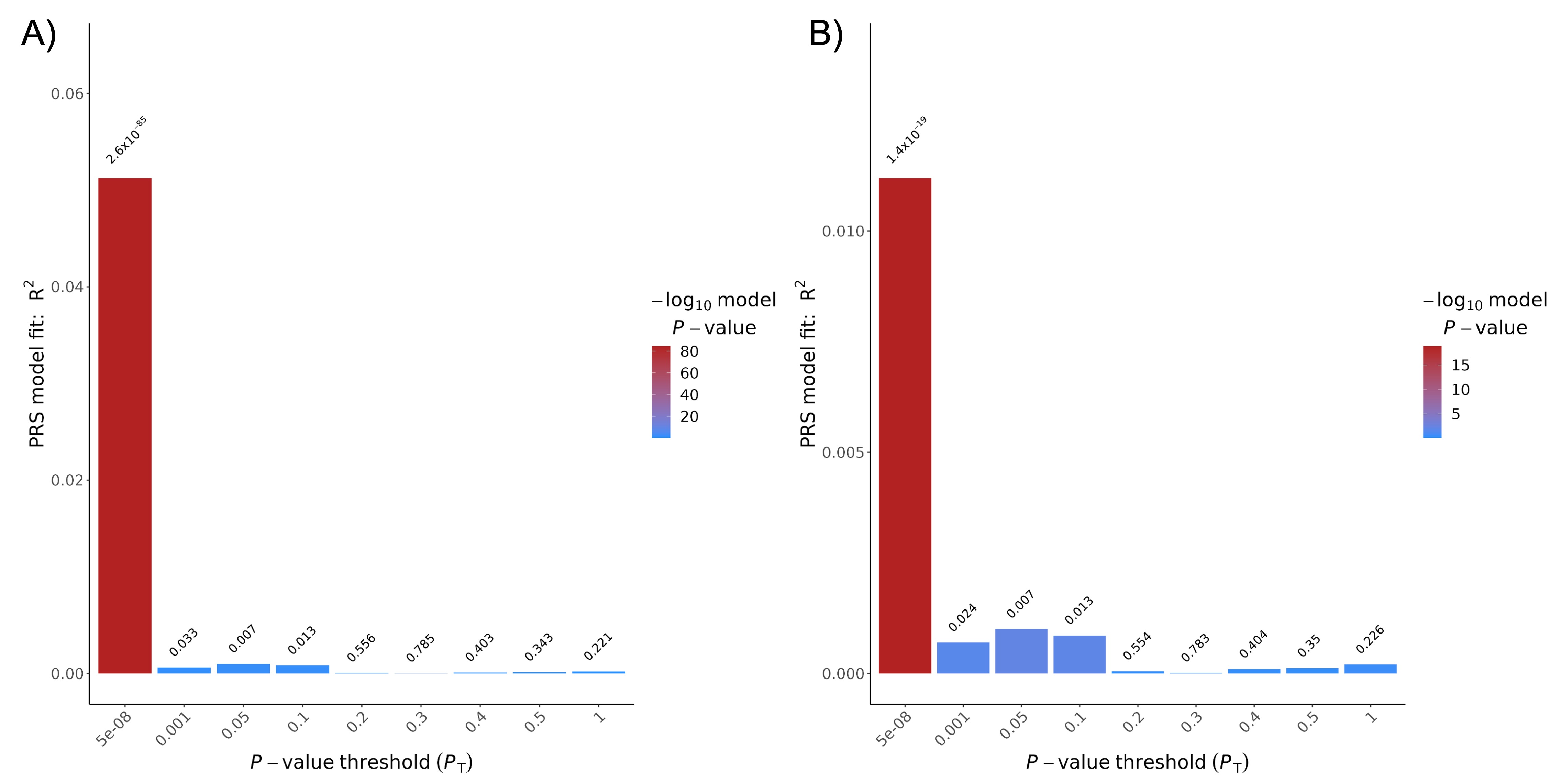 |
| --- |
| **Fig. S18:** **Polygenic risk score (PRS) analysis with (A) and without (B) *APOE* region.** Bar plot showing different P-value thresholds (P_T_) used for calculating PRS on the x-axis and PRS model fit (R2) on the y-axis. PRS calculation was performed on 9 different thresholds (P_T_) and the most predictive (precise) threshold was 5×10^-08^ in both cases. |

| 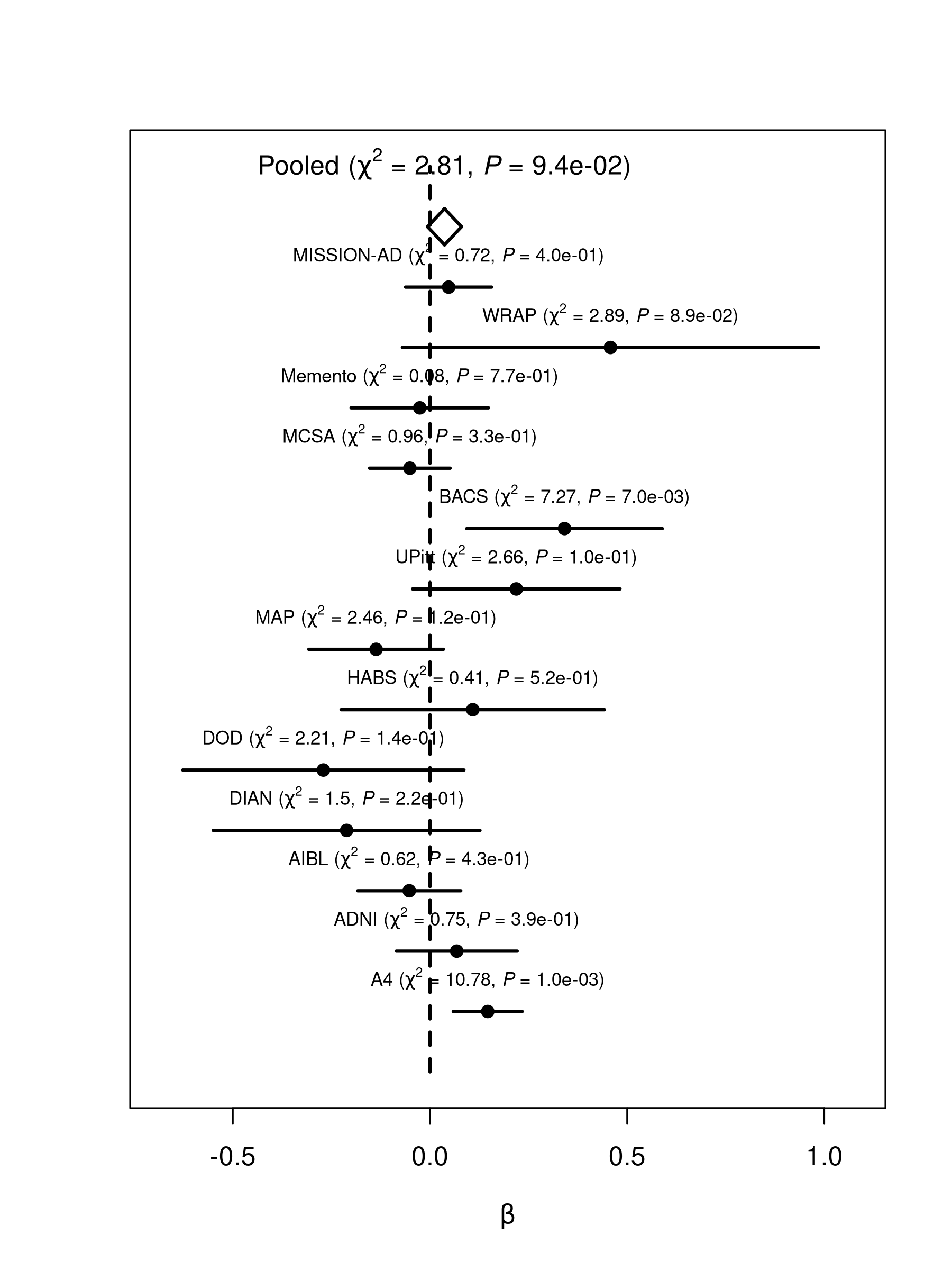 |
| --- |
| **Fig. S19: Forest plot for chr16p.13.3 (rs56081887/chr16:6853159:C:G) demonstrating significance across 13 different cohorts of NHW ancestry**. Forest plot for the SNP previously reported to be significantly associated with increased amyloid levels. The association was assessed for only NHW participants from each cohort. |
